# Synthesis and Auxin-like Activity of Halogenated Alkylphenoxyacetic Acids

**DOI:** 10.3390/ijms27062696

**Published:** 2026-03-16

**Authors:** Stepan V. Vorobyev, Danila V. Mizin, Maria A. Breygina, Ekaterina A. Bykova, Maxim E. Antropov, Boris P. Tonkonogov, Vladimir N. Koshelev

**Affiliations:** 1 Department of Organic Chemistry and Petroleum Chemistry, Faculty of Chemical and Environmental Engineering, Gubkin University, Leninsky Prospect 65, Moscow 119991, Russia; danila.miz@list.ru (D.V.M.); maksantr@vk.com (M.E.A.);; 2 Department of Plant Physiology, Biological Faculty, Lomonosov Moscow State University, Leninskiye Gory 1-12, Moscow 119234, Russia; katebykova.90@mail.ru; 3 Department of Chemistry and Technology of Lubricants and Chemometology, Faculty of Chemical and Environmental Engineering, Gubkin University, Leninsky Prospect 65, Moscow 119991, Russia

**Keywords:** synthetic auxins, alkylphenols, phenoxyacetic acids, selective halogenation, DFT calculations, pollen germination, seed germination, plant growth regulation, plant hormones

## Abstract

Synthetic auxins are widely used nowadays as plant growth regulators and necessary components of media for micropropagation. Hence, the search for and development of novel auxin-like compounds is an important goal at the intersection of chemistry and biology. In this study, we have suggested alkylphenols as starting materials for the preparation of halogenated phenoxyacetic acids, which are well-known synthetic auxins, to decrease their possible phytotoxicity. Alkylphenoxyacetic acids were obtained with good yields, and their selective halogenation was studied. N-halogensuccinimides and molecular bromine in dioxane were shown as suitable reagents since they allowed for *p*-halogenophenoxyacetic acids to be synthesized with high yields. We further investigated the auxin-like activity of several obtained compounds. It was estimated that all of them stimulate tobacco *Nicotiana tabacum* L. pollen germination at concentrations 10^−6^–10^−7^ M with the maximum effect up to 157%. For the most efficient compounds, the germination of tobacco (*Nicotiana tabacum* L.) and corn (*Zea mays*) seeds was studied, as well as seedling growth. The results demonstrate the efficacy of obtained compounds as synthetic auxins, showing that alkylphenols are prospective starting materials for such compounds.

## 1. Introduction

Plant growth and development are regulated by a multi-level system of signaling pathways, in which the hormone auxin plays a key role. It is involved in modulating such fundamental processes as cell division and elongation, apical dominance, formation of lateral and adventitious roots, and development of fruits and reproductive organs [1]. In particular, the nature of the main representative of the auxin family, indole-3-acetic acid (IAA), its synthesis, transport, metabolism, and degradation are discussed in detail in recent review articles emphasizing its central role in the regulation of plant growth and adaptation [2,3]. However, the use of natural auxins in agriculture and other applied phytotechnologies is limited for several reasons: high levels of endogenous regulation, rapid degradation, and conjugation, as well as instability in plant tissues and the inability to ensure a stable, long-lasting effect [4].

Synthetic analogs of auxins, in particular representatives of the phenoxyacetic acid group (2,4-dichlorophenoxyacetic acid (**2,4-D**) and related compounds), have been used for decades as plant growth regulators and herbicides [5]. These substances act as auxin mimics—they can imitate the effect of natural auxins, stimulating the growth of weeds or, conversely, suppressing crop development at high concentrations. However, despite their widespread use and commercial success, serious limitations have been identified: the high resistance of some forms in soil and aquatic environments and insufficient selectivity (i.e., a serious impact on target crops and non-target plants), as well as the risk of accumulation and associated environmental consequences [6,7]. Given these shortcomings, current research is less focused on profound structural changes in each compound, but is instead focused on improving our understanding of the factors that influence the selectivity, distribution, and degradation of phenoxyacetic acid herbicides to improve their safety and sustainability.

One promising direction for the creation of new synthetic auxins is the targeted modification of the phenoxyacetic acid molecule by introducing an alkylphenol fragment. Such structural modification increases lipophilicity and tunes the electronic properties of the aromatic core, thereby facilitating the incorporation of the molecule into the membrane lipid bilayer and influencing its interactions with membrane constituents [8,9]. As demonstrated in model systems, local structural changes within the aromatic fragment and its degree of hydrophobicity correlate with alterations in membrane fluidity and permeability, thereby modulating the transport and intracellular distribution of such derivatives [10].

For phenoxyacetic herbicides, a close relationship has been established between electronic structure, substituent character, and biological activity, as emphasized by recent structure–activity studies on phenoxyacetic acid derivatives [11]. Quantum–chemical calculations and analysis of the herbicide-likeness of substituted phenoxyacetic acids show that bulky alkyl substituents significantly affect the distribution of electron density, frontier orbital parameters, and the ability to interact with the transport inhibitor response 1 (TIR1) receptor, which correlates with the potential for auxin-like activity [12]. In our recent work on the density functional theory (DFT) study of alkylphenolic derivatives of phenoxyacetic acid [13], it was shown that several alkylphenols satisfy the key electron-structural criteria for promising synthetic auxins, which served as the basis for moving on to the stage of targeted synthesis and biological testing.

At the same time, current data on the transport of phenoxyacetic herbicides indicate that these compounds are recognized and transported by the PIN-formed (PIN)-like auxin transporters [14], which shifts the focus towards the search for new structures with an optimized interaction profile with transport and receptor systems. Despite the existence of theoretical and patent developments in the field of phenoxyacetic herbicides, systematic experimental studies of alkylphenolic phenoxyacetic acid series with a detailed comparison of their auxin-like activity remain extremely limited, which creates significant scope for further research.

The development of our previous DFT study of alkylphenolic potential auxins naturally required a transition from theoretical models to a reproducible synthetic route that would allow a series of real compounds for biological testing. Recent reviews on aryloxy- and phenoxy-derivatives of phenols emphasize that subtle modification of the O-substituent and the system of electron-donating/electron-accepting groups in the aromatic nucleus determine the spectrum of biological activity of such compounds, including phytohormone-like effects [15].

For aryloxyacetic acids used as pharmacological agents and herbicides, the most widely used approach is a two-step process: Firstly, construction of a phenoxyacetic acid “platform” should be made by O-alkylation of phenol with chloroacetic acid or its esters (Williamson reaction). Secondly, the subsequent variation in substituents in the aromatic nucleus is usually provided, making it possible to synthesize many biologically active aryloxyacetic acids, including modern multi-target molecules with activity against PPAR receptors and lipid metabolism enzymes [16]. At the same time, approaches to the targeted functionalization of natural phenols are being actively developed: It has been shown that the introduction of additional fragments (carboxylic acids, esters, phenoxyacetic blocks, etc.) via an O-bond significantly improves the solubility, antioxidant properties, and pharmacokinetic parameters of such derivatives [17,18].

Against this background, the strategy of using monomeric phenolic monoterpenoids (thymol, carvacrol, *p*-cymene derivatives) as “natural” building blocks appears particularly interesting. A few studies have shown that their targeted O-functionalization and side-chain modification allow for the fine tuning of lipophilicity, volatility, antioxidant, and antimicrobial properties [19]. For carvacrol and thymol derivatives, semisynthetic routes involving O-alkylation and acylation stages have been successfully implemented, demonstrating the fundamental technological feasibility of such substrates [20]. Our approach is based on the following logical construction: The thymol structure, which has pronounced membrane-protective and antioxidant properties, was chosen as the base, onto which a phenoxyacetic fragment, a carrier of auxin-like activity, is “built up”. As a development of our previous work dedicated to the chemistry of alkylphenols [21,22], in this study the derivatives of methylphenols were obtained in order to evaluate their biological activity.

## 2. Results and Discussion

### 2.1. Chemistry

This work implements a two-stage synthetic route to produce alkylphenolic phenoxyacetic acids. In the first stage, thymol and related alkylphenols are converted into the corresponding thymoxy- and other alkylphenoxyacetic acids by O-alkylation. Alkylphenoxyacetic acids, thus obtained, are further halogenated by an appropriate agent. The alternative route is the usage of halogenated phenols for alkylation reaction. Though it is single-staged, it is not the preferable scheme, since highly toxic dioxins [23,24,25] can be formed during phenols’ halogenation. In order to prevent this side reaction, we decided to apply a two-staged synthetic route and to estimate the selectivity of halogenation reagents at alkylphenoxyacetic acids.

**Figure 1:**
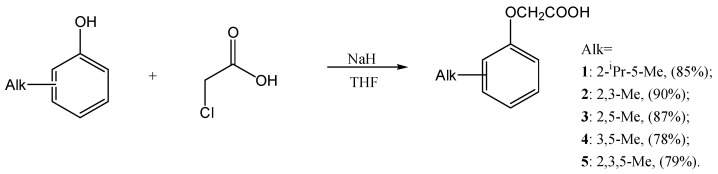
Reaction scheme of alkylphenoxyacetic acid preparation.

The O-alkylation reaction is the well-known one, and many possible ways of its carrying have been proposed. The most widely used one is to conduct the reaction in an aqueous alkali solution, both with and without suitable catalysts. All of these methods, though being accessible, suffer from yield-reducing side reactions [26,27], the most important of which is chloroacetic acid hydrolysis. Nevertheless, we carried out the synthesis of thymoxyacetic acid according to the described method [28], but the yield of the target compound was only 15%. Therefore, for further synthesis, we chose a method using THF as a solvent and sodium hydride as a base [29], also replacing bromoacetic acid with the cheaper chloroacetic acid. As a result, alkylphenoxyacetic acids **1**–**5** were obtained with good yields exceeding 85% in most entries (Figure 1), and the purity of the compounds obtained allowed them to be used in subsequent syntheses without additional purification. It should be noted that, for several obtained compounds (e.g., for **2**, **7** and **9**), the great difference in melting point temperature is observed, compared to that in the literature. There are several possible explanations, including the formation of a polymorph form of the solid compound or an error in the previous compounds’ identification.

**Figure 2:**
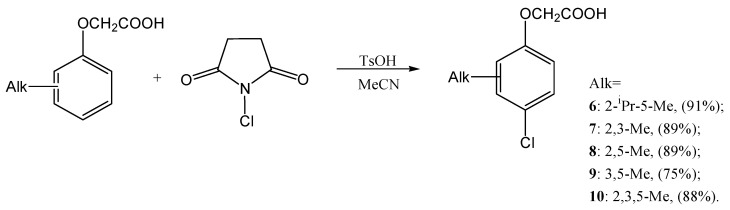
Reaction scheme of chlorination of alkylphenoxyacetic acids.

The second stage of the route involves the targeted introduction of halogen atoms at the *para*-position of aromatic ring of the alkylphenol fragment. It is known that, for this purpose, mild and selective electrophilic reagents of the N-halosuccinimide class might be used: N-chlorosuccinimide (NCS), N-bromosuccinimide (NBS), and N-iodosuccinimide (NIS). In recent years it has been shown that the combination of NCS with organoselenium ligands, organic bases, or other activating additives allows for highly regio- and chemoselective chlorination of phenols and aniline derivatives [30,31], while NBS in mild alcohol solutions provides rapid and selective preparation of *ortho*-bromo-substituted phenols [32]. NIS, in turn, has proven to be an effective source of electrophilic iodine for a wide range of aromatic substrates, including phenols. In particular, the “green” methods using mechanical activation (grinding) or non-aqueous conditions [33,34] are of great interest. Since, according to the literature data, chlorine derivatives have the greatest auxin-like activity, we adapted the well-known technique [35] and synthesized *para*-chlorosubstituted alkylphenoxyacetic acids **6**–**10** using NCS in acetonitrile at room temperature, with *para*-toluenesulfonic acid as a catalyst (Figure 2):

The yields of the target compounds are good and lie in the range of 75–91%; the structures of the compounds have been estimated using spectroscopic methods, and the composition was estimated by elemental analysis. Thus, we have shown the applicability of NCS for the chlorination of phenoxyacetic acids.

**Figure 3:**
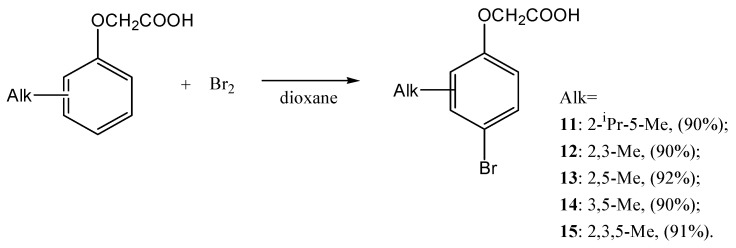
Reaction scheme of bromination of alkylphenoxyacetic acids.

**Figure 4:**
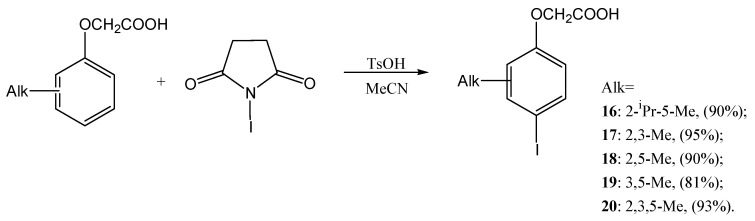
Reaction scheme of iodination of alkylphenoxyacetic acids.

Then, the target reaction was extended to other halosuccinimides. Thymoxyacetic acid was brominated by NBS with a product **11** yield equal to 81%. However, in continuation of our studies on phenols bromination [36], we have studied the possibility of obtaining target compounds using the molecular bromine in dioxane. This method has several advantages, as it does not require a catalyst. Under these conditions, the target compound c was obtained with an even higher yield up to 91%, so the remaining alkylphenoxyacetic acids **12**–**15** were obtained using the bromine–dioxane system. This technique proved to be effective, making it possible to obtain target compounds with yields exceeding 90% (Figure 3):

Finally, the iodination was performed by NIS in a similar way to chlorination, yielding iodoalkylphenoxyacetic acids **16**–**20** with high yields (Figure 4):

Both the corresponding halosuccinimides and bromine in dioxane make it possible to obtain target substances with high *para*-position selectivity (the *ortho*-isomer was not determined; see the structure estimation part); thus, we proposed a method for selective halogenation of phenoxyacetic acids. It should be noted that several target compounds (**7**–**9**, **11**, **16**) are mentioned in the literature [37,38,39]; however, neither spectral data nor elemental analyses (in some cases) are given. In addition, no systematic study of the phenoxyacetic acid halogenation was previously provided. The main method for bromination and iodination was the oxidative one [39,40], requiring a strong oxidant (NaClO_3_ or HIO_3_) and a source of halogen. The halogenated acids were obtained in this case only with moderate yields (usually up to 60%), while we suggest a much more effective system.

For the bioactivity compounds, their stability is a task of a great importance. Synthetic auxins are known for their rapid biodegradation in nature [41,42,43], the main pathways of which include side-chain cleavage and ring hydroxylation [44]. The stability of the studied compounds in solutions are yet to be studied, but we may assume their stability in a solid state. Thus, the NMR spectra of the several compounds (e.g., **11** and **15**) demonstrate no significant changes in two years, if the sample was prepared from a compound’s powder. In addition, NMR samples themselves were repeatedly registrated in a month for compounds **9** and **12**, also demonstrating the absence of destruction.

### 2.2. Structure and Property Estimation

The most informative method for estimating the structure of alkylphenoxyacetic acids and their halogenated derivatives is ^1^H and ^13^C NMR spectroscopy. Modification of the starting alkylphenol with chloroacetic acid leads to the appearance in the ^1^H spectrum of an intense singlet in the region of 4.5 ppm, corresponding to the protons of the methylene group CH_2_. In the ^13^C spectrum, a carbon signal of the same group in the region of 65 ppm is observed, as is a carbon signal of the carboxyl group in a downfield region (See Supplementary Materials).

The change in spectra of halogenated compounds is even more noticeable. Firstly, the number and multiplicity of proton signals in the aromatic ring changes. Secondly, the introduction of various halogens leads to a significant change in the chemical shift in neighboring atoms, especially carbons, due to the “heavy atom effect” [45]. It is characteristic, first of all, for iodine, and reveals in additional shielding of neighboring atoms.

For thymoxyacetic acid **1** and (2,5-dimethylphenoxy)acetic acid **3**, the introduction of a halogen atom into the aromatic ring leads to the disappearance of the AB system of two doublets in the proton spectrum belonging to hydrogen atoms in the third and fourth positions of the aromatic ring. The spectrum in the downfield region is simplified to two singlets corresponding to hydrogen atoms in the third and sixth positions. A similar pattern is observed for (3,5-dimethylphenoxy)acetic acid **4**, the molecule of which is symmetrical, and the proton signal of the aromatic ring is a singlet.

Spectra interpretation for derivatives of (2,3-dimethylphenoxy)acetic acid **2** and (2,3,5-trimethylphenoxy)acetic **5** acids is a more complicated task. In the first case, substitution directed either to the fourth or the sixth positions of the aromatic ring will lead to the appearance in the proton spectrum of two doublets from nearby hydrogens; in the second case, a single hydrogen atom remains in the ring, with the singlet signal. Calculating chemical shift values using quantum–chemical methods can help to assign the peaks properly and to interpret the structure. Based on the recommendations provided by the CHESHIRE CCAT project [46], we selected the DFT B3LYP/6-311+G (2d,p) method, combining gas-phase optimization and a SCRF-solvation (chloroform or DMSO) model for NMR calculations for chlorine-substituted acids **7** and **10**. Theoretical and experimental chemical shifts were then compared, and the agreement between them was estimated by calculation of mean absolute deviation (MAD) and root mean square (RMS) deviation. The MAD and RMS values for the *para*-isomer were lower in both cases. For compound **7**, MAD and RMS were equal to 0.077 and 0.098, respectively, compared to 0.221 and 0.371 for the *ortho*-isomer. For compound **10,** they were equal to 0.123 and 0.134, compared to 0.383 and 0.530 for the *ortho*-isomer. This data also confirms the proceeding of the reaction at *para*-position of the corresponding acid.

Since only a few basis sets are suitable for the heavy atoms, we decided to change the combination of the functionals and basis set for compounds **17** and **20**. According to CHESHIRE CCAT data, the combination of B3LYP/6-31+G (d,p) optimization and BMK/6-311G(d) gives quite accurate results. We used the latter basis set for both optimization and NMR calculation, and the obtained results proved that the substitution proceeds at the *para*-position. When used for bromine and chlorine derivatives **12**, **15** and **7**, **10**, respectively, the MAD and RMS values became larger, but still making it possible to estimate the orientation of the substitution reaction. Further calculations are needed in order to estimate more accurate scaling factors for halogenated phenoxyacetic acids. Calculated data is presented in Table 1.

In order to estimate the applicability of NMR calculations for compounds with heavy atoms, we tried the alternative method for the compound **20**. The PBE0 method was implemented, together with 6-311G(d) functional. The obtained data was scaled according to CHESHIRE CCAT. Though MAD and RMS values were larger than in the previous case, they still made it possible to check the way of substitution. Thus, for the *para*-isomer, they were equal to 0.369 and 0.412, respectively, while for the *ortho*- one, they were 0.418 and 0.569.

Lipophilicity was estimated using online services for the several obtained compounds in order to explain their bioactivity (see the bioactivity study part). Calculated values of logP are presented in Table 2.

It is noteworthy that two different methods gave quite controversial results. For three isomers of chloro-dimethylphenoxyacetic acids, the same values of logP were calculated by ProteinIQ, which seems to be counter-intuitive. When the chlorine atom is replaced with bromine or iodine, the increase in lipophilicity is observed. The most significant changes take place for thymol derivatives, due to its isopropyl group. The lipophilicity of the target compounds was compared to that of commercial synthetic auxin, **2,4-D**, indicating the greater values than for the **2,4-D**.

### 2.3. Bioactivity Study

To assess the potential biological activity of substances in plants, it is customary to use biotests, the most convenient of which are germinating pollen and germinating seeds [47,48,49]. A suspension of short-living culture of *Nicotiana tabacum* L. pollen germinating in vitro was used as a simple and sensitive model system [50]. Based on the data from our previous study [13], the highest auxin-like activity was expected for compound **9**, so its effect was studied in a wide range of concentrations (10^−4^–10^−11^ M). We found that, at low concentrations (10^−8^–10^−11^ M), it did not stimulate pollen germination, while at high concentrations (10^−4^–10^−5^ M), compound **9** had a noticeable inhibitory effect. The optimal concentration was 10^−6^ M, at which the relative increase in germination efficiency reached +70,8 ± 4,7%. Based on the obtained data, we chose a narrow concentration range (10^−5^–10^−7^ M) to test substances **6**–**11**, **13**, and **16**. The results of the pollen tests are presented in Table 3.

Generally, germination inhibition or no effect were observed for all studied compounds at a concentration of 10^−5^ M. The most pronounced stimulating effect for most compounds was found at 10^−6^ M (significant for **6**, **9**–**11**, **13** and **16**), while three of them (**7**, **10** and **16**) showed the same or even greater stimulation at 10^−7^ M. In this case, lower concentrations (10^−8–^10^−11^ M) were additionally tested, but germination did not change significantly. We also reproduced the effect of the well-known synthetic auxin **2,4-D**, which stimulated pollen germination at a concentration of 10^−6^ M.

**Figure 5:**
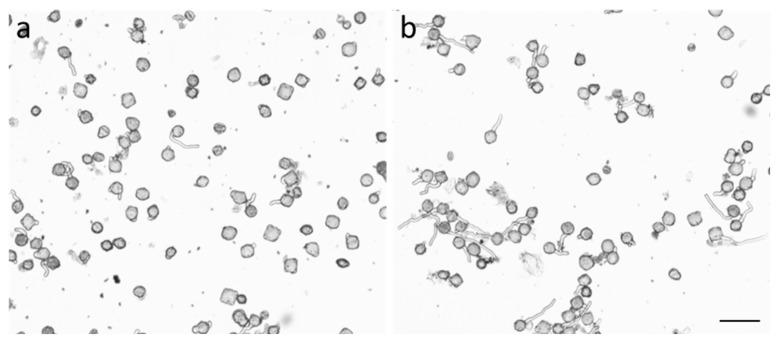
The effect of compound **16** on *Nicotiana tabacum* pollen germination efficiency (an example of images used for pollen counting): (**a**)—control suspension, (**b**)—suspension treated with substance **16** (10^−7^ M). Scale bar—100 µm.

It is noteworthy that substance **16**, which is iodine derivative, showed the greatest stimulating activity in both effective concentrations (Table 3, Figure 5). This data does not fit into the previously established trend, according to which the auxin-like activity of compounds decreases in a row of chloro-bromine-iodine atoms as substituents [11,51,52]. This discrepancy may be because earlier studies did not use the pollen test. The mechanism and possible reason for such high sensitivity of pollen to substance **16** remains to be studied, but one of the hypotheses is a noticeable change in the lipophilicity of this compound in comparison with known phenoxyacetic acids. Indeed, compound **16** demonstrates the lipophilicity that is an order of magnitude higher than that of **2,4-D**.

The stimulation of pollen germination by a number of the studied compounds indicates pronounced auxin-like activity. The stimulating effect of natural auxins on pollen germination or/and pollen tube growth has been described for several species, including plum (*Prunus domestica*) [53], pear (*Pyrus pyrifolia*) [54], and *Petunia hybrida* [55]. This is consistent with the idea that in vivo pollen tubes grow along an auxin concentration gradient in styles, as has been shown, in particular, for tobacco pistils [56]. The compounds with the highest activity according to the pollen test were further studied in a more complex model system of germinating tobacco and corn seeds and growing seedlings.

The concentration of 10^−5^ M in experiments with all substances was generally inhibitory for pollen; therefore, it was used not in all cases for growing seeds, but only where lower toxicity was expected—for substances **7** and **16**. No significant inhibition of seed germination was observed. In all cases, 10^−5^ M had a negative or insignificant effect on the growth processes in seedlings. Significant stimulation of tobacco seed germination was observed for substances **9** and **16** at 10^−6^ M; we did not find any reliable effect on corn seeds. The reaction of corn and tobacco seedlings to the effects of the substances was largely the opposite: Substance **7** at 10^−6^ M inhibited the growth of the above-ground part of tobacco seedlings; for corn, there was no reliable effect, while substance **8** strongly stimulated growth.

Thus, some substances increased germination or shoot growth efficiency for both large corn seeds, which are characterized by prolonged heterotrophic nutrition (**8**), and small tobacco seeds with a rapid transition to autotrophic nutrition (**8**, **9** and **16**). As expected, small seeds exhibit higher sensitivity to exogenous hormone-like substances. The results of the plant growth test are summarized in Table 4, Table 5 and Table 6.

The shoot elongation in seedlings, as well as cell growth, is one of the most typical manifestations of natural auxin action [57], which in vivo counteracts the action of the antagonist hormone, ethylene. While ethylene indicates stress and the need for shoot protection, auxin stimulates normal seedling development [58]. The obtained data on the different sensitivity of seedlings to auxin-like substances (Table 5 and Table 6) confirms the literature data that the sensitivity to exogenous auxins varies among seedlings of different plant species. Significant stimulation of the above-ground part elongation is detected in maize, which is one of the species most sensitive to auxins [59]. Protoplasts from maize coleoptile were used to develop test systems for determining auxin sensitivity, such as the hormone-induced increase in cytosolic Ca^2+^ and H^+^ concentration [60]. At the same time, whole seedlings largely use the hormones found in the seed, and therefore exhibit less sensitivity to exogenous substances than isolated protoplasts.

The role of auxin in the transition of seeds from dormancy to germination is controversial. Auxins are widely used to stimulate growth processes in low doses [61], but it has been observed that increasing concentrations, conversely, shift the equilibrium toward seed dormancy [62]. The data obtained on tobacco (Table 4) can be compared with the data previously published for natural auxin (IAA): 1000 mg/L auxin solution markedly decreased germination while concentrations of 10 and 100 mg/L were stimulating [61]. Recently, it was discovered that “auxin-induced” seed dormancy, which is observed at high concentrations of the hormone, is due to an increased abscisic acid signaling in the presence of auxin [63]. According to current concepts, the three phytohormones (IAA, abscisic acid and jasmonates) act synergistically to suppress seed germination, as their high concentrations signal unfavorable conditions [64]. Thus, obtained data on the effect of the studied substances at 10^−5^ M on seed germination and seedling growth is consistent with the complex effects of natural auxin and other hormones on this process. Some synthetic auxins (such as substance **16**) are less toxic at high concentrations than natural ones. A potential explanation is the inability of such molecules to interact with natural hormones found in seeds. As for the stimulation of tobacco seed germination by low concentrations of the studied substances, this reflects the general trend towards higher sensitivity of small seeds to exogenous phytohormones.

## 3. Materials and Methods

### 3.1. Chemical Synthesis

For the chemical synthesis, purification, structural analysis, and bioactivity studies, reagents were purchased from local suppliers (Ekos-1 and Reachem, Moscow, Russia) and Merck KGaA (Darmstadt, Germany), and used according to the instructions.

Analytical thin-layer chromatography (TLC) was performed on ALUGRAM Xtra SIL G UV254 (Macherey-Nagel, Düren, Germany) sheets to monitor the reaction progress and to verify the purity of the tested compounds. The elution system employed was a mixture of ethyl acetate and *n*-hexane in a 2:1 ratio. The visualization was carried out with UV light at 254 nm.

Based on the glass capillarity method, melting points were measured using a melting point device Stuart SMP30 apparatus (Bibby Scientific Ltd., Staffordshire, UK).

The structures of compounds were confirmed by recording and examining the IR, ^1^H-NMR, and ^13^C-NMR spectra. FTIR spectra was recorded on an Agilent Cary 660 spectrometer (Agilent Technologies Inc., Santa Clara, CA, USA) equipped with an attenuated total reflectance (ATR) accessory (ZnSe crystal), averaging 32 scans per measurement. NMR spectra (^1^H at 300 MHz, ^13^C at 75 MHz) were acquired on a Bruker Avance II 300 spectrometer (Bruker Corporation, Billerica, MA, USA) using DMSO-d6 or CDCl_3_ as the solvent and tetramethylsilane (TMS) as the internal standard. The chemical shift (δ) values are described in parts per million (ppm). Elemental analysis was performed on a Vario MicroCube analyzer (Elementar Analysensysteme GmbH, Langenselbold, Germany).

General procedure for the synthesis of intermediate compounds **1**–**5**

To a solution of 0.01 mol of corresponding phenol in 20 mL of tetrahydrofuran 0.025 mol of sodium hydride (60% oil dispersion) was added. After hydrogen release, 0.01 mol of chloroacetic acid was added, and the resulting mixture was heated with stirring for 6 h. The reaction mass was then rotary evaporated to 1/3 of the starting volume, diluted with water and acidified with 10% hydrochloric acid. The formed precipitate was filtered off and dried on air, resulting in the correspondent alkylphenoxyacetic acid. It can be used without additional purification.

*(2-isopropyl-5-methylphenoxy)acetic acid* **1** (thymoxyacetic acid)

Yield 85%, m.p. 145–147 °C (lit. 146 °C [28])

^1^H NMR (CDCl_3_, δ, ppm, ^3^J_HH_, Hz): 1.24 (d, 6H, CH(CH_3_)_2_, ^3^J_HH_ = 7.14); 2.34 (s, 3H, ArCH_3_); 3.37 (sep, 1H, CH(CH_3_)_2_, ^3^J_HH_ = 7.14); 4.71 (s, 2H, OCH_2_COOH); 6.59 (s, 1H, ArH); 6.83 (d, 1H, ArH, ^3^J_HH_ = 7.68); 7.15 (d, 1H, ArH, ^3^J_HH_ = 7.68).

*(2,3-dimethylphenoxy)acetic acid* **2**

Yield 90%, m.p. 189–191 °C (lit. 95–97 °C [65])

^1^H NMR (DMSO-d_6_, *δ*, ppm, ^3^*J*_HH_, Hz): 2.10 (s, 3H, ArCH_3_); 2.20 (s, 3H, ArCH_3_); 4.62 (s, 2H, OCH_2_COOH); 6.64 (d, 1H, ArH, ^3^*J*_HH_ = 8.06); 6.75 (d, 1H, ArH, ^3^*J*_HH_ = 7.32); 6.98 (t, 1H, ArH, ^3^*J*_HH_ = 8.05).

*(2,5-dimethylphenoxy)acetic acid* **3**

Yield 87%, m.p. 157–158 °C (lit. 153–155 °C [66])

^1^H NMR (DMSO-d_6_, *δ*, ppm, ^3^*J*_HH_, Hz): 2.11 (s, 3H, ArCH_3_); 2.21 (s, 3H, ArCH_3_); 4.63 (s, 2H, OCH_2_COOH); 6.61–6.65 (m, 2H, ArH); 6.98 (d, 1H, ArH; ^3^*J*_HH_ = 7.31); 12.88 (bs, 1H, COOH).

*(3,5-dimethylphenoxy)acetic acid* **4**

Yield 78%, m.p. 98–100 °C (lit. 100–104 °C [67])

^1^H NMR (CDCl_3_, *δ*, ppm, ^3^*J*_HH_, Hz): 2.31 (s, 6H, ArCH_3_); 4.66 (s, 2H, OCH_2_COOH); 6.57 (s, 2H, ArH); 6.69 (s, 1H, ArH).

*(2,3,5-trimethylphenoxy)acetic acid* **5**

Yield 79%, m.p. 115–117 °C (lit. 123–125 °C [68])

^1^H NMR (DMSO-d_6_, δ, ppm, ^3^J_HH_, Hz): 2.04 (s, 3H, ArCH_3_); 2.14 (s, 3H, ArCH_3_); 2.17 (s, 3H, ArCH_3_); 4.59 (s, 2H, OCH_2_COOH); 6.47 (s, 1H, ArH); 6.57 (s, 1H, ArH); 12.84 (bs, 1H, COOH).

*General procedure for the synthesis of chlorinated compounds* **6**–**10**

To a solution of 0.01 mol of corresponding alkylphenoxyacetic acid in 20 mL of dry acetonitrile, 0.01 mol of N-chlorosuccinimide and 0.01 mol of *para*-toluenesulfonic acid were added, and the resulting mixture was stirred for 12 h. The reaction mass was then rotary evaporated to 1/3 of the starting volume and diluted with a large volume of water. The formed precipitate was filtered off and dried on air, resulting in the correspondent chloroalkylphenoxyacetic acid.

*(4-chloro-2-isopropyl-5-methylphenoxy)acetic acid* **6**

Yield 85%, m.p. 131–132 °C (lit. 138 °C [69]).

^1^H NMR (DMSO-d_6_, δ, ppm, ^3^J_HH_, Hz): 1.14 (d, 6H, CH(CH_3_)_2_, ^3^J_HH_ = 6.95); 2.23 (s, 3H, ArCH_3_); 3.23 (sep, 1H, CH(CH_3_)_2_, ^3^J_HH_ = 6.95); 4.67 (s, 2H, OCH_2_COOH); 6.83 (s, 1H, ArH); 7.13 (s, 1H, ArH).

^13^C NMR (DMSO-d_6_, δ, ppm): 19.96 (ArCH_3_); 22.79 (CH(CH_3_)_2_); 26.67 (CH(CH_3_)_2_); 65.42 (OCH_2_COOH); 114.90; 125.40; 126.60; 133.66; 136.53; 154.06; 170.51.

IR, cm^−1^: 1741 (ν C=O). Elemental analysis found C, 59.31; H, 6.31. Calculated for C_12_H_15_ClO_3_: C, 59.39; H, 6.23.

*(4-chloro-2,3-dimethylphenoxy)acetic acid* **7**

Yield 89%, m.p. 162–164 °C (lit. 117 °C [37]).

^1^H NMR (DMSO-d_6_, *δ*, ppm, ^3^*J*_HH_, Hz): 2.16 (s, 3H, ArCH_3_); 2.25 (s, 3H, ArCH_3_); 4.65 (s, 2H, OCH_2_COOH); 6.71 (d, part of AB-system, 1H, ArH, ^3^*J*_HH_ = 8.78); 7.16 (d, part of AB-system, 1H, ArH, ^3^*J*_HH_ = 8.78).

^13^C NMR (DMSO-d_6_, *δ*, ppm): 13.05 (ArCH_3_); 16.94 (ArCH_3_); 65.61 (OCH_2_COOH); 111.10; 125.95; 126.70; 127.33; 135.27; 154. 93; 170.60.

IR, cm^−1^: 1736 (ν C=O). Elemental analysis found C, 55.85; H, 5.30. Calculated for C_10_H_11_ClO_3_: C, 55.96; H, 5.17.

*(4-chloro-2,5-dimethylphenoxy)acetic acid* **8**

Yield 89%, m.p. 103–104 °C (lit. 118 °C [37]).

^1^H NMR (CDCl_3_, *δ*, ppm, ^3^*J*_HH_, Hz): 2.22 (s, 3H, ArCH_3_); 2.31 (s, 3H, ArCH_3_); 4.66 (s, 2H, OCH_2_COOH); 6.58 (s, 1H, ArH); 7.12 (s, 1H, ArH).

^13^C NMR (CDCl_3_, *δ*, ppm): 15.54 (ArCH_3_); 20.04 (ArCH_3_); 65.41 (OCH_2_COOH); 113.97; 126.49; 126.65; 131.20; 134.02; 154.26; 173.92.

IR, cm^−1^: 1759 (ν C=O). Elemental analysis found C, 55.86; H, 5.25. Calculated for C_10_H_11_ClO_3_: C, 55.96; H, 5.17.

*(4-chloro-3,5-dimethylphenoxy)acetic acid* **9**

Yield 75%, m.p. 122–125 °C (lit. 100–103 °C [38]).

^1^H NMR (CDCl_3_, *δ*, ppm, ^3^*J*_HH_, Hz): 2.37 (s, 6H, ArCH_3_); 4.67 (s, 2H, OCH_2_COOH); 6.68 (s, 2H, ArH); 10.01 (bs, 1H, COOH).

^13^C NMR (CDCl_3_, *δ*, ppm): 20.95 (ArCH_3_); 64.93 (OCH_2_COOH); 114.64; 127.75; 137.53; 155.24; 174.23.

IR, cm^−1^: 1755 (ν C=O). Elemental analysis found C, 55.85; H, 5.27. Calculated for C_10_H_11_ClO_3_: C, 55.96; H, 5.17.

*(4-chloro-2,3,5-trimethylphenoxy)acetic acid* **10**

Yield 88%, m.p. 139–141 °C.

^1^H NMR (CDCl_3_, *δ*, ppm, ^3^*J*_HH_, Hz): 2.11 (s, 3H, ArCH_3_); 2.20 (s, 6H, Ar(CH_3_)_2_); 4.45 (s, 2H, OCH_2_COOH); 6.41 (s, 1H, ArH).

^13^C NMR (CDCl_3_, *δ*, ppm): 12.62 (ArCH_3_); 17.07 (ArCH_3_); 21.10 (ArCH_3_); 65.81 (OCH_2_COOH); 111.80; 124.67; 127.32; 133.40; 135.64; 154.00; 170.94.

IR, cm^−1^: 1742 (ν C=O). Elemental analysis found C, 57.74; H, 5.75. Calculated for C_11_H_13_ClO_3_: C, 57.78; H, 5.73.

*General procedure for the synthesis of brominated compounds* **11**–**15**

To a solution of 0.01 mol of corresponding alkylphenoxyacetic acid in 20 mL of dry dioxane, 0.01 mol of bromine was added, and the resulting mixture was stirred for 6 h. The reaction mass was then rotary evaporated to 1/3 of the starting volume and diluted with a large volume of water. The formed precipitate was filtered off and dried on air, resulting in the correspondent bromoalkylphenoxyacetic acid.

*(4-bromo-2-isopropyl-5-methylphenoxy)acetic acid* **11**

Yield 90%, m.p. 127–129 °C (lit. 132–135 °C [39]).

^1^H NMR (CDCl_3_, δ, ppm, ^3^J_HH_, Hz): 1.21 (d, 6H, CH(CH_3_)_2_, ^3^*J*_HH_ = 6.58); 2.34 (s, 3H, ArCH_3_); 3.31 (m, 1H, CH(CH_3_)_2_); 4.68 (s, 2H, OCH_2_COOH); 6.62 (s, 1H, ArH); 7.35 (s, 1H, ArH); 8.55 (bs, 1H, COOH).

^13^C NMR (CDCl_3_, δ, ppm): 22.53 (CH(CH_3_)_2_); 22.80 (ArCH_3_); 26.65 (CH(CH_3_)_2_); 65.22 (OCH_2_COOH); 113.95; 116.99; 130.29; 135.70; 137.23; 153.88; 173.97.

IR, cm^−1^: 1738 (ν C=O). Elemental analysis found C, 50.08; H, 5.35. Calculated for C_12_H_15_BrO_3_: C, 50.19; H, 5.27.

*(4-bromo-2,3-dimethylphenoxy)acetic acid* **12**

Yield 90%, m.p. 143–145 °C.

^1^H NMR (CDCl_3_, δ, ppm, ^3^J_HH_, Hz): 2.22 (s, 3H, ArCH_3_); 2.30 (s, 3H, ArCH_3_); 4.51 (s, 2H, OCH_2_COOH); 6.46 (d, part of AB-system, 1H, ArH, ^3^*J*_HH_ = 8.78); 7.24 (d, part of AB-system, 1H, ArH, ^3^*J*_HH_ = 8.78); 8.00 (bs, 1H, COOH).

^13^C NMR (CDCl_3_, δ, ppm): 13.21 (ArCH_3_); 19.87 (ArCH_3_); 65.66 (OCH_2_COOH); 110.60; 117.19; 127.66; 129.51; 137.27; 155.12; 170.94.

IR, cm^−1^: 1738 (ν C=O). Elemental analysis found C, 46.29; H, 4.37. Calculated for C_10_H_11_BrO_3_: C, 46.36; H, 4.28.

*(4-bromo-2,5-dimethylphenoxy)acetic acid* **13**

Yield 92%, m.p. 98–99 °C.

^1^H NMR (CDCl_3_, δ, ppm, ^3^J_HH_, Hz): 2.22 (s, 3H, ArCH_3_); 2.34 (s, 3H, ArCH_3_); 4.66 (s, 2H, OCH_2_COOH); 6.60 (s, 1H, ArH); 7.31 (s, 1H, ArH).

^13^C NMR (CDCl_3_, δ, ppm): 15.45 (ArCH_3_); 22.88 (ArCH_3_); 65.29 (OCH_2_COOH); 113.89; 116.45; 126.82; 134.32; 135.96; 154.93; 173.78.

IR, cm^−1^: 1749 (ν C=O). Elemental analysis found C, 46.33; H, 4.31. Calculated for C_10_H_11_BrO_3_: C, 46.36; H, 4.28.

*(4-bromo-3,5-dimethylphenoxy)acetic acid* **14**

Yield 90%, m.p. 138–140 °C.

^1^H NMR (DMSO-d_6_, δ, ppm, ^3^J_HH_, Hz): 2.29 (s, 6H, ArCH_3_); 4.63 (s, 2H, OCH_2_COOH); 6.76 (s, 2H, ArH).

^13^C NMR (DMSO-d_6_, δ, ppm): 23.97 (ArCH_3_); 64.86 (OCH_2_COOH); 115.08; 118.26; 138.96; 156.86; 170.50.

IR, cm^−1^: 1746 (ν C=O). Elemental analysis found C, 46.31; H, 4.30. Calculated for C_10_H_11_BrO_3_: C, 46.36; H, 4.28.

*(4-bromo-2,3,5-trimethylphenoxy)acetic acid* **15**

Yield 91%, m.p. 147–149 °C.

^1^H NMR (DMSO-d_6_, δ, ppm, ^3^J_HH_, Hz): 2.13 (s, 3H, ArCH_3_); 2.25 (s, 3H, ArCH_3_); 2.29 (s, 3H, ArCH_3_); 4.14 (s, 2H, OCH_2_COOH); 6.60 (s, 1H, ArH).

^13^C NMR (DMSO-d_6_, δ, ppm): 13.38 (ArCH_3_); 20.58 (ArCH_3_); 24.53 (ArCH_3_); 68.88 (OCH_2_COOH); 112.75; 117.71; 124.06; 134.82; 136.28; 156.33; 171.35.

IR, cm^−1^: 1739 (ν C=O). Elemental analysis found C, 48.45; H, 4.86. Calculated for C_11_H_13_BrO_3_: C, 48.37; H, 4.80.

*General procedure for the synthesis of iodinated compounds* **16**–**20**

To a solution of 0.01 mol of corresponding alkylphenoxyacetic acid in 20 mL of dry acetonitrile, 0.01 mol of N-iodosuccinimide and 0.01 mol of *para*-toluenesulfonic acid were added, and the resulting mixture was stirred for 12 h. The reaction mass was then rotary evaporated to 1/3 of the starting volume and diluted with a large volume of water. The formed precipitate was filtered off and dried on air, resulting in the correspondent iodoalkylphenoxyacetic acid.

*(4-iodo-2-isopropyl-5-methylphenoxy)acetic acid* **16**

Yield 90%, m.p. 121–123 °C (lit. 126–127 °C [40]).

^1^H NMR (CDCl_3_, δ, ppm, ^3^J_HH_, Hz): 1.20 (d, 6H, CH(CH_3_)_2_, ^3^*J*_HH_ = 6.95); 2.37 (s, 3H, ArCH_3_); 3.28 (sep, 1H, CH(CH_3_)_2_, ^3^*J*_HH_ = 6.95); 4.66 (s, 2H, OCH_2_COOH); 6.62 (s, 1H, ArH); 7.58 (s, 1H, ArH).

^13^C NMR (CDCl_3_, δ, ppm): 22.59 (CH(CH_3_)_2_); 26.62 (ArCH_3_); 28.02 (CH(CH_3_)_2_); 65.07 (OCH_2_COOH); 91.93; 113.15; 136.85; 137.46; 139.53; 155.04; 173.63.

IR, cm^−1^: 1738 (ν C=O). Elemental analysis found C, 43.01; H, 4.60. Calculated for C_12_H_15_IO_3_: C, 43.13; H, 4.52.

*(4-iodo-2,3-dimethylphenoxy)acetic acid* **17**

Yield 95%, m.p. 169–171 °C.

^1^H NMR (CDCl_3_, δ, ppm, ^3^J_HH_, Hz): 2.11 (s, 3H, ArCH_3_); 2.23 (s, 3H, ArCH_3_); 4.38 (s, 2H, OCH_2_COOH); 6.22 (d, part of AB-system, 1H, ArH, ^3^*J*_HH_ = 8.78); 7.39 (d, part of AB-system, 1H, ArH, ^3^*J*_HH_ = 8.78).

^13^C NMR (CDCl_3_, δ, ppm): 13.11 (ArCH_3_); 25.05 (ArCH_3_); 65.26 (OCH_2_COOH); 91.98; 110.96; 126.70; 135.77; 139.97; 155.81; 170.32.

IR, cm^−1^: 1742 (ν C=O). Elemental analysis found C, 39.18; H, 3.69. Calculated for C_10_H_11_IO_3_: C, 39.24; H, 3.62.

*(4-iodo-2,5-dimethylphenoxy)acetic acid* **18**

Yield 90%, m.p. 140–142 °C.

^1^H NMR (CDCl_3_, δ, ppm, ^3^J_HH_, Hz): 2.21 (s, 3H, ArCH_3_); 2.38 (s, 3H, ArCH_3_); 4.66 (s, 2H, OCH_2_COOH); 6.63 (s, 1H, ArH); 7.57 (s, 1H, ArH).

^13^C NMR (CDCl_3_, δ, ppm): 15.24 (ArCH_3_); 28.00 (ArCH_3_); 65.11 (OCH_2_COOH); 91.20; 113.00; 127.04; 139.72; 140.72; 156.00; 173.70.

IR, cm^−1^: 1751 (ν C=O). Elemental analysis found C, 39.15; H, 3.70. Calculated for C_10_H_11_IO_3_: C, 39.24; H, 3.62.

*(4-iodo-3,5-dimethylphenoxy)acetic acid* **19**

Yield 81%, m.p. 179–182 °C.

^1^H NMR (CDCl_3_, δ, ppm, ^3^J_HH_, Hz): 2.26 (s, 6H, ArCH_3_); 4.39 (s, 2H, OCH_2_COOH); 6.52 (s, 2H, ArH).

^13^C NMR (CDCl_3_, δ, ppm): 29.57 (ArCH_3_); 64.73 (OCH_2_COOH); 97.84; 113.31; 142.69; 157.30; 170.49.

IR, cm^−1^: 1746 (ν C=O). Elemental analysis found C, 39.17; H, 3.70. Calculated for C_10_H_11_IO_3_: C, 39.24; H, 3.62.

*(4-iodo-2,3,5-trimethylphenoxy)acetic acid* **20**

Yield 93%, m.p. 159–161 °C.

^1^H NMR (CDCl_3_, δ, ppm, ^3^J_HH_, Hz): 2.23 (s, 3H, ArCH_3_); 2.36 (s, 3H, ArCH_3_); 2.41 (s, 3H, ArCH_3_); 4.51 (s, 2H, OCH_2_COOH); 6.52 (s, 1H, ArH).

^13^C NMR (CDCl_3_, δ, ppm): 13.53 (ArCH_3_); 26.49 (ArCH_3_); 30.39 (ArCH_3_); 65.63 (OCH_2_COOH); 100.05; 110.92; 123.72; 139.38; 140.92; 155.69; 171.04.

IR, cm^−1^: 1749 (ν C=O). Elemental analysis found C, 41.21; H, 4.18. Calculated for C_11_H_13_IO_3_: C, 41.27; H, 4.09.

### 3.2. Computational Details

Quantum–chemical calculations were performed using Gaussian 16 software [70], and the results were visualized by GaussView 6.0.16 [71]. Initially, the geometry of compounds **7** and **10** was optimized, using gas-phase calculation at the B3LYP/6-31+G(d,p) level. NMR chemical shifts were further calculated by B3LYP/6-311+G(2d,p) with the default (SCRF) solvation model and then scaled according to CHESHIRE CCAT method [46]. B3LYP/6-311+G (2d,p) was chosen due to its tight root mean square deviation value. Theoretical and experimental chemical shifts were compared, and the agreement between them was estimated by calculation of mean absolute deviation (MAD) and root mean square (RMS) deviation. For heavier atoms the B3LYP/6-311G* (gas-phase) method was applied for geometry optimization, and BMK/6-311G* was applied for NMR calculations (SCRF solvent model). For compound **20**, an additional method was used, B3LYP/6-311G* (gas-phase) for geometry optimization, together with PBE0/6-311G* for NMR calculations (SCRF solvent model).

The lipophilicity of the target compounds was calculated by two different online sources: Virtual Computational Chemistry Laboratory [72] and Molecular descriptors by ProteinIQ (https://proteiniq.io/, accessed on 6 March 2026).

### 3.3. Pollen Germination Test

Plants of *Nicotiana tabacum* L. var. Petit Havana SR1 were grown in a climatic chamber in controlled conditions (25 °C, 16h light) in vermiculite. For pollen collection, the anthers were removed from the flowers on the eve of opening and dried at 25 °C for 2 days, after which the pollen was collected with a specially equipped vacuum cleaner. Dry pollen was stored at −20 °C. Pollen germination efficiency was assessed after 1 h of cultivation at 25 °C in standard medium containing 0.3 M sucrose, 1.6 mM H_3_BO_3_, 3 mM Ca(NO_3_)_2_, 0.8 mM MgSO_4_, and 1 mM KNO_3_ in 25 mM MES-Tris buffer, pH 5.8 at 2 mg pollen/mL [73]. Before cultivation, pollen was pre-hydrated in a humid atmosphere for 2 h. Germinated pollen was fixed with 2% paraformaldehyde in 50 mM Na-phosphate buffer, pH 7.4 for minimum 30 min at 4 °C. Between 500 and 900 pollen grains from each suspension were analyzed by light microscopy at magnification ×100–×400.

The studied compounds were added into the nutrient medium in the appropriate concentration. For each variant, a control experiment was performed without the addition of a substance.

The results were examined using the Fiji ImageJ 2.17.0 program. Since it was not possible to conduct all the experiments in one day, for a more objective assessment, germination of the control suspension was always evaluated on the same day as the experimental one. To compare experiments conducted on different days, the relative change in germination (in percent) was introduced, which is calculated using the following formula:

$$p=\frac{|\max\left(b\right)-K|}{K}\times 100\%,$$

where *p* is the relative increase in germination (in%); *b* is pollen germination (in%); *max*(*b*) is the maximum average germination that the substance could provide in one concentration or another; and *K* is the average germination in the control (in %).

### 3.4. Seeds Germination Test

For the experiment of morphometric parameter estimation in seedlings, pre-sterilized seeds of corn (*Zea mays*) and tobacco (*Nicotiana tabacum* L.) were used. The seeds were sterilized using the broad-spectrum fungicide “Maxim”. A sterilized paper filter was placed in a glass Petri dish and moistened with a solution of the test substance in a certain concentration. Next, the seeds were placed in this Petri dish and covered with a lid, and, if necessary, wrapped with cling film to reduce evaporation of the liquid. Due to evaporation, the liquid level in the dishes was replenished with the appropriate solution. If necessary, the lids were removed as the plants grew. Tap water was used for the control dishes. The cups were incubated in controlled conditions (25 °C, 16h light). For each concentration of the test substance, 3 Petri dishes were placed, each containing at least 20 corn seeds or 35 tobacco seeds.

On day 7, morphometric parameters were estimated from the plants. The length of the longest root in the root system, the length of the hypocotyl (the internode from the root neck to the cotyledons, for tobacco), the length of the longest leaf (only for corn), and germination (the number of germinated seeds) were measured. All the results were presented in the form of a relative increase in one parameter or another, which was calculated using the formula given above.

### 3.5. Statistical Analysis

Results in the tables are presented as means ± standard errors. In pollen and seed germination experiments, the sample sizes were small (1 Petri dish = 1 sample); typically, 3–6 dishes were counted, so we used the Mann–Whitney test for small samples. For shoot and root length counts, the samples were larger (10–30 plants), so we checked for normal distribution using the Shapiro–Wilk test. In case of a positive result, we used Student’s *t*-test for normal samples.

## 4. Conclusions

A method for halogenated alkylphenoxyacetic acid preparation was suggested; nine novel compounds were obtained. We revealed that N-halosuccinimides and molecular bromine in dioxane make it possible to obtain target derivatives of phenoxyacetic acids with high yields and high selectivity. The structures of the synthesized compounds were estimated by IR and NMR spectroscopy. In addition, quantum–chemical calculations were used to assign NMR peaks with the structure. It was shown that BMK functional in combination with Pople’s split-valence basis set 6-311G* fits well for bromine- and iodine-containing molecule NMR calculations. In bioactivity investigation, it was found that target compounds exhibit auxin-like activity, stimulating pollen germination, with the maximum effect for (4-iodo-2-isopropyl-5-methylphenoxy)acetic acid **16**. This compound has the highest lipophilicity among the studied ones, which probably influences its bioactivity. Further studies are warranted to clarify its mechanism of action. In addition, the effect on seed germination for several target compounds was evaluated, demonstrating that (4-chloro-3,5-dimethylphenoxy)acetic acid **9** and **16** stimulate tobacco seed germination; corn seedling growth is accelerated by (4-chloro-2,3-dimethylphenoxy)acetic acid **7** and (4-chloro-2,5-dimethylphenoxy)acetic acid **8**. New substances that have demonstrated auxin-like activity in bioassays can be further tested for applied use in large-scale laboratory studies and, further, field experiments.

## Figures and Tables

**Table 1:** MAD and RMS values for BMK/6-311G(d) NMR calculation in comparison to observed peaks.

	Values for Studied Compounds			
Substitution	*para*-		*ortho*-	
Compound	MAD	RMS	MAD	RMS
**7**	0.177	0.191	0.289	0.339
**10**	0.252	0.277	0.520	0.581
**12**	0.233	0.259	0.413	0.486
**15**	0.233	0.245	0.504	0.638
**17**	0.111	0.126	0.326	0.3984
**20**	0.110	0.133	0.252	0.366

**Table 2:** Lipophilicity (logP) values for studied compounds.

Compound	logP Values	
	VCCLab ALOGPS 2.1	ProteinIQ Molecular Descriptors
**6**	3.61	3.24
**7**	3.09	2.42
**8**	3.07	2.42
**9**	3.09	2.42
**10**	2.87	2.73
**11**	3.53	3.24
**13**	2.81	2.53
**16**	3.83	3.19
**2,4-D**	2.82	2.46

**Table 3:** The effect of studied compounds on *Nicotiana tabacum* pollen germination efficiency.

Compound	Changes in Germination Efficiency Relative to the Control, %		
	10^−5^ M	10^−6^ M	10^−7^ M
**6**	−23.4 ± 0.4 **	+14.7 ± 1.6 *	−14.9 ± 0.9 **
**7**	−43.5 ± 1.0	+7.0 ± 2.1	+97.7 ± 0.9 **
**8**	−47.5 ± 1.1 **	+59.1 ± 2.9	−7.9 ± 1.1
**9**	−40.3 ± 3.0 *	+70.8 ± 4.7 *	+15.9 ± 2.4
**10**	−4.1 ± 1.8	+21.7 ± 1.9 **	+36.6 ± 6.1 *
**11**	−28.6 ± 0.1 **	+18.1 ± 0.8 **	+9.2 ± 1.6
**13**	−34.5 ± 1.1	+42.4 ± 2.5	+19.3 ± 2.1
**16**	−66.2 ± 1.7 **	+157.6 ± 1.2 **	+155.1 ± 1.9 **
**2,4-D**	+0.7 ± 0.8	+44 ± 2.3 **	+10.7 ± 1.5

**—significant difference from control, Mann–Whitney test, *p* < 0.01. *—significant difference from control, Mann–Whitney test, *p* < 0.05.

**Table 4:** The effect of studied compounds on seed germination efficiency.

	Changes in Seed Germination Efficiency Relative to Control, %			
Plant	*Nicotiana tabacum*		*Zea mays*	
Compound	10^−5^ M	10^−6^ M	10^−5^ M	10^−6^ M
**7**	−24.4 ± 4.0	+36.7 ± 11.0	−6.9 ± 0.1	+3.6 ± 0.1
**8**	-	−5.0 ± 2.0	-	+3.6 ± 0.2
**9**	-	+40.0 ± 2.0 **	-	+14.3 ± 1.0
**16**	+14.6 ± 2.0	+73.3 ± 13.0 **	0.0 ± 0.1	−10.7 ± 2.0

**—significant difference from control, Mann–Whitney test, *p* < 0.01.

**Table 5:** The effect of studied compounds on the root length in seedlings.

	Change in the Length of the Longest Root Relative to the Control, %			
Plant	*Nicotiana tabacum*		*Zea mays*	
Compound	10^−5^ M	10^−6^ M	10^−5^ M	10^−6^ M
**7**	−70.2 ± 7.7 **	−7.8 ± 0.3	−15.7 ± 1.4	+13.0 ± 1.0
**8**	-	+7.5 ± 0.3	-	−5.3 ± 0.4
**9**	-	+5.2 ± 0.2	-	+13.8 ± 1.0
**16**	−16.6 ± 0.6 **	+2.0 ± 0.1	−13.1 ± 0.9	−2.1 ± 0.2

**—significant difference from control, Student’s *t*-test, *p* < 0.01.

**Table 6:** The effect of studied compounds on the shoot length in seedlings.

	Change in the Length of the Hypocotyl/Coleoptile Relative to the Control, %			
Plant	*Nicotiana tabacum*		*Zea mays*	
Compound	10^−5^ M	10^−6^ M	10^−5^ M	10^−6^ M
**7**	−57.0 ± 4.7 **	−26.4 ± 1.4 **	−8.7 ± 0.7	+12.3 ± 0.9
**8**	-	−10.7 ± 0.6	-	+48.1 ± 3.9 **
**9**	-	−8.4 ± 0.3	-	+2.0 ± 0.2
**16**	−9.5 ± 0.3	−9.5 ± 0.5	−18.2 ± 1.3	+3.2 ± 0.3

**—significant difference from control, Student’s *t*-test, *p* < 0.01.

## Data Availability

The original contributions presented in this study are included in the article/Supplementary Material. Further inquiries can be directed to the corresponding authors.

## Supplementary Materials

The following supporting information can be downloaded at: https://www.mdpi.com/article/10.3390/ijms27062696/s1, spectra of the synthesized compounds **1**–**20**.
