# Supplementary material for: Synthesis and Auxin-like Activity of Halogenated Alkylphenoxyacetic Acids
Source: Int J Mol Sci. 2026 Mar 16;27(6):2696. doi: 10.3390/ijms27062696 (PMC13026768; doi:10.3390/ijms27062696)
Supplement: Supplementary file 1 [file ijms-27-02696-s001.zip › ijms-4179935-supplementary.pdf]

Spectral data for compounds **1-20**

(2-isopropyl-5-methylphenoxy)acetic acid **1**

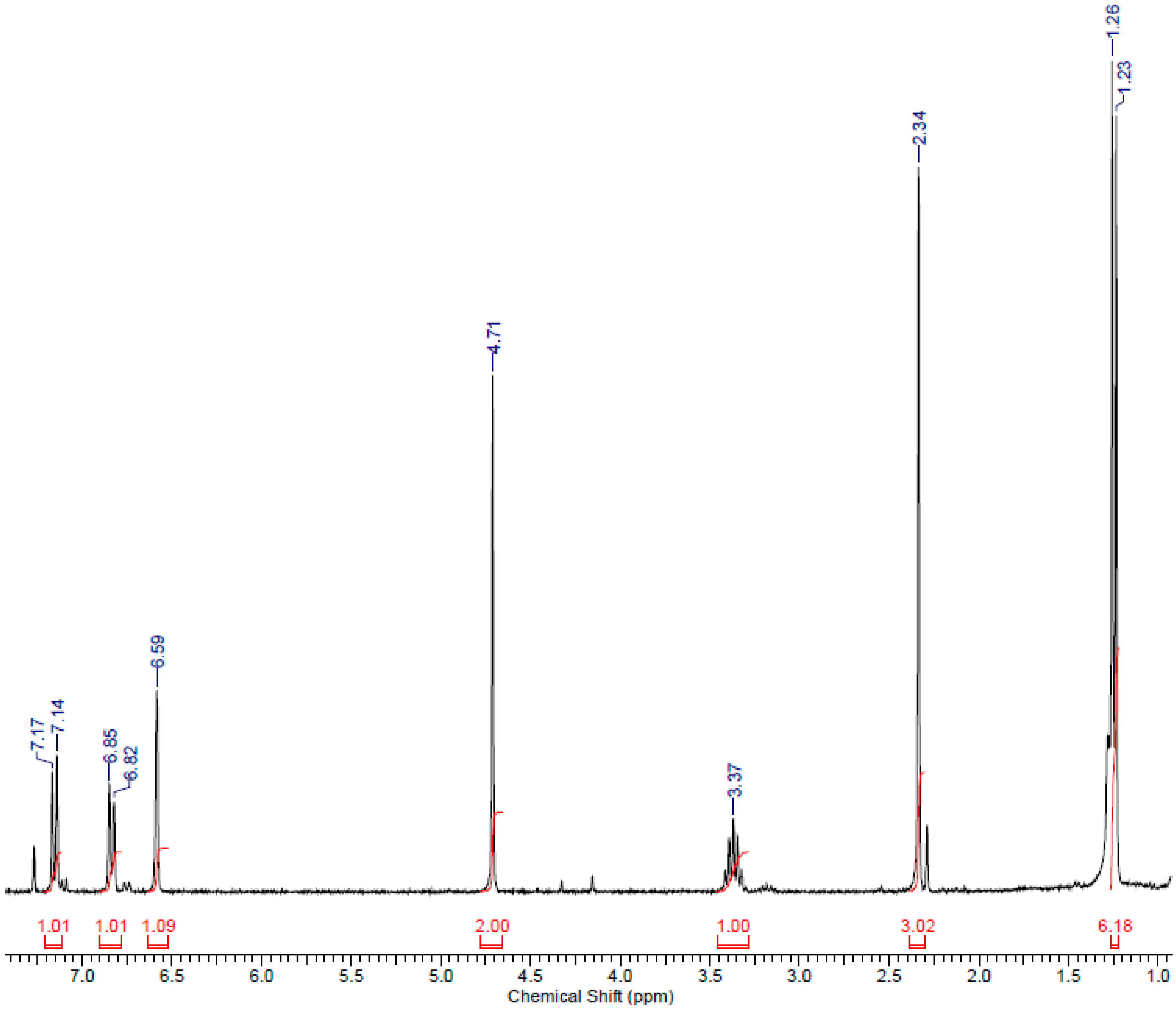

(2,3-dimethylphenoxy)acetic acid **2**

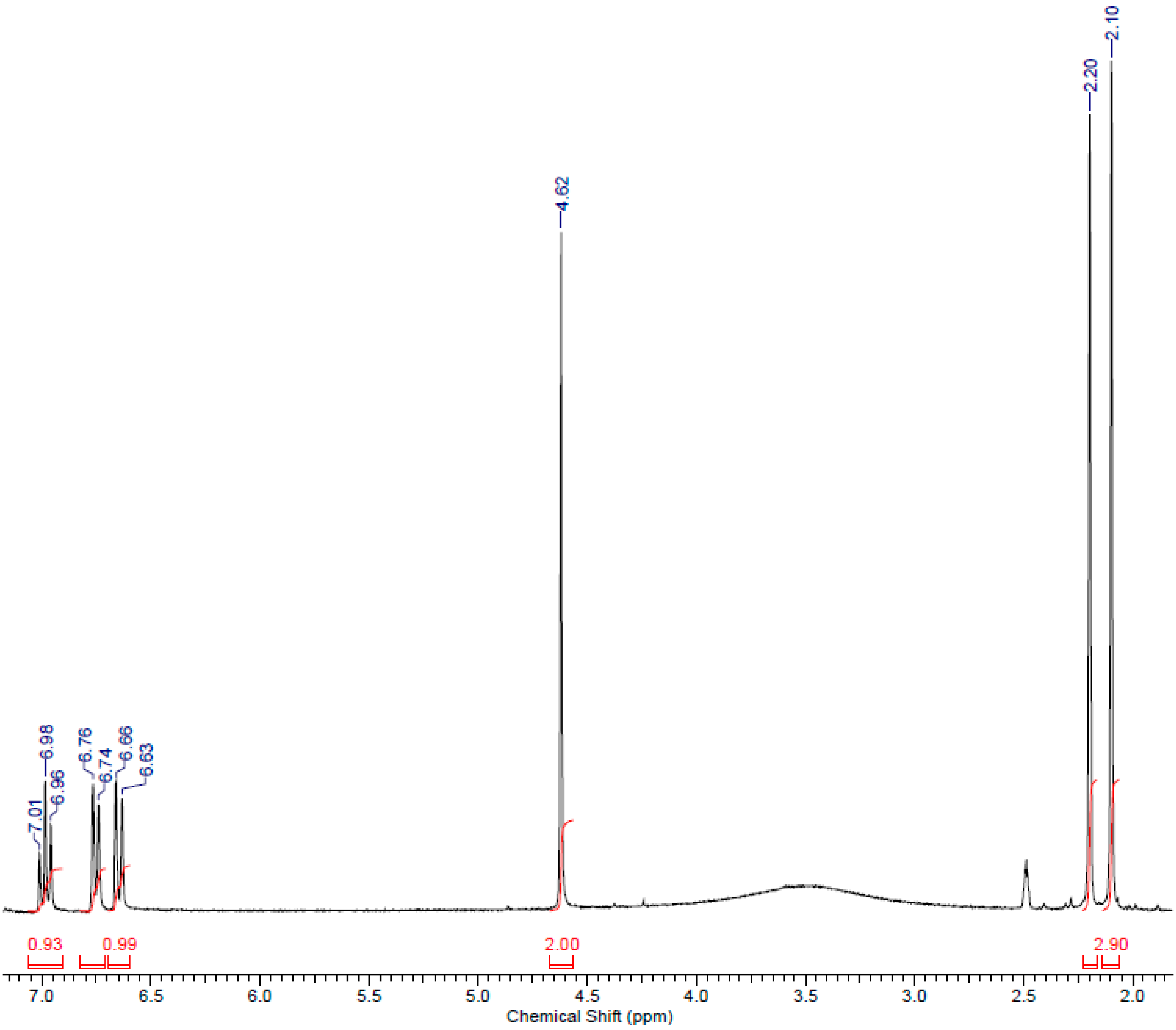

(2,5-dimethylphenoxy)acetic acid **3**

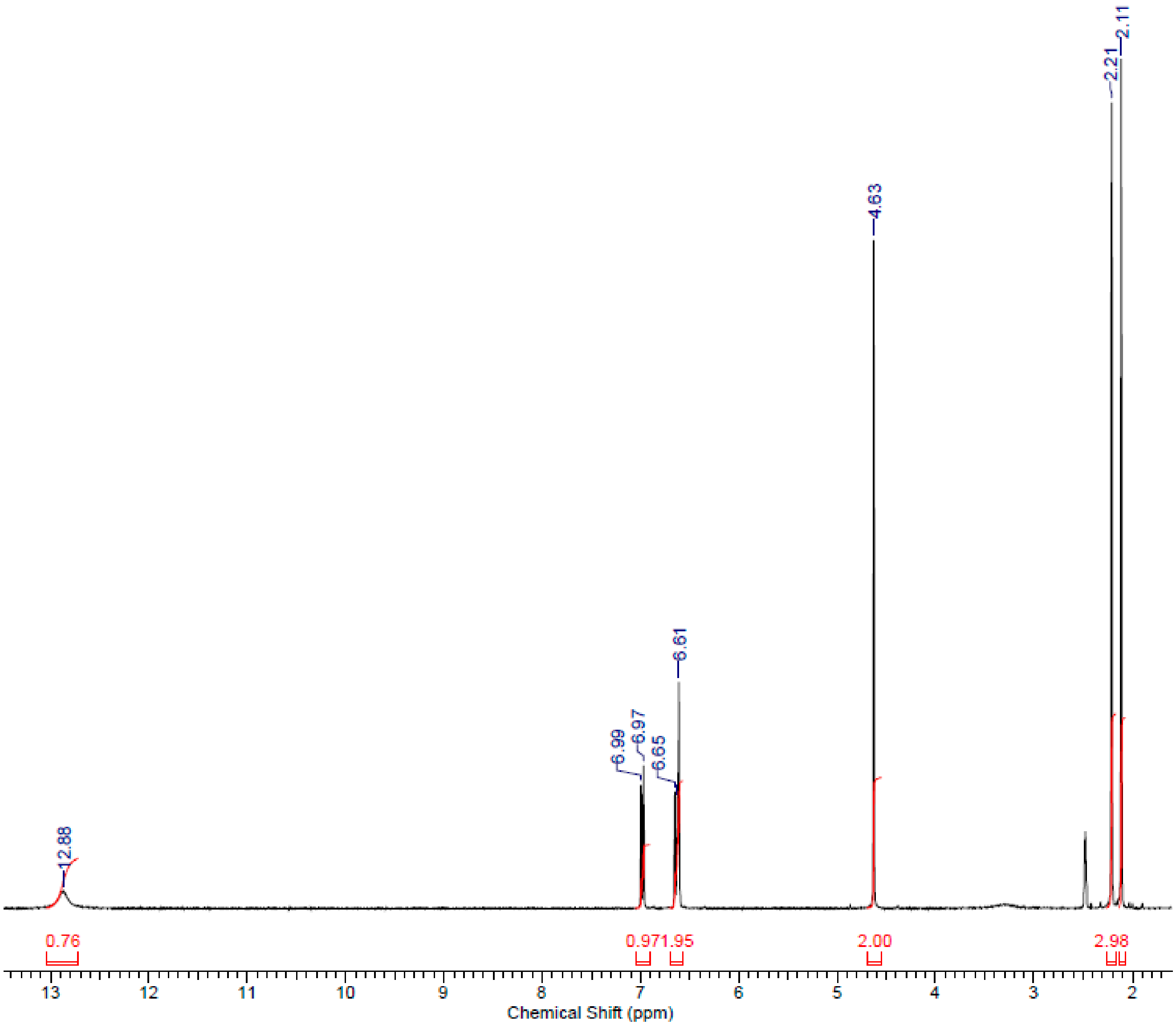

(3,5-dimethylphenoxy)acetic acid **4**

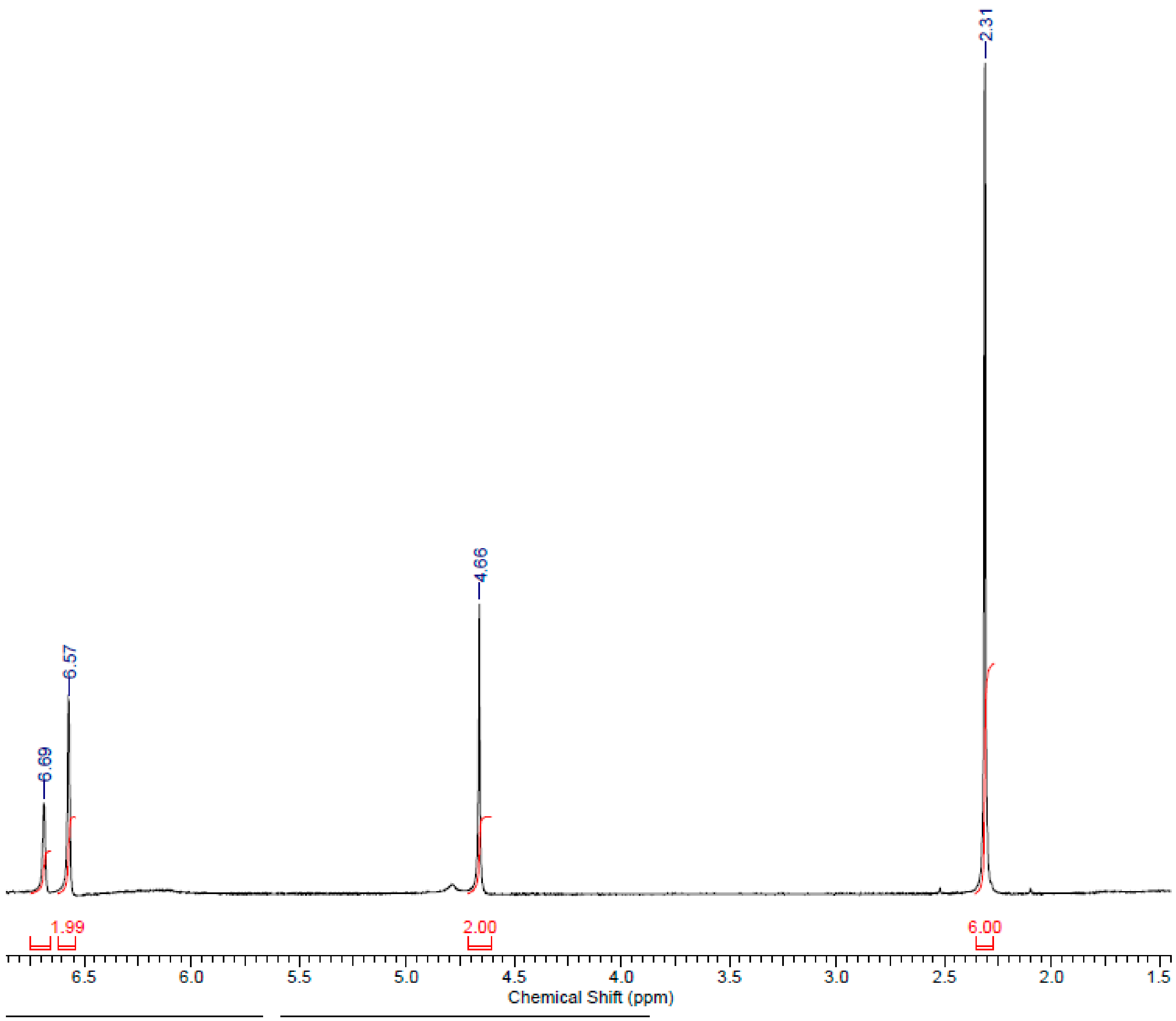

(2,3,5-trimethylphenoxy)acetic acid **5**

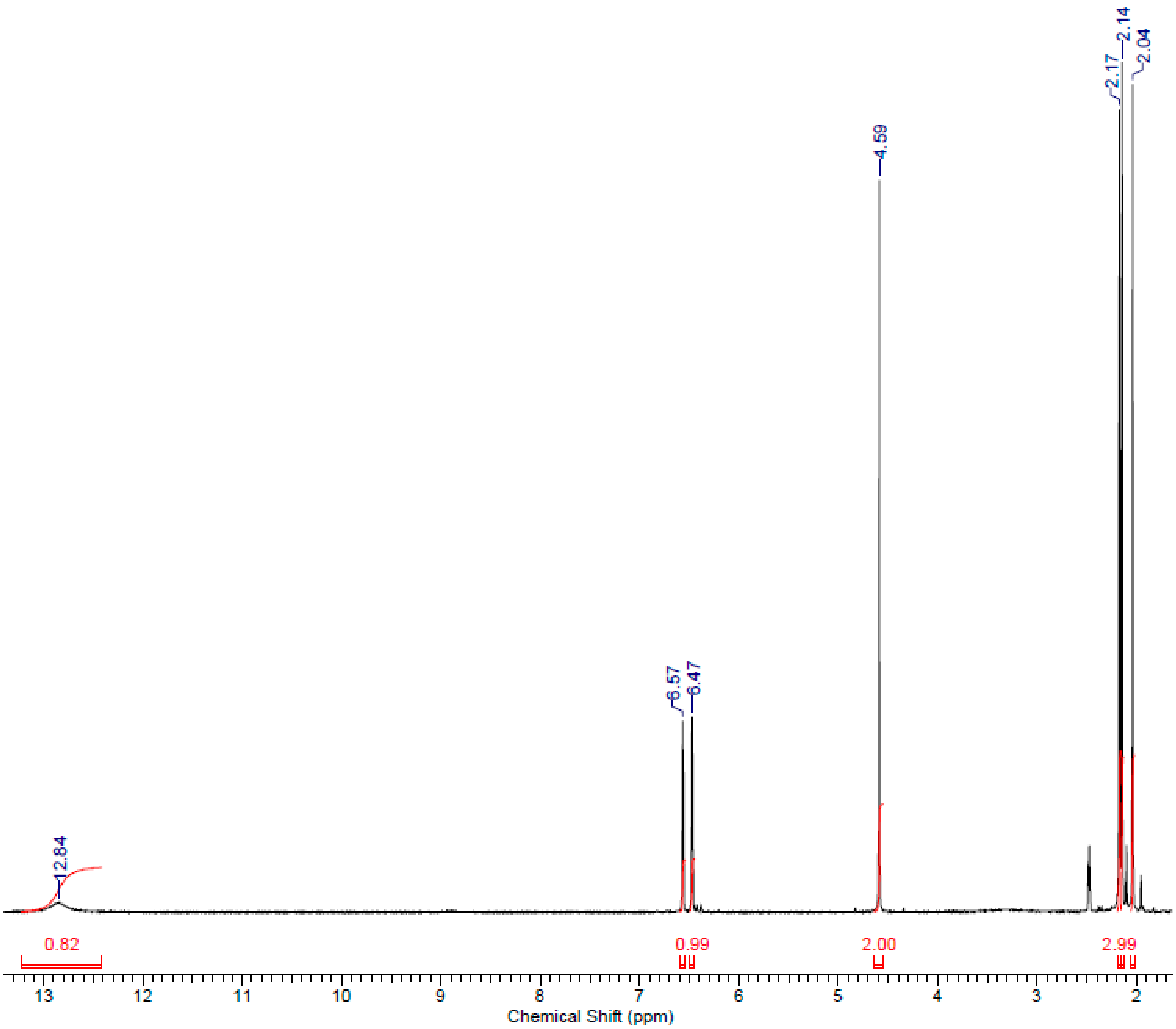

(4-chloro-2-isopropyl-5-methylphenoxy)acetic acid **6**

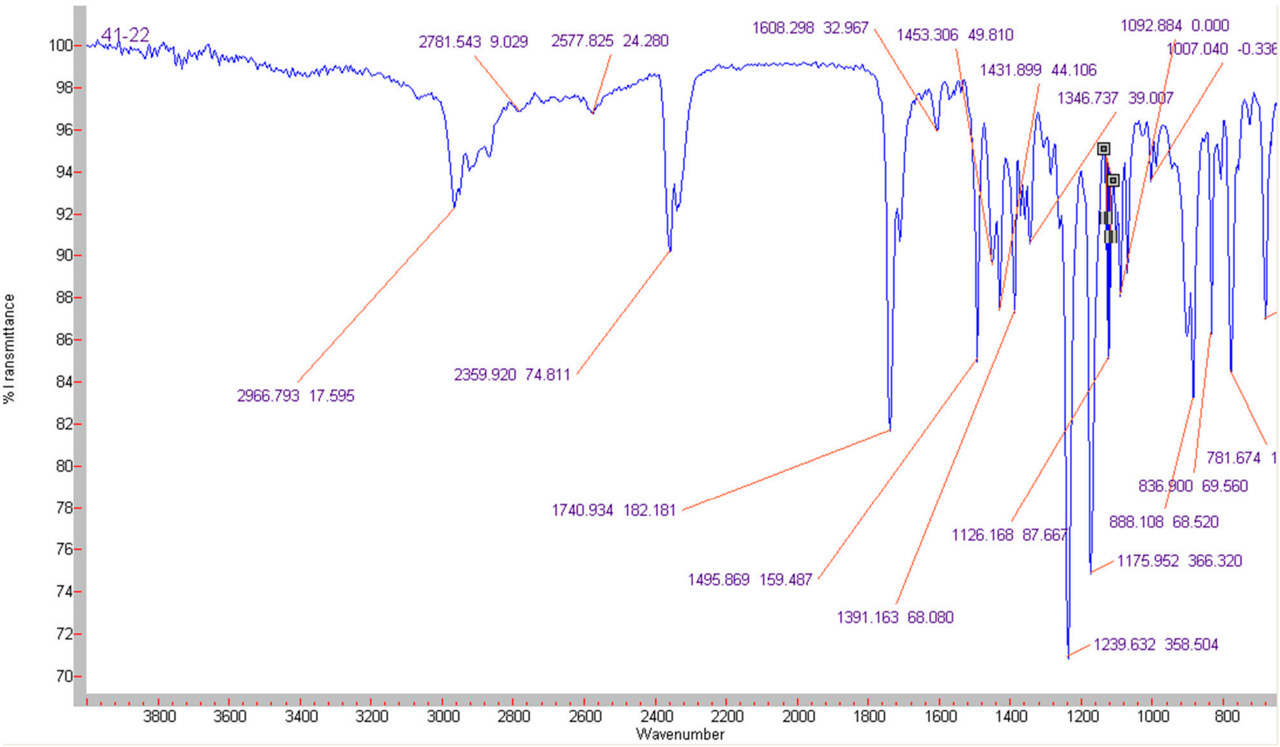

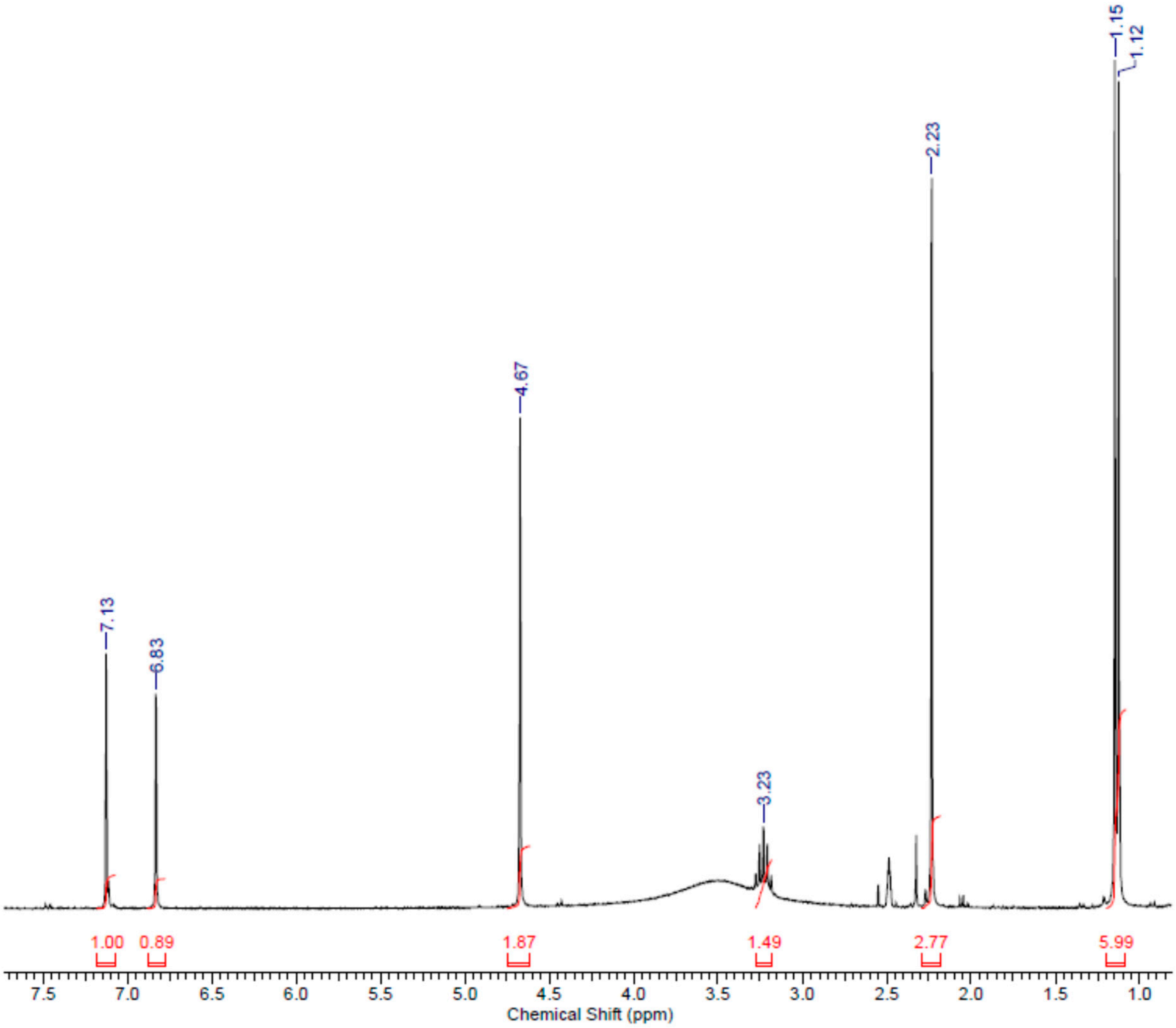

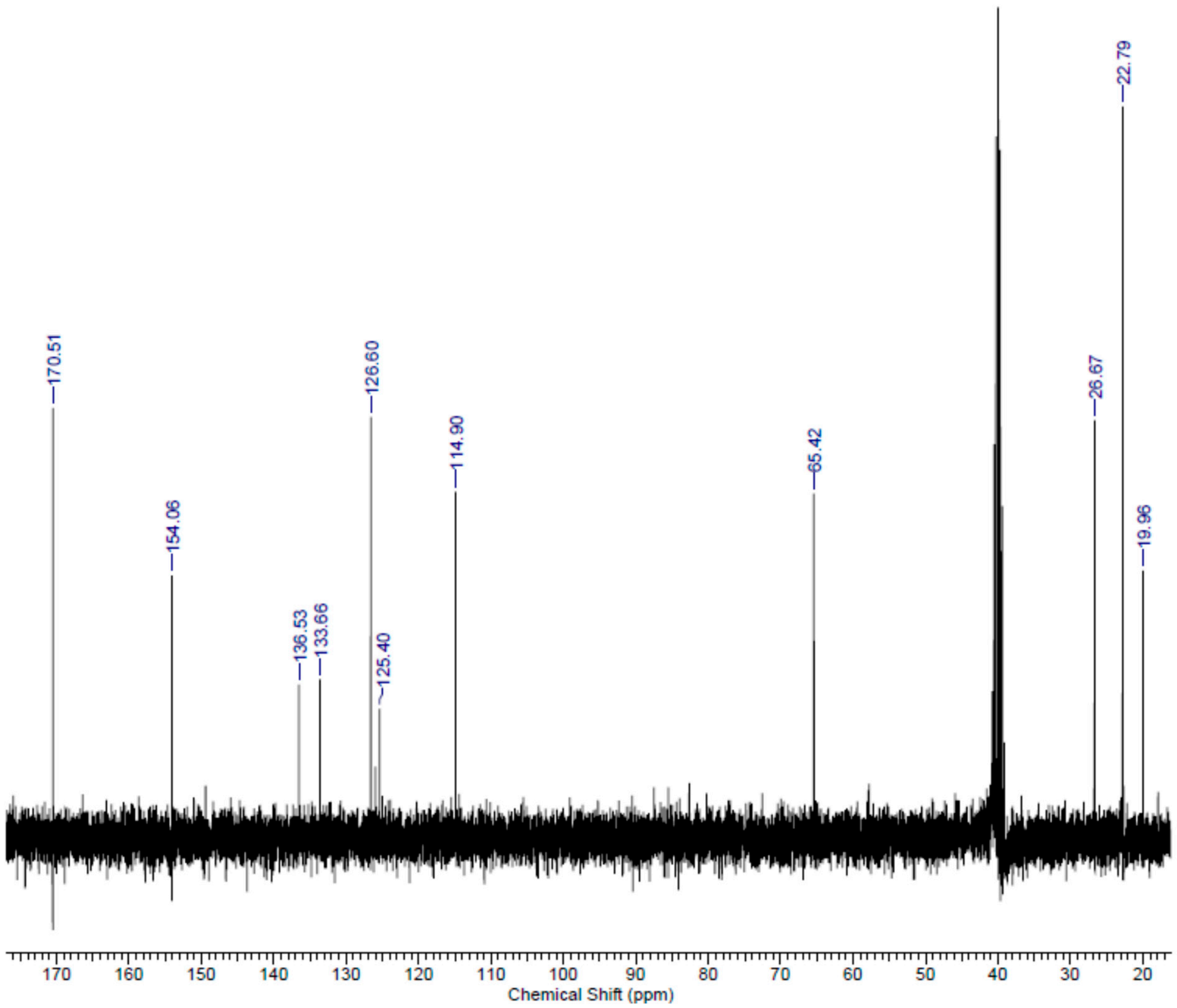

(4-chloro-2,3-dimethylphenoxy)acetic acid **7**

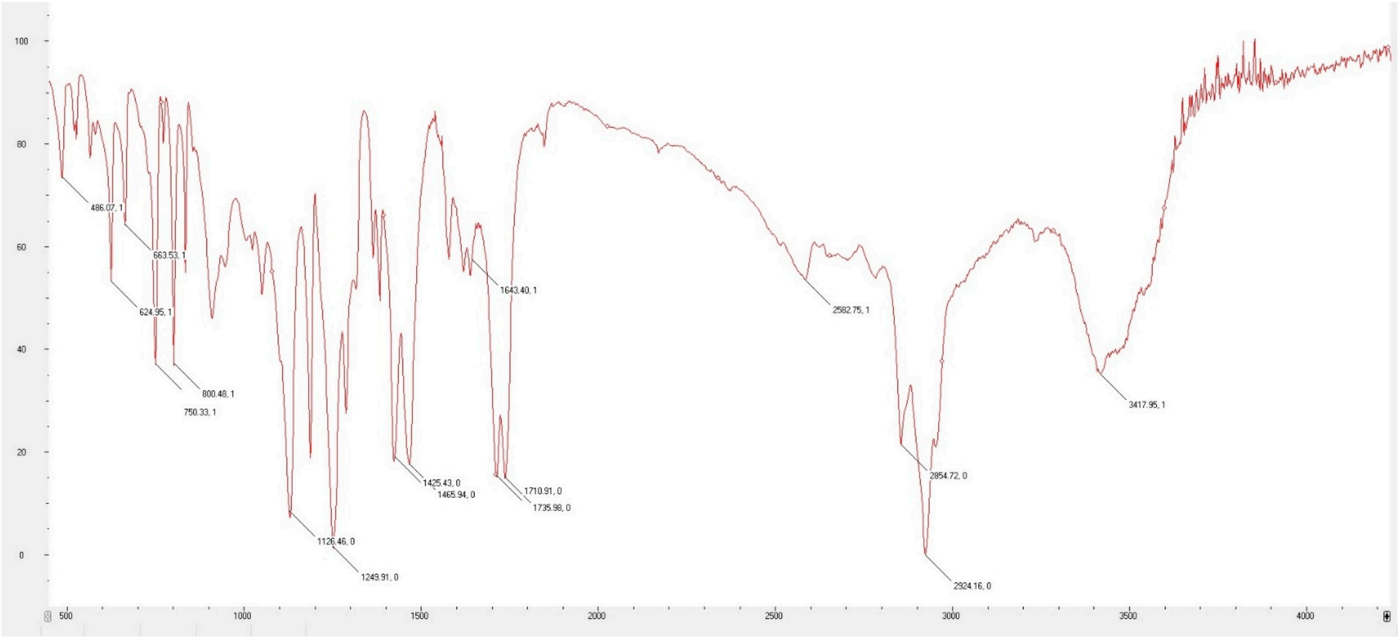

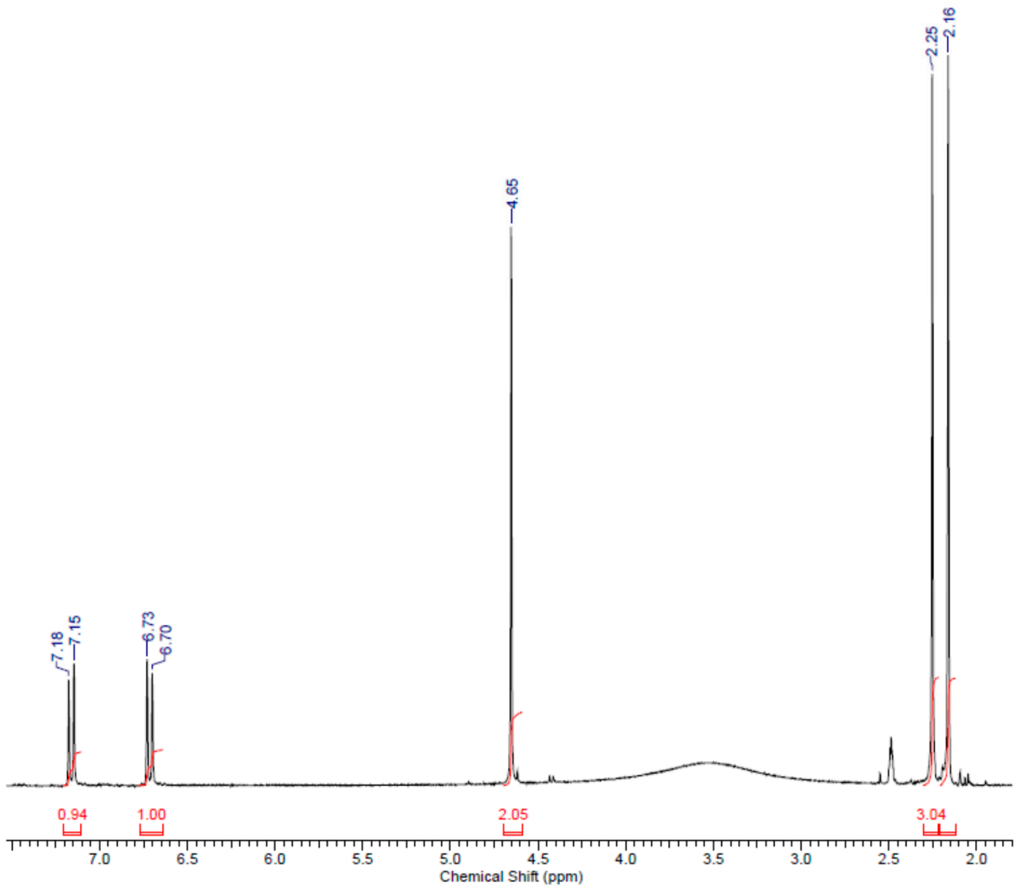

7.18
7.15
6.73
6.70
4.65
2.25
2.16
0.94
1.00
2.05
3.04
7.0
6.5
6.0
5.5
5.0
4.5
4.0
3.5
3.0
2.5
2.0
Chemical Shift (ppm)

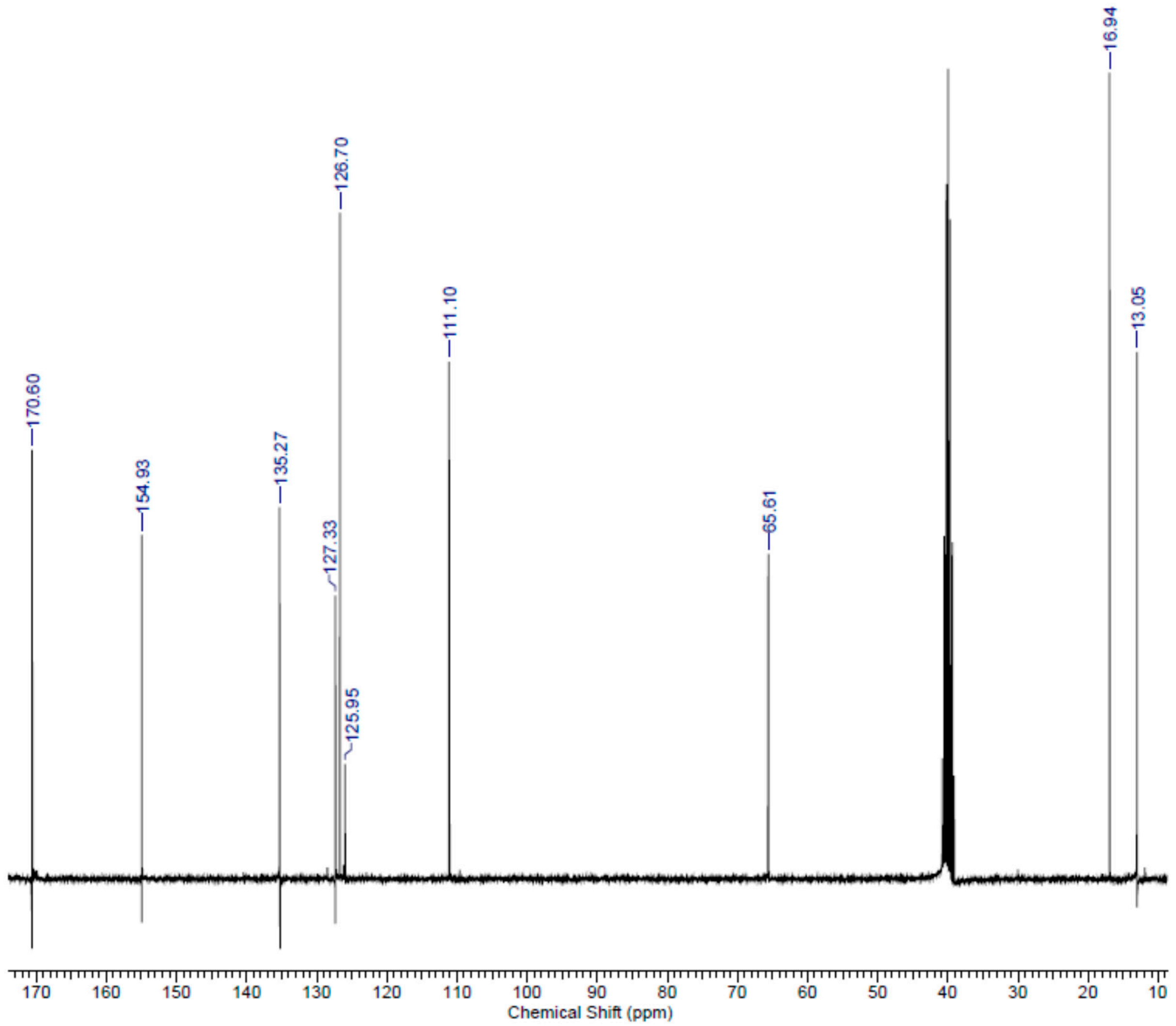

170.60
154.93
135.27
127.33
126.70
125.95
111.10
65.61
16.94
13.05
170
160
150
140
130
120
110
100
90
80
70
60
50
40
30
20
10
Chemical Shift (ppm)

(4-chloro-2,5-dimethylphenoxy)acetic acid **8**

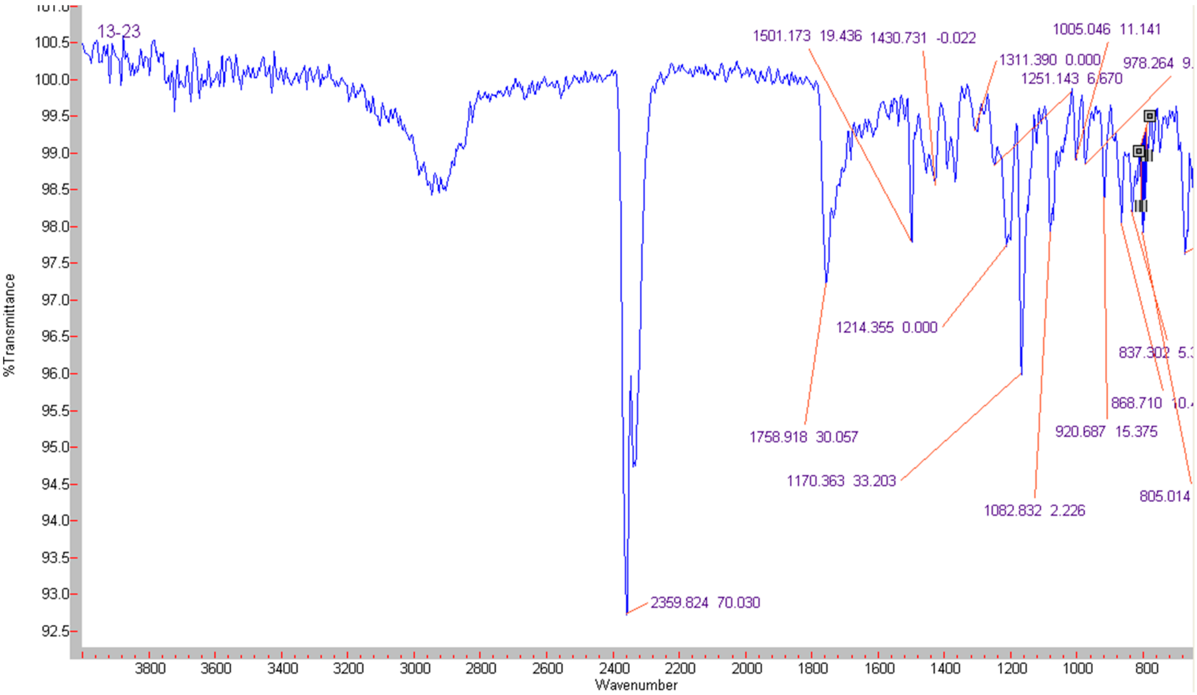

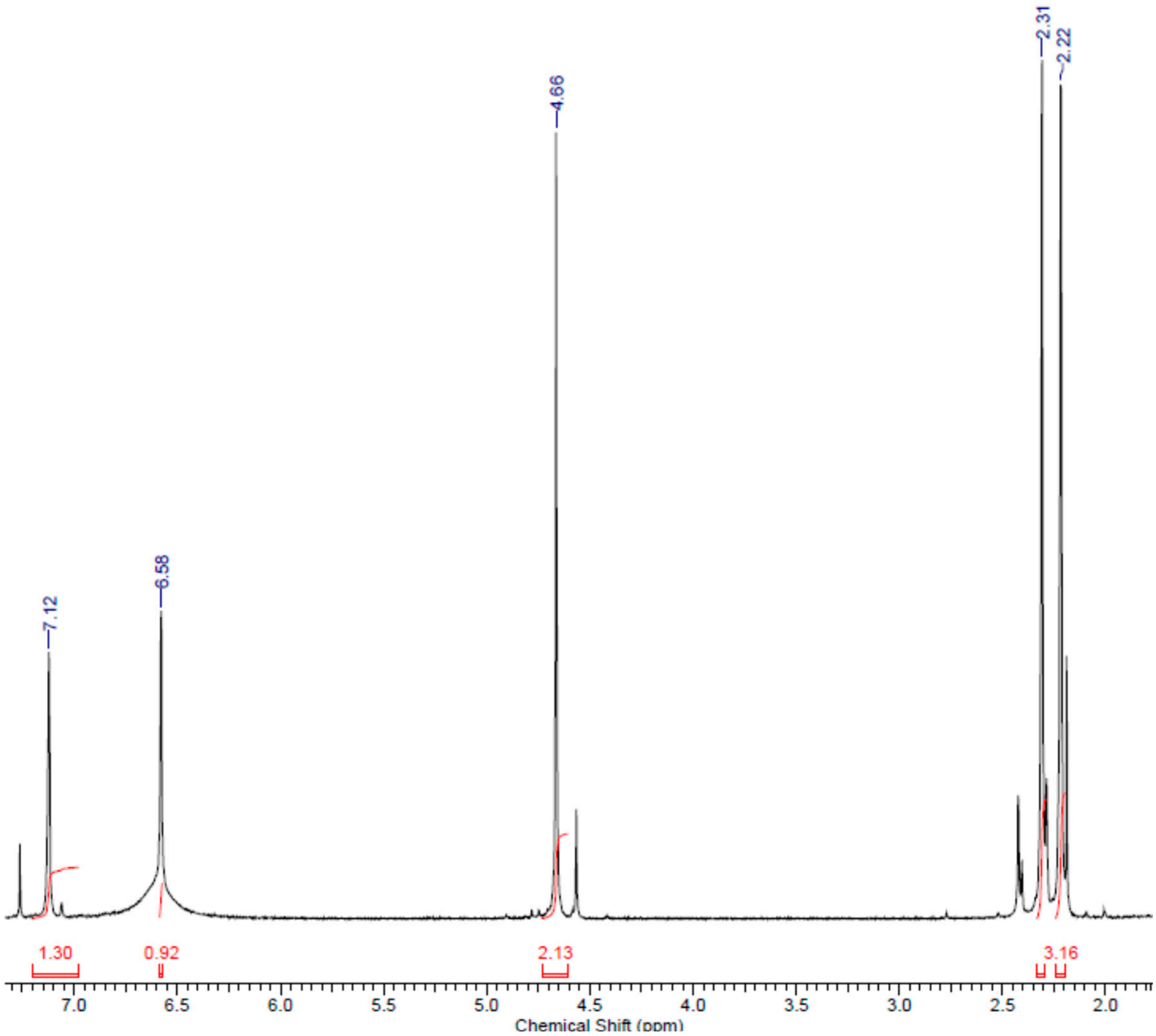

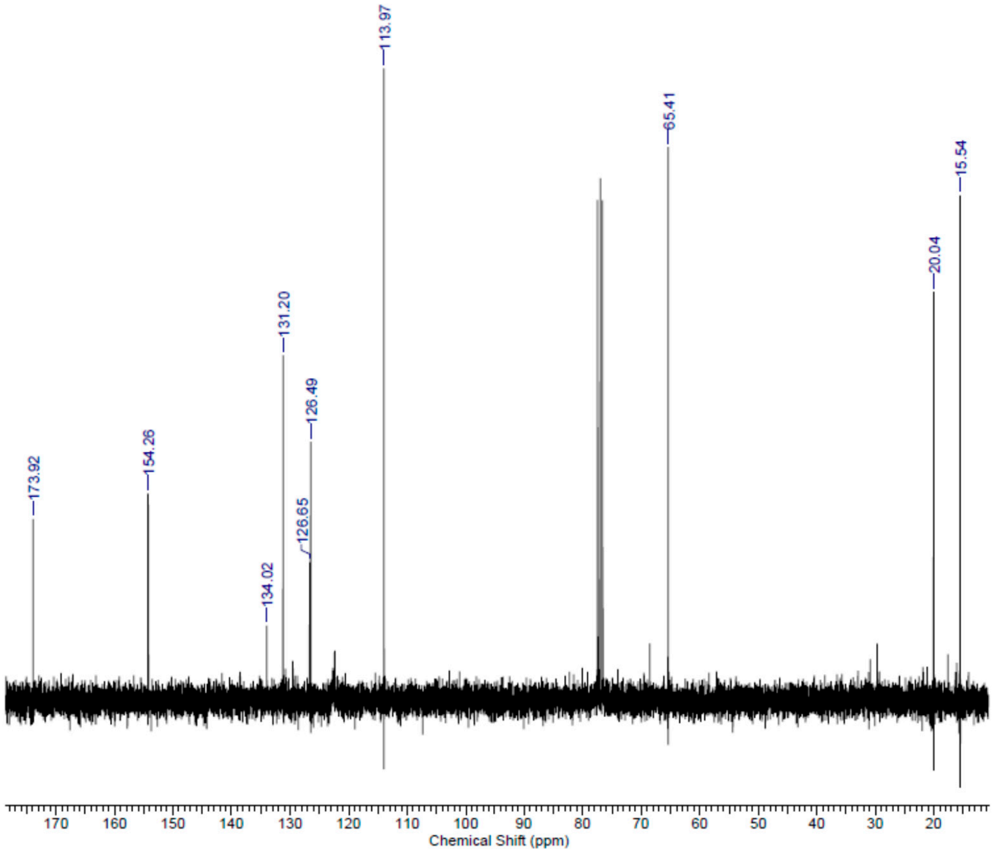

(4-chloro-3,5-dimethylphenoxy)acetic acid **9**

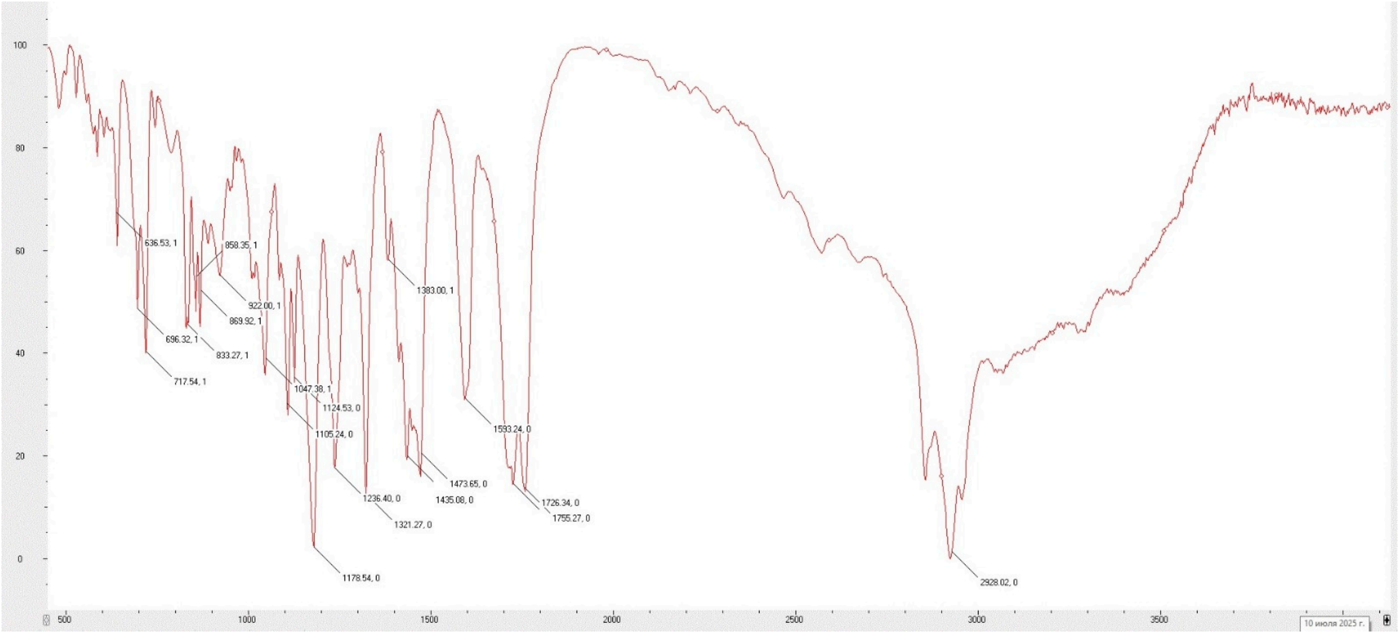

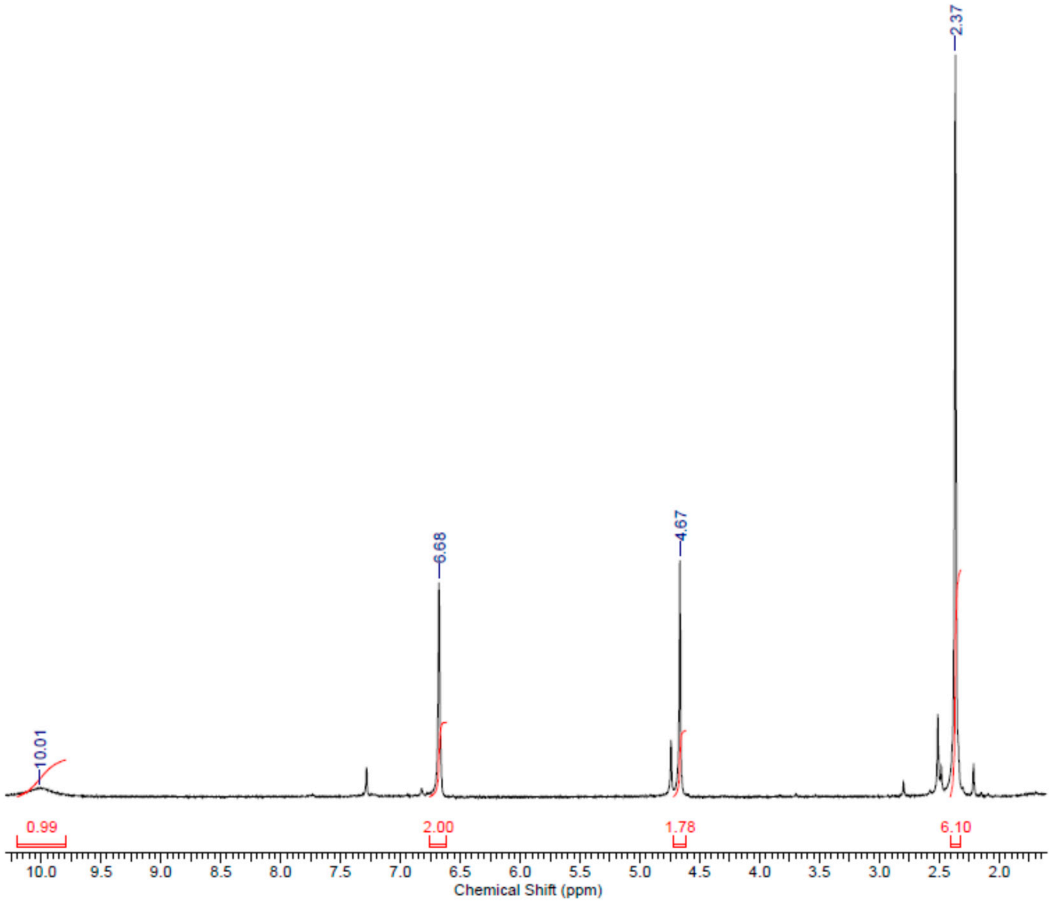
10.01
6.68
4.67
2.37
0.99
2.00
1.78
6.10
10.0 9.5 9.0 8.5 8.0 7.5 7.0 6.5 6.0 5.5 5.0 4.5 4.0 3.5 3.0 2.5 2.0
Chemical Shift (ppm)

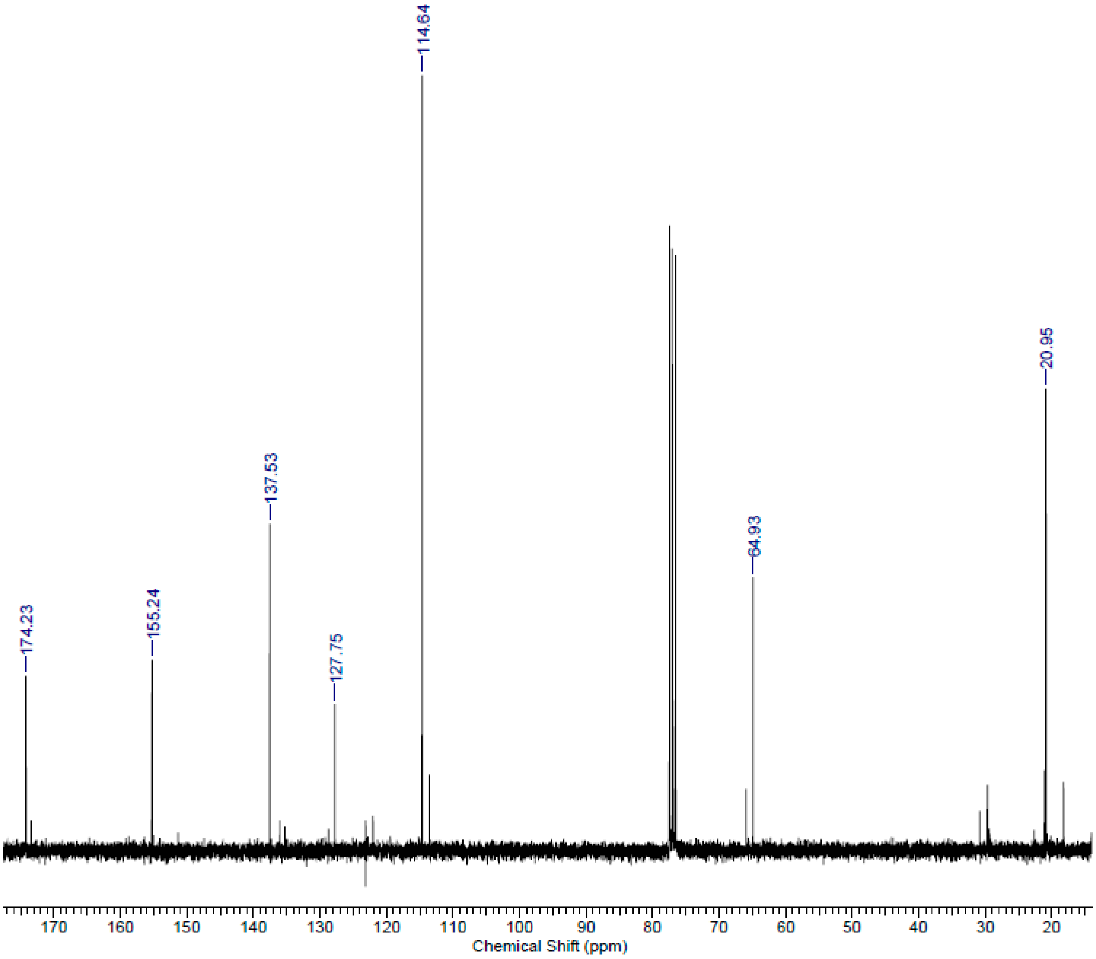
174.23
155.24
137.53
127.75
114.64
64.93
20.95
170 160 150 140 130 120 110 100 90 80 70 60 50 40 30 20
Chemical Shift (ppm)

(4-chloro-2,3,5-trimethylphenoxy)acetic acid **10**

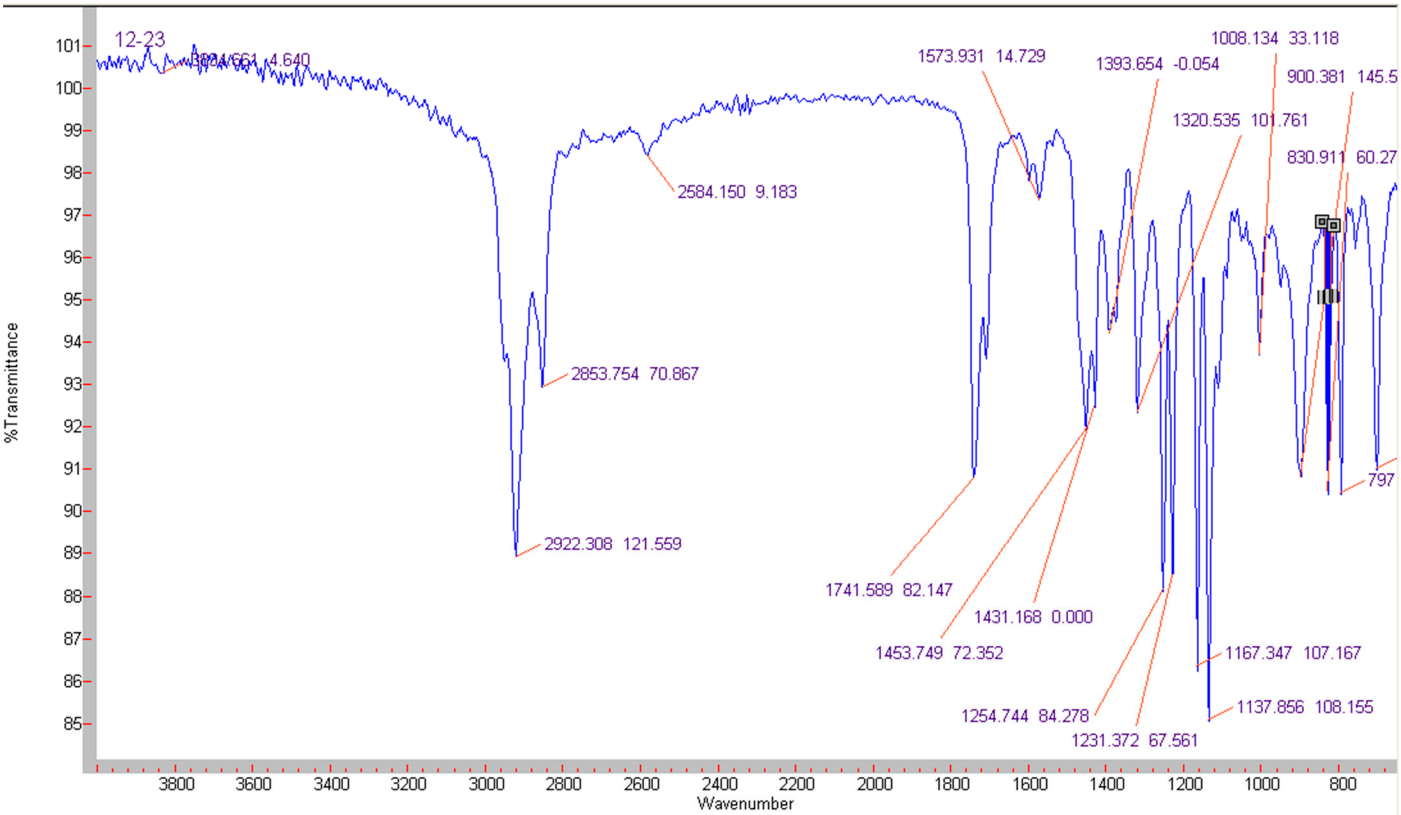

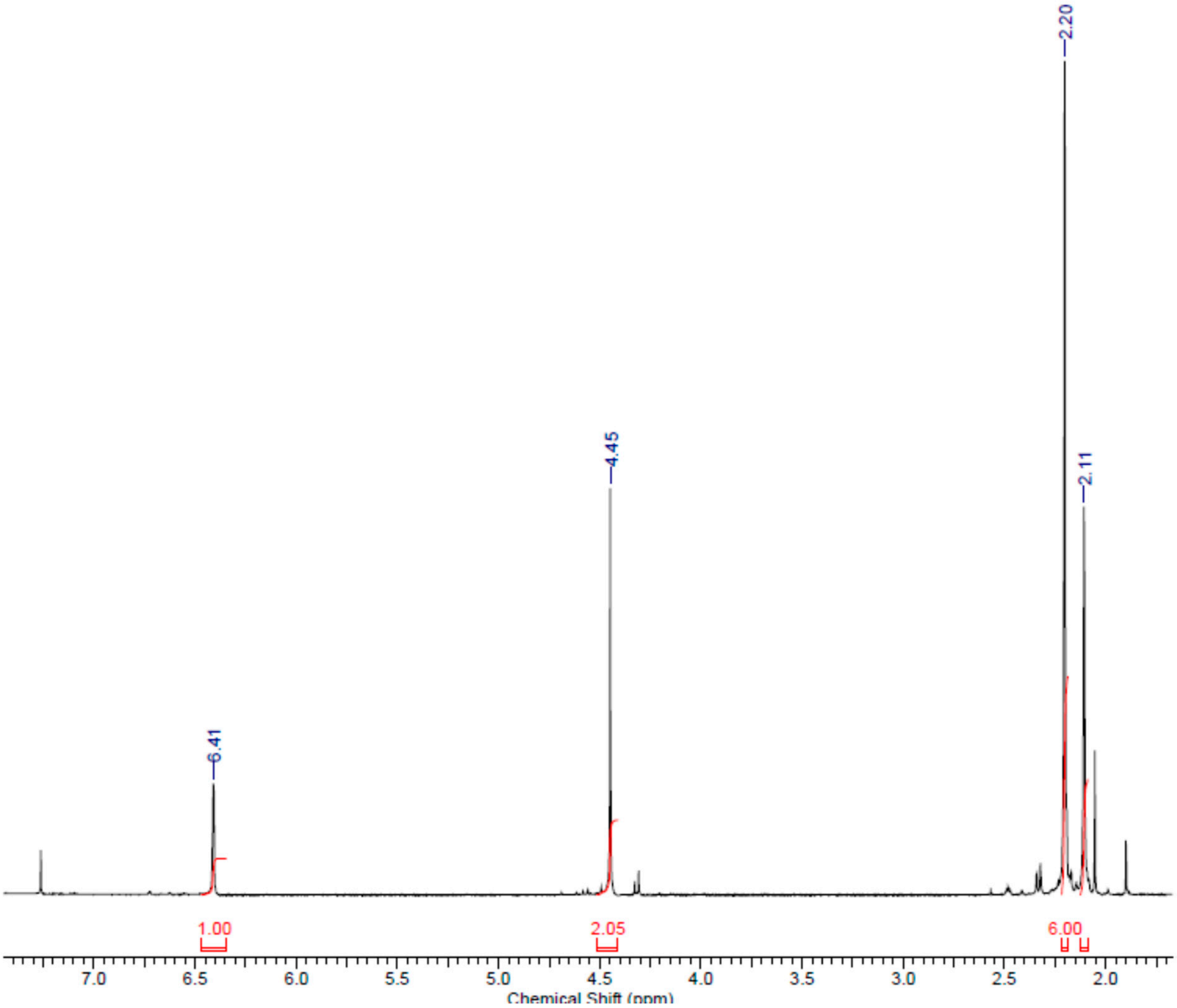

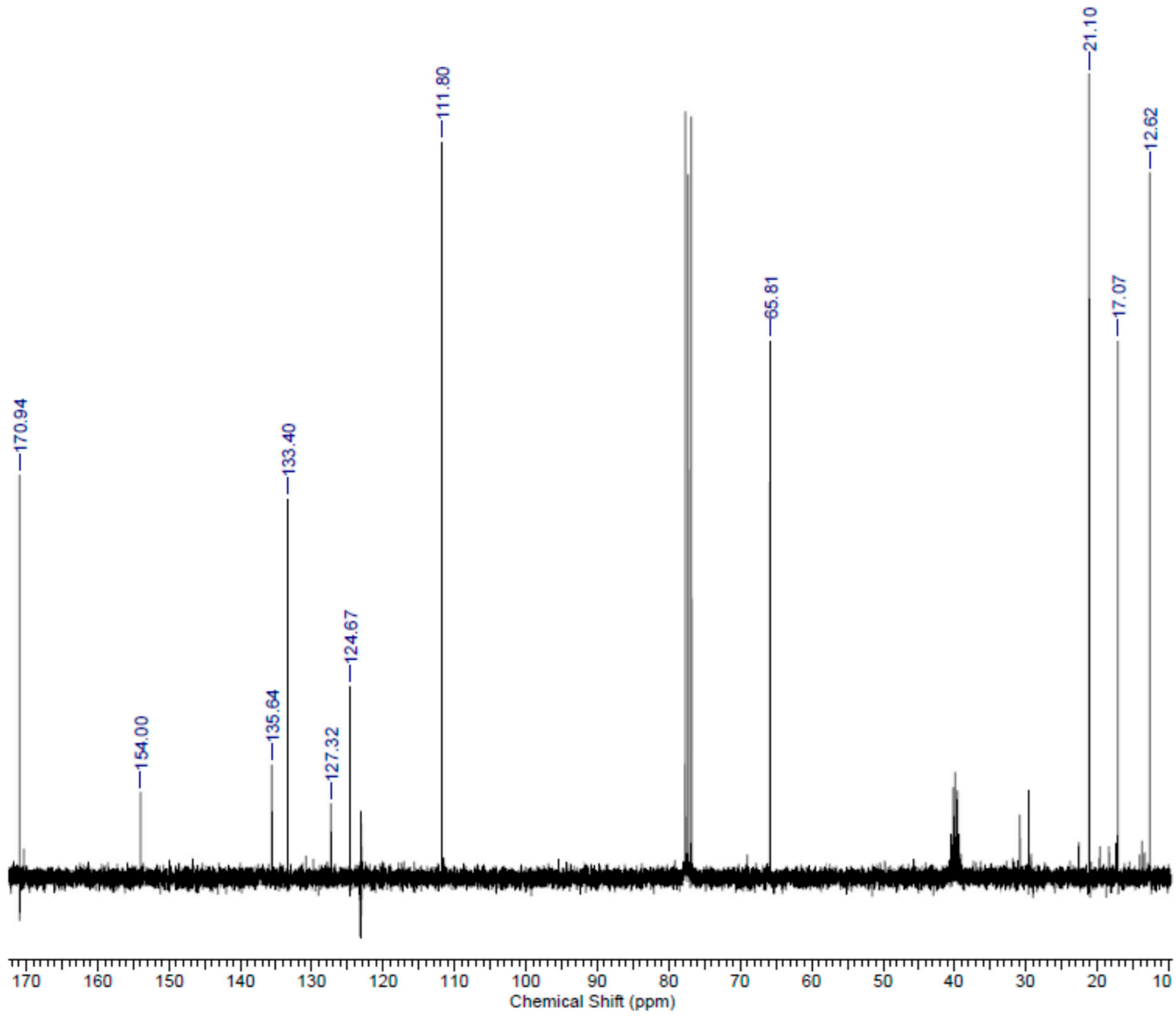

(4-bromo-2-isopropyl-5-methylphenoxy)acetic acid **11**

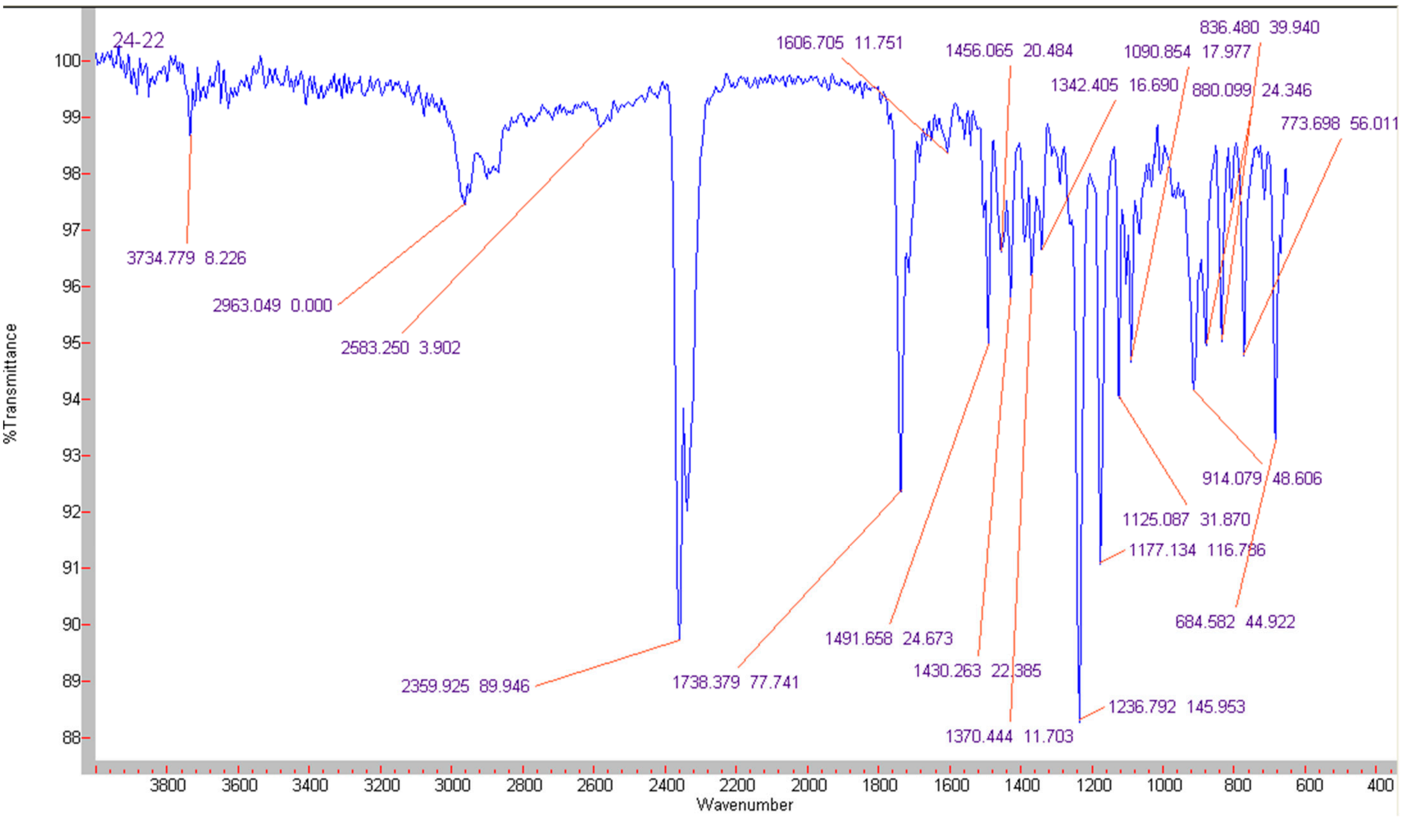

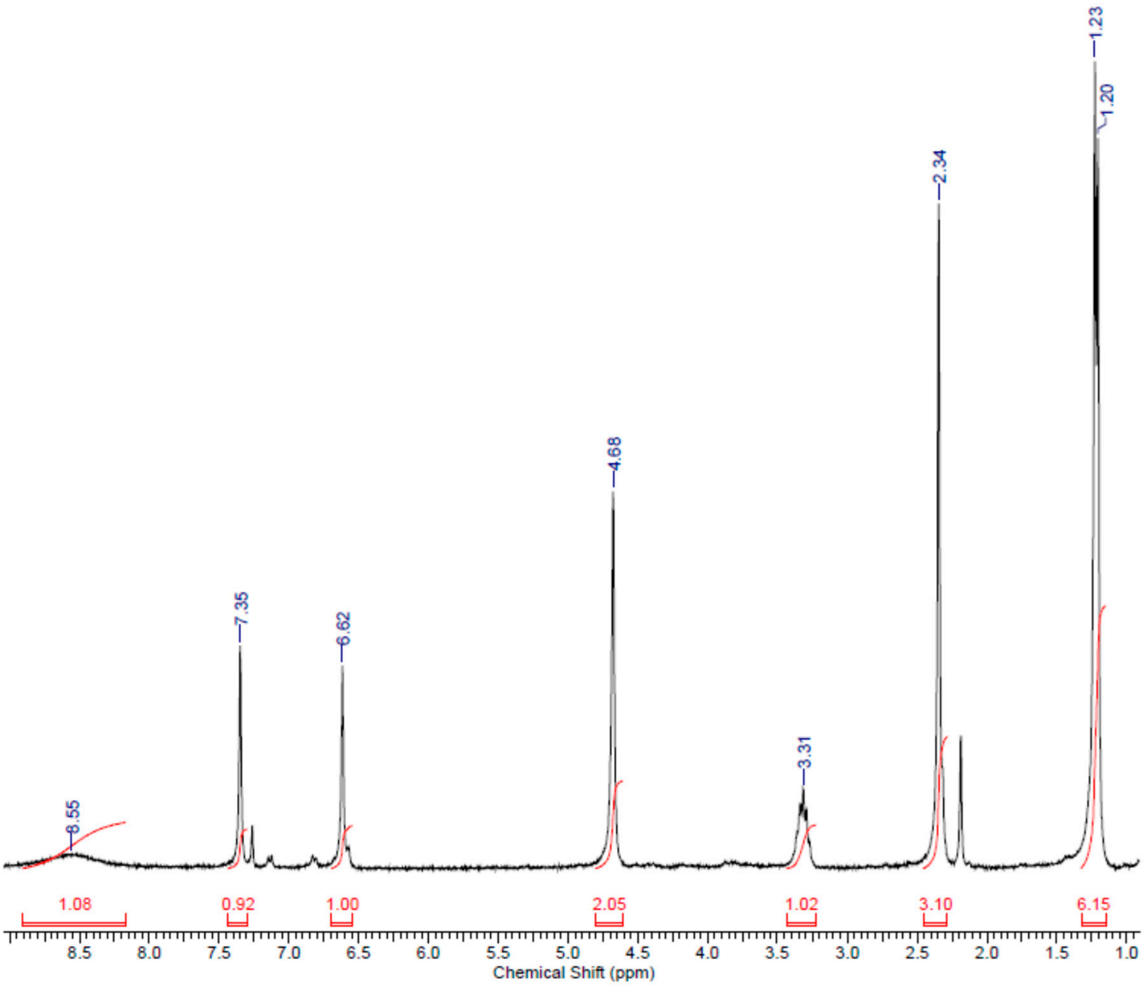
8.55
7.35
6.62
4.68
3.31
2.34
1.23
1.20
1.08
0.92
1.00
2.05
1.02
3.10
6.15
8.5
8.0
7.5
7.0
6.5
6.0
5.5
5.0
4.5
4.0
3.5
3.0
2.5
2.0
1.5
1.0
Chemical Shift (ppm)

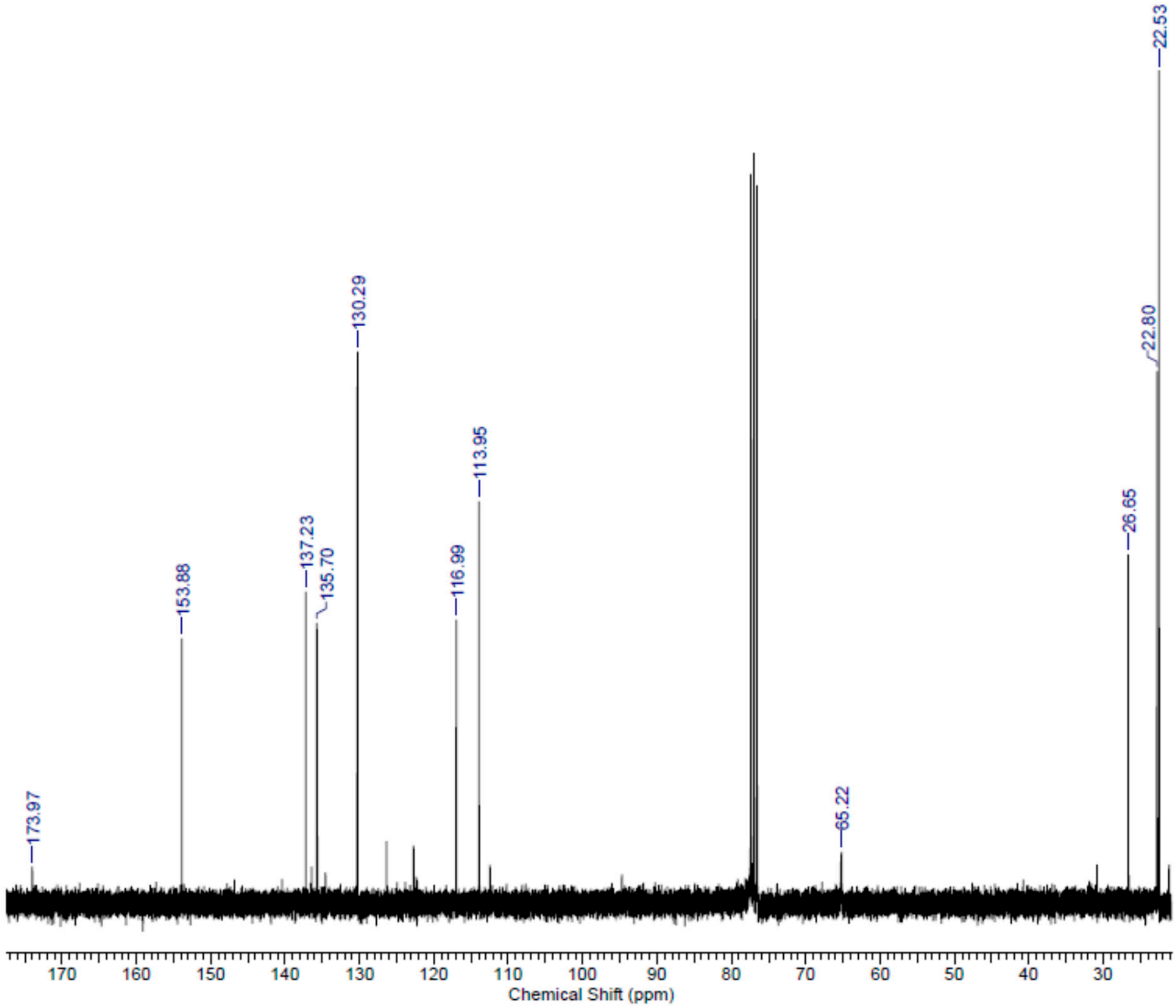
173.97
153.88
137.23
135.70
130.29
116.99
113.95
65.22
26.65
22.80
22.53
170
160
150
140
130
120
110
100
90
80
70
60
50
40
30
Chemical Shift (ppm)

(4- bromo -2,3-dimethylphenoxy)acetic acid **12**

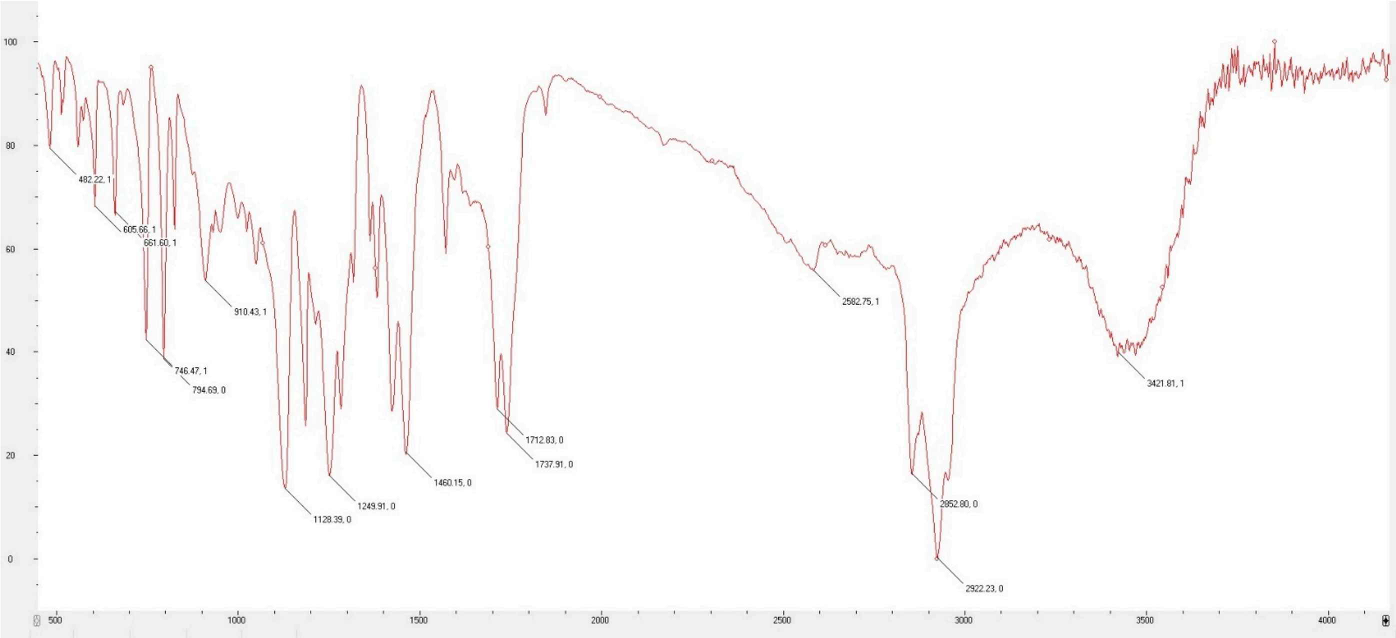

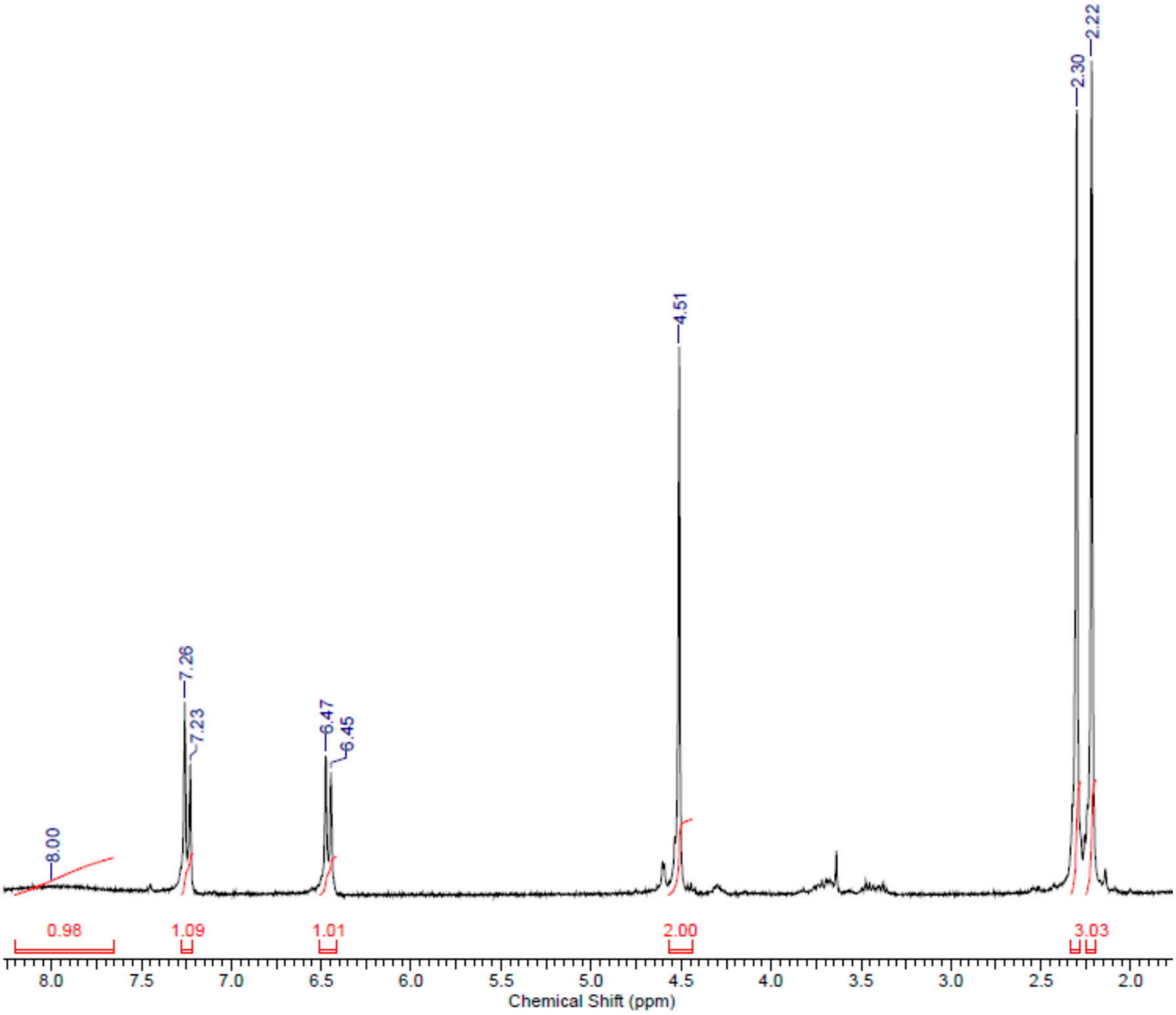

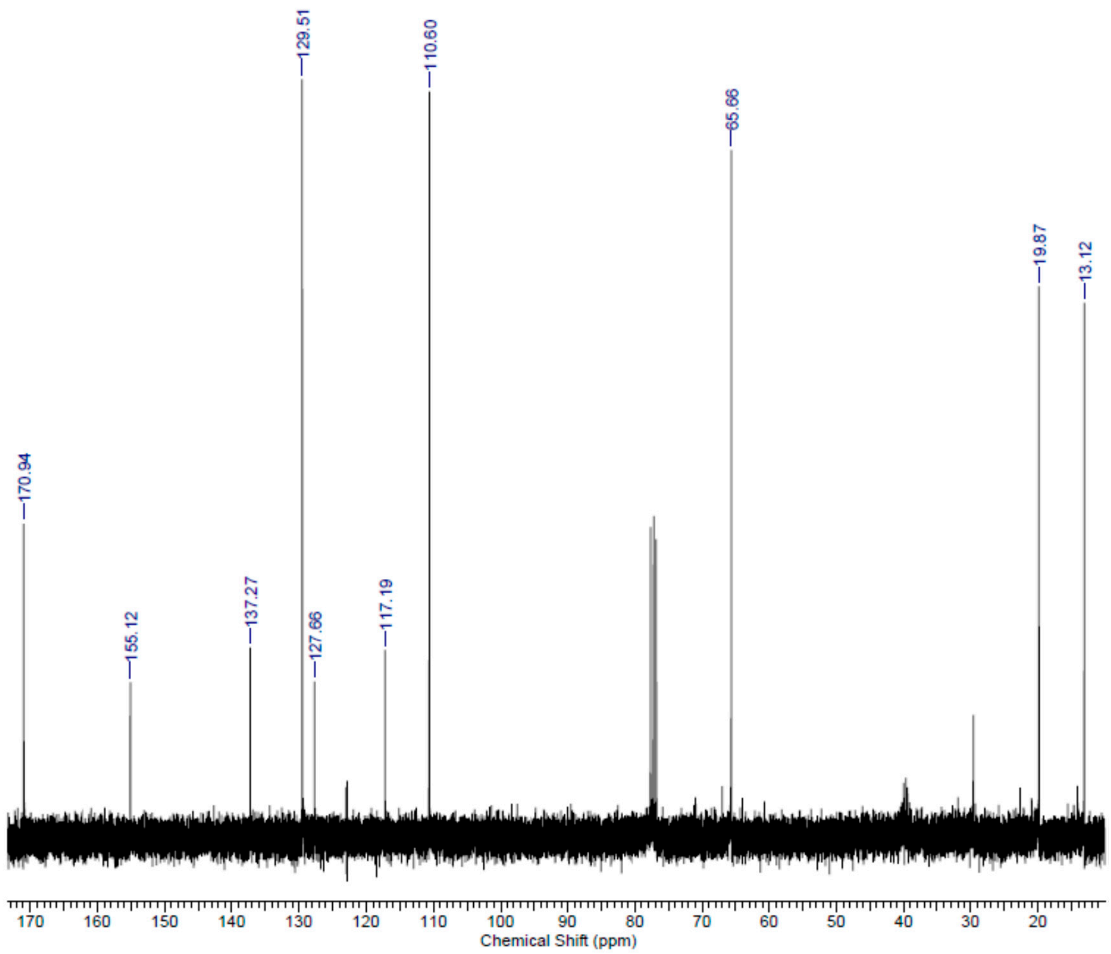

(4- bromo -2,5-dimethylphenoxy)acetic acid **13**

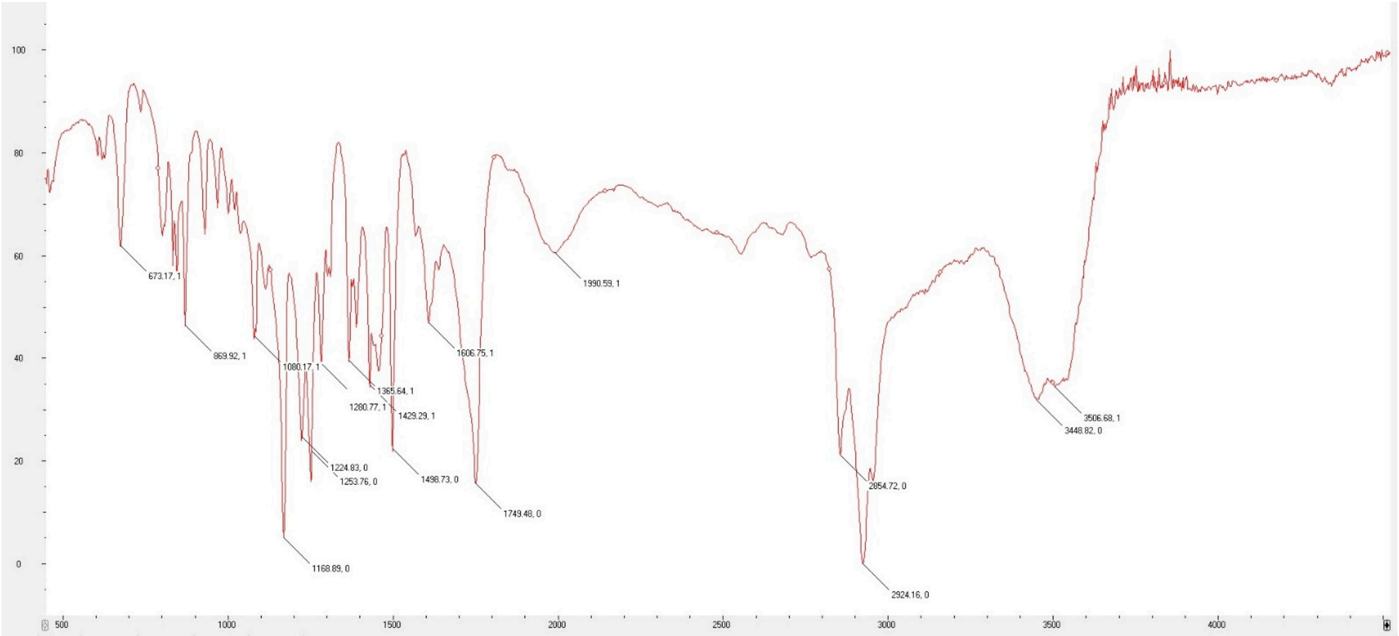

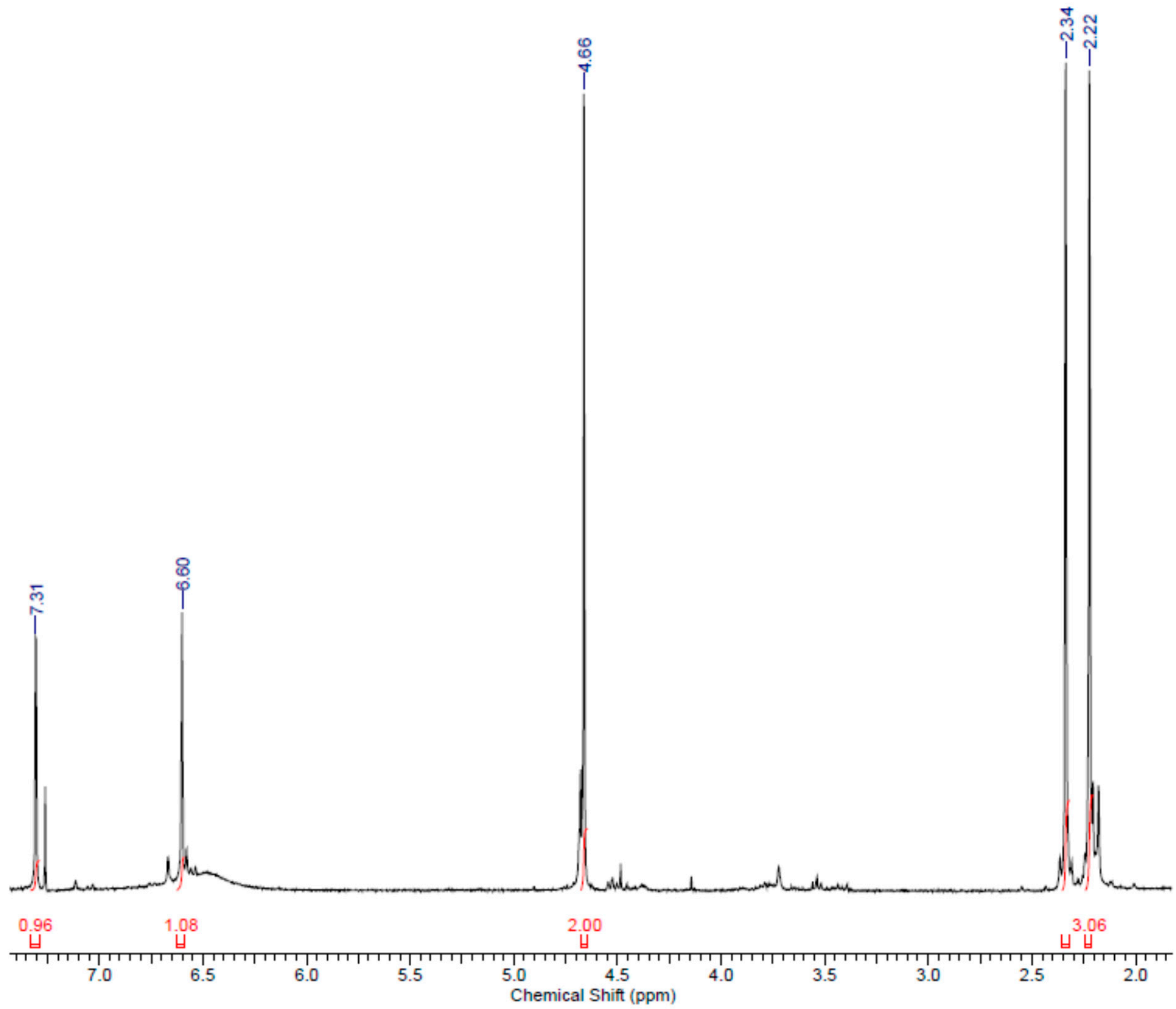

7.31
6.60
4.66
2.34
2.22
0.96
1.08
2.00
3.06
7.0
6.5
6.0
5.5
5.0
4.5
4.0
3.5
3.0
2.5
2.0
Chemical Shift (ppm)

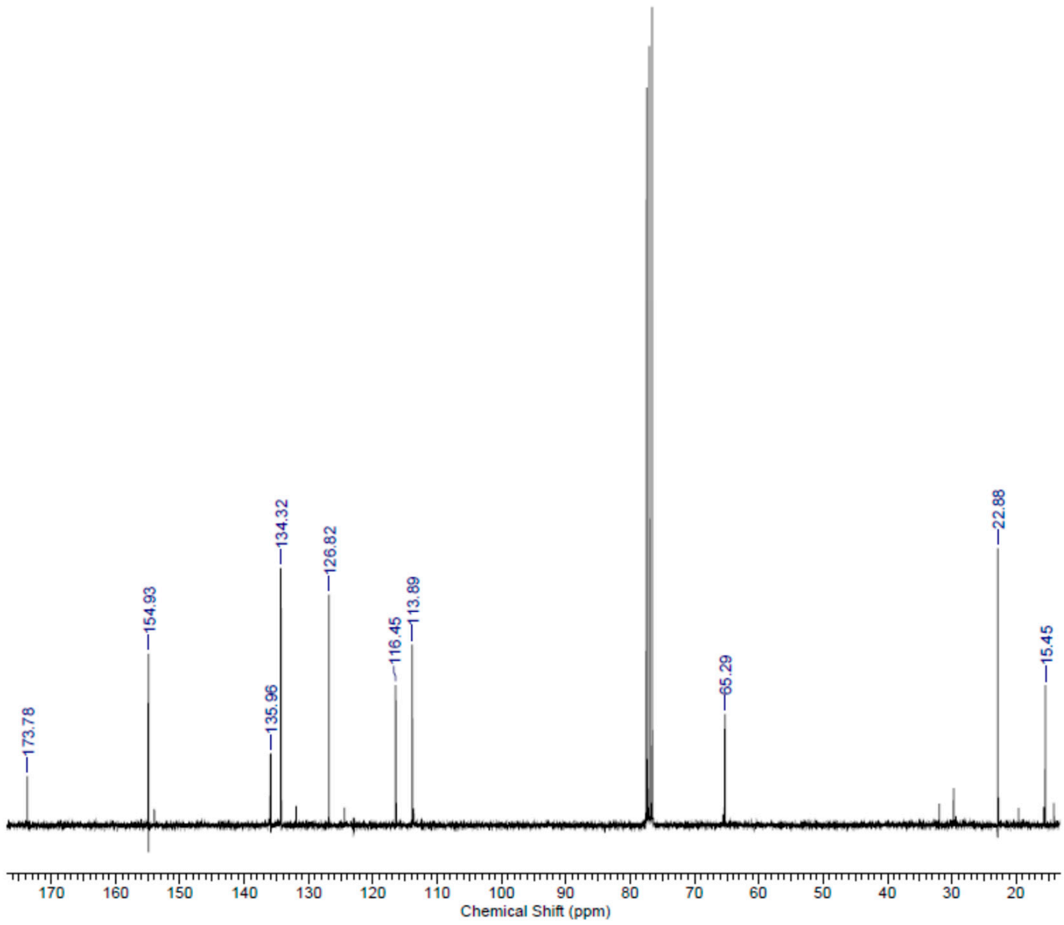

173.78
154.93
135.96
134.32
126.82
116.45
113.89
65.29
22.88
15.45
170
160
150
140
130
120
110
100
90
80
70
60
50
40
30
20
Chemical Shift (ppm)

(4- bromo -3,5-dimethylphenoxy)acetic acid **14**

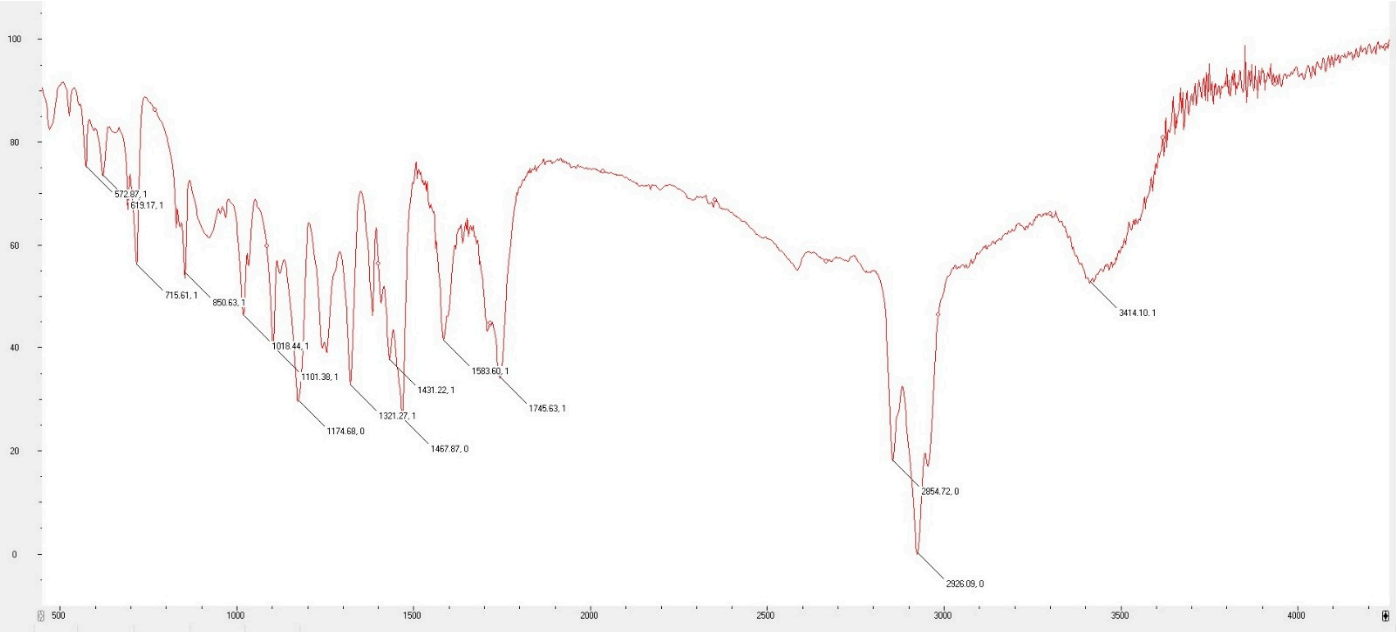

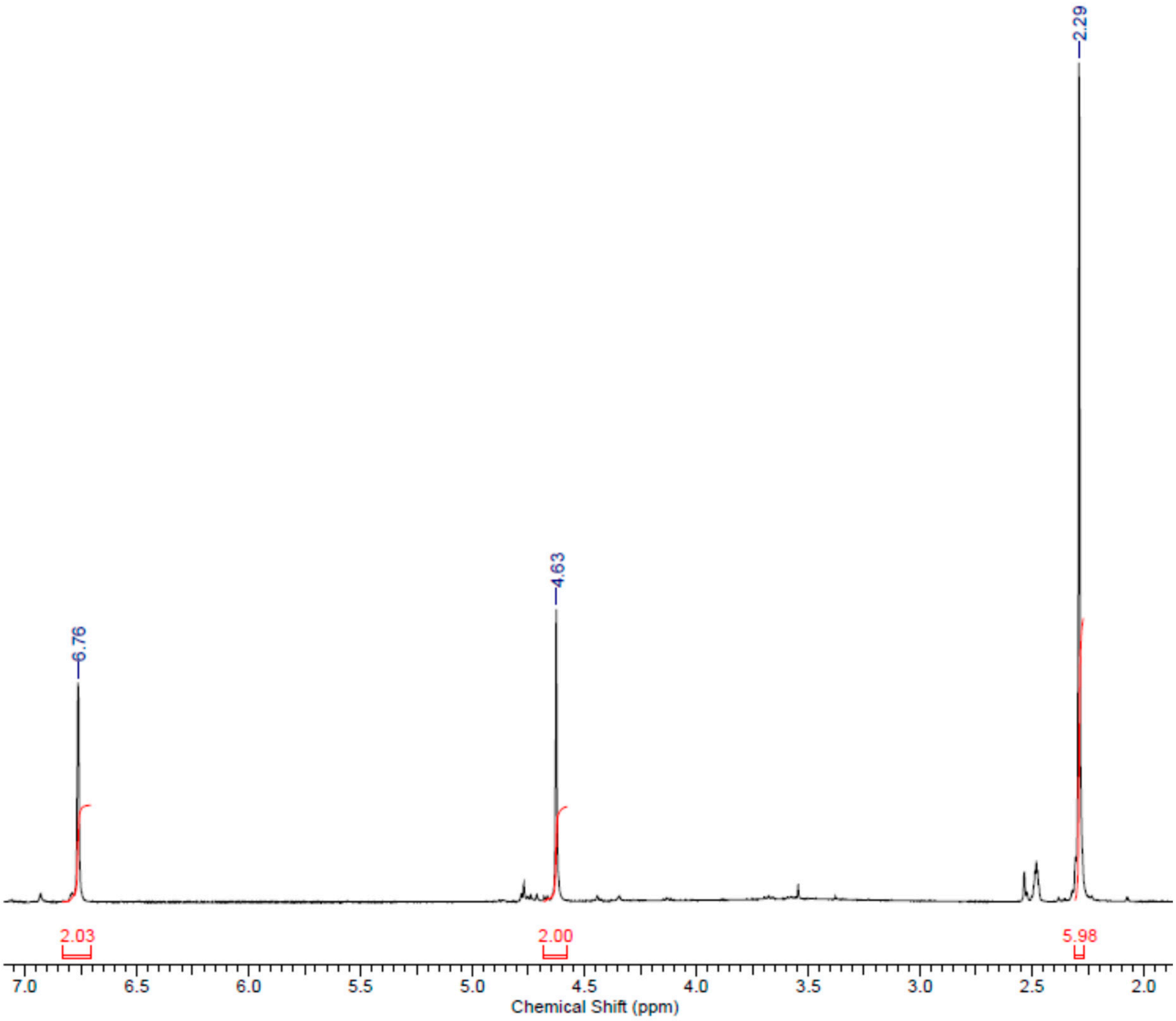

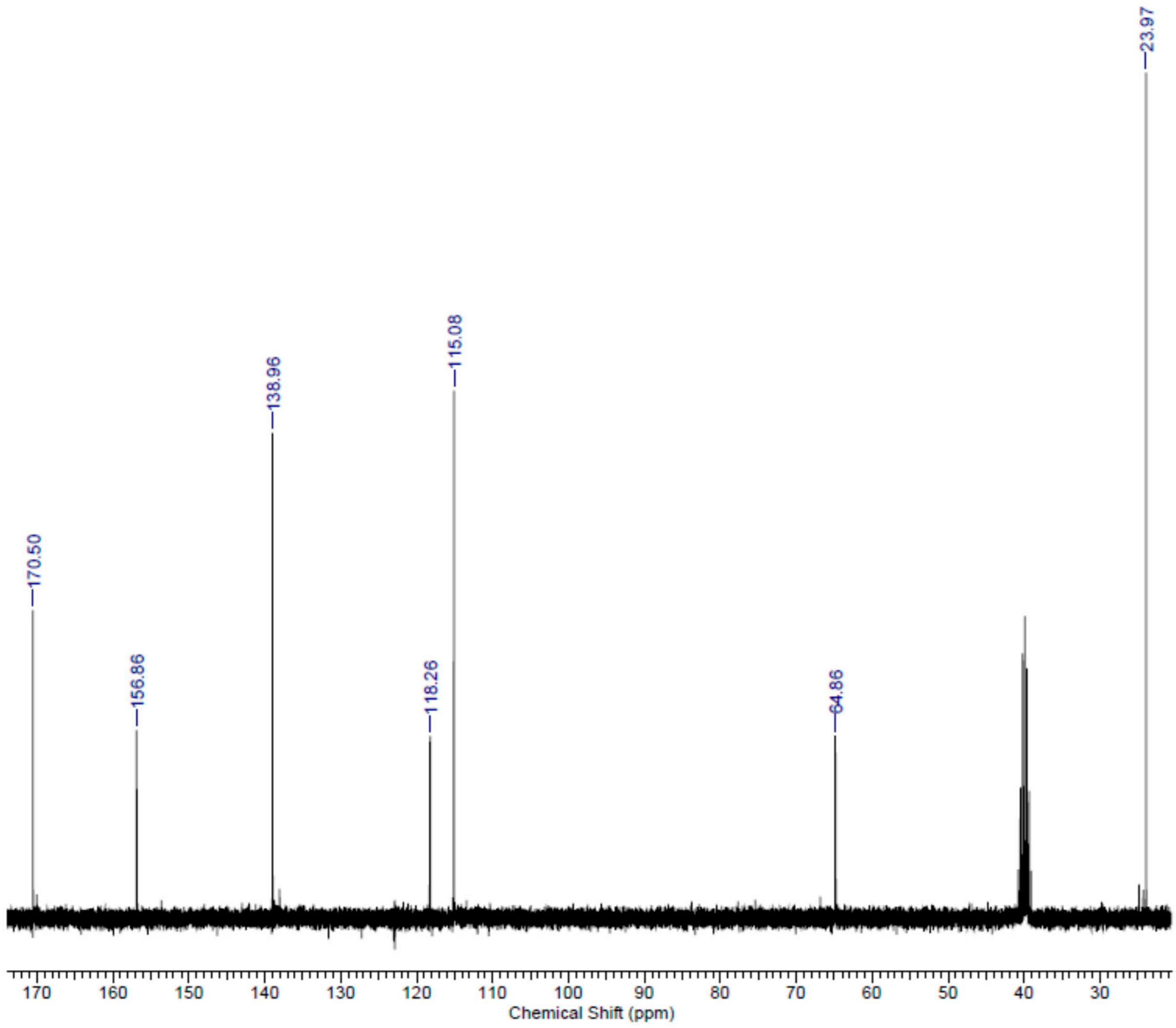

(4- bromo -2,3,5-trimethylphenoxy)acetic acid **15**

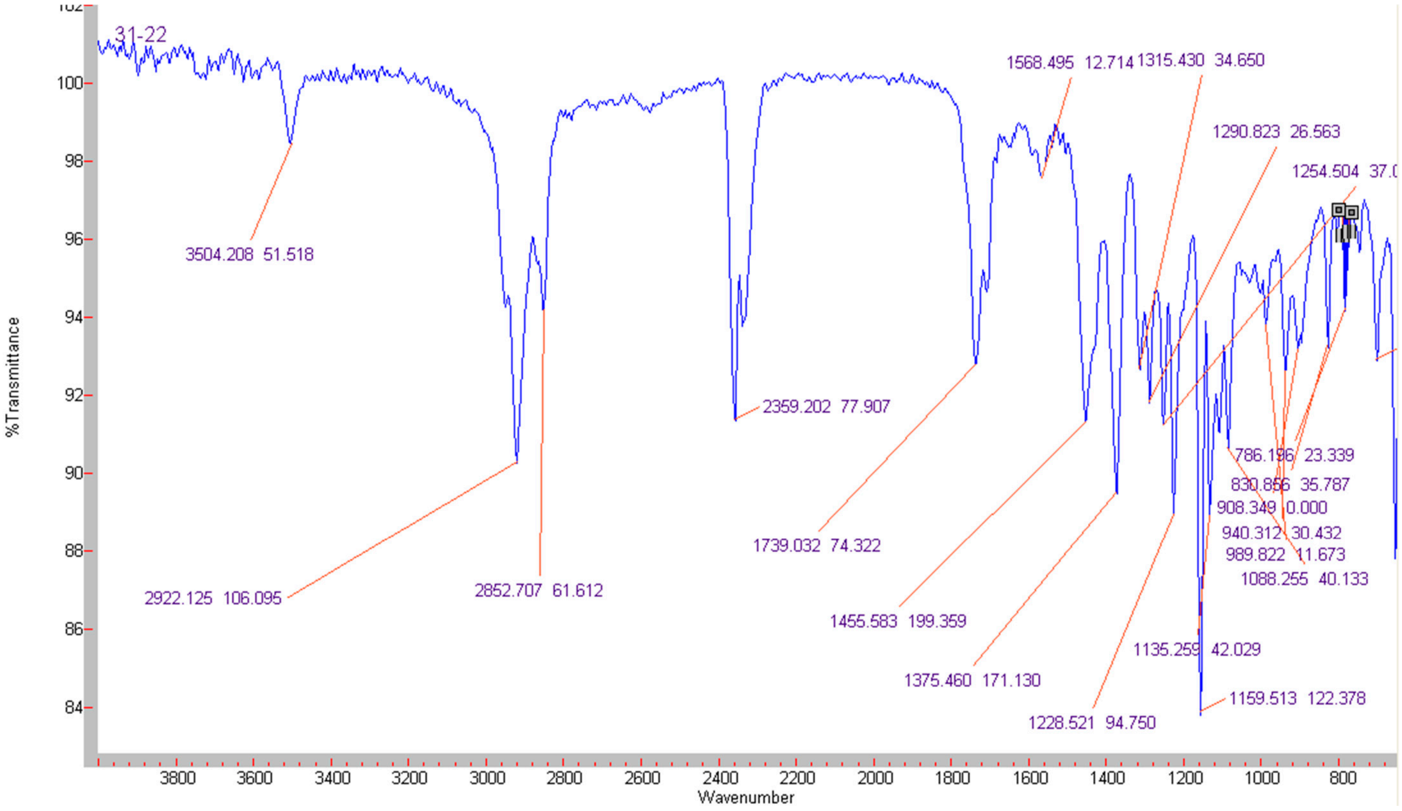

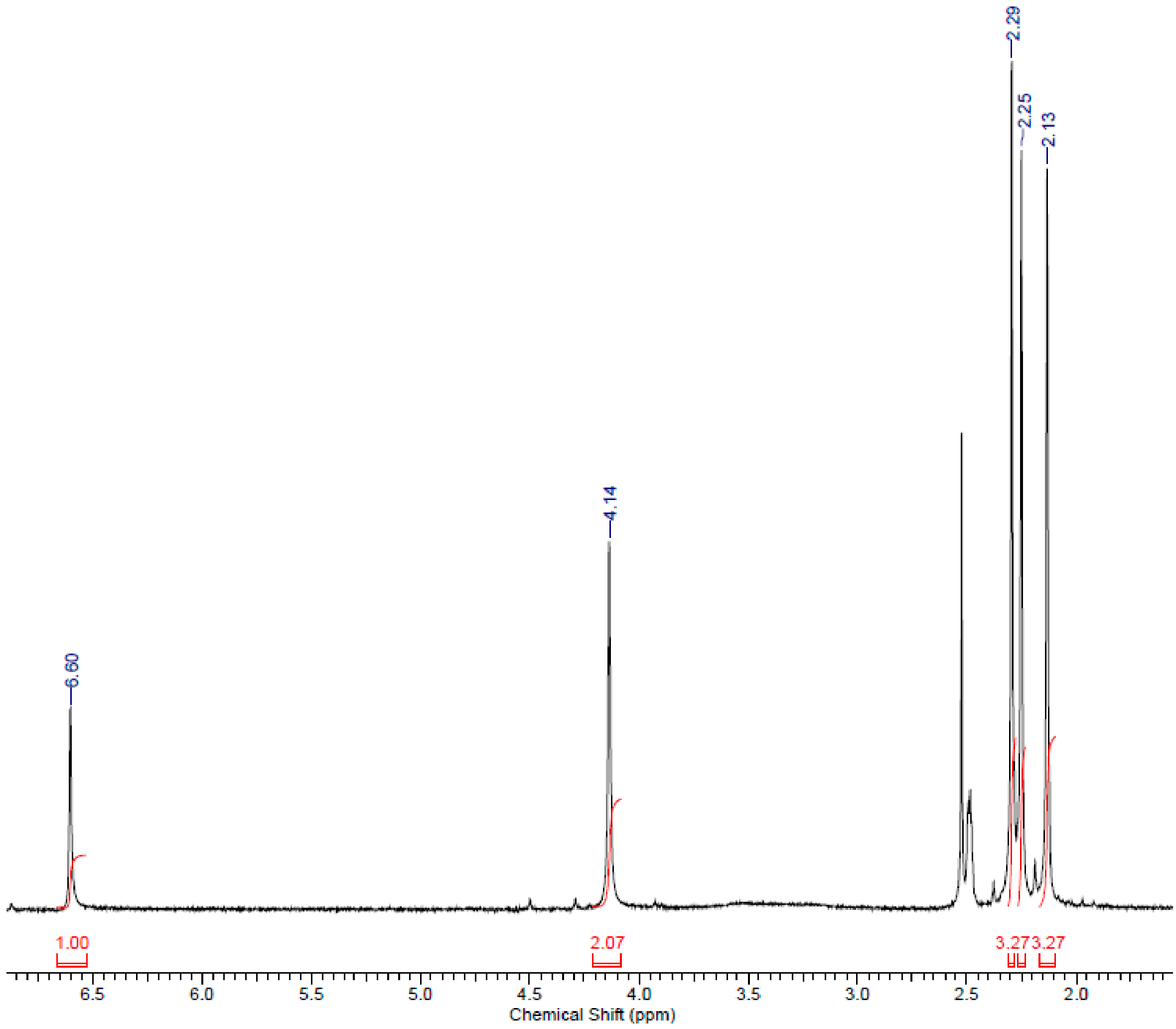

6.60
4.14
2.29
2.25
2.13
1.00
2.07
3.27
3.27
6.5
6.0
5.5
5.0
4.5
4.0
3.5
3.0
2.5
2.0
Chemical Shift (ppm)

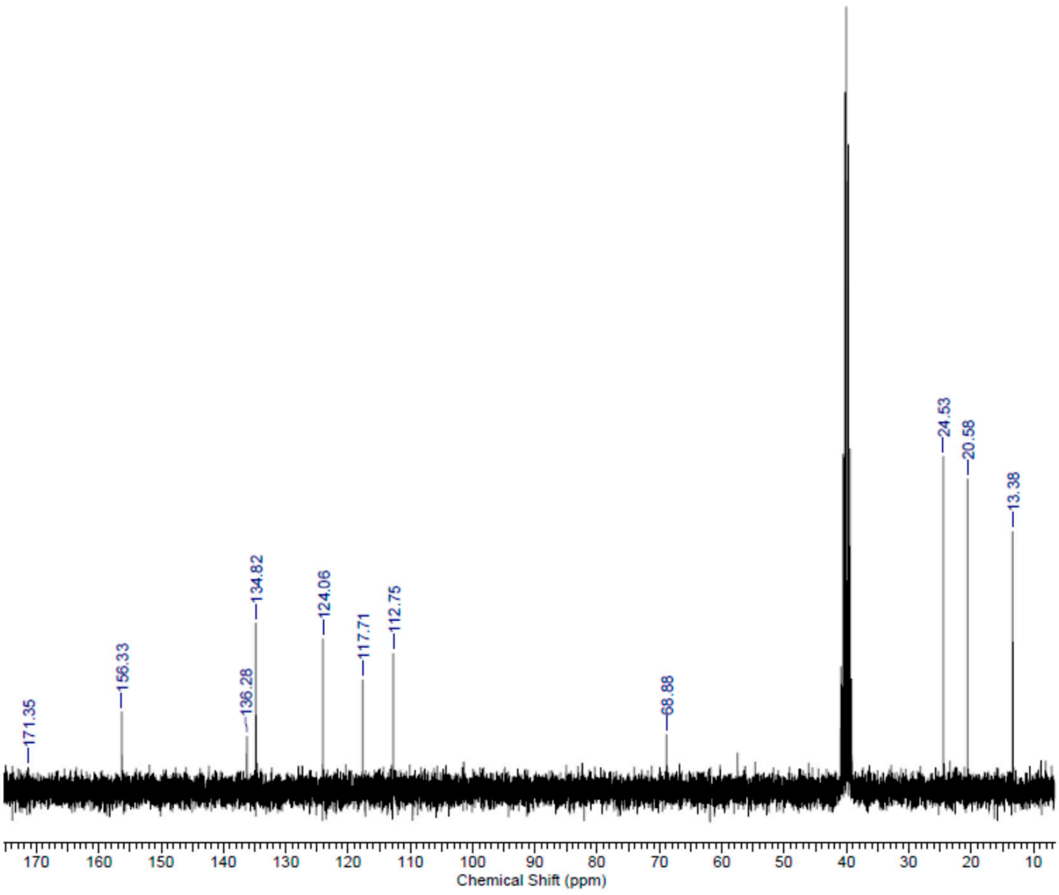

171.35
156.33
136.28
134.82
124.06
117.71
112.75
68.88
24.53
20.58
13.38
170
160
150
140
130
120
110
100
90
80
70
60
50
40
30
20
10
Chemical Shift (ppm)

(4-iodo-2-isopropyl-5-methylphenoxy)acetic acid **16**

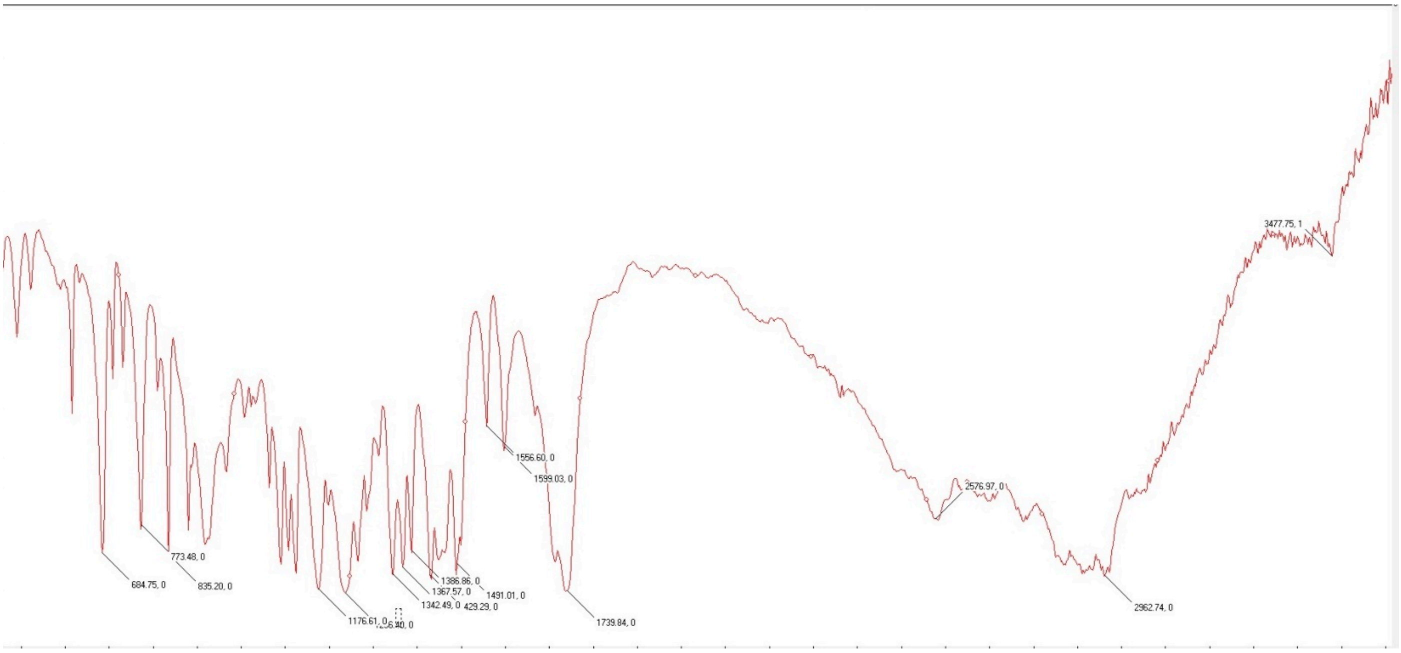

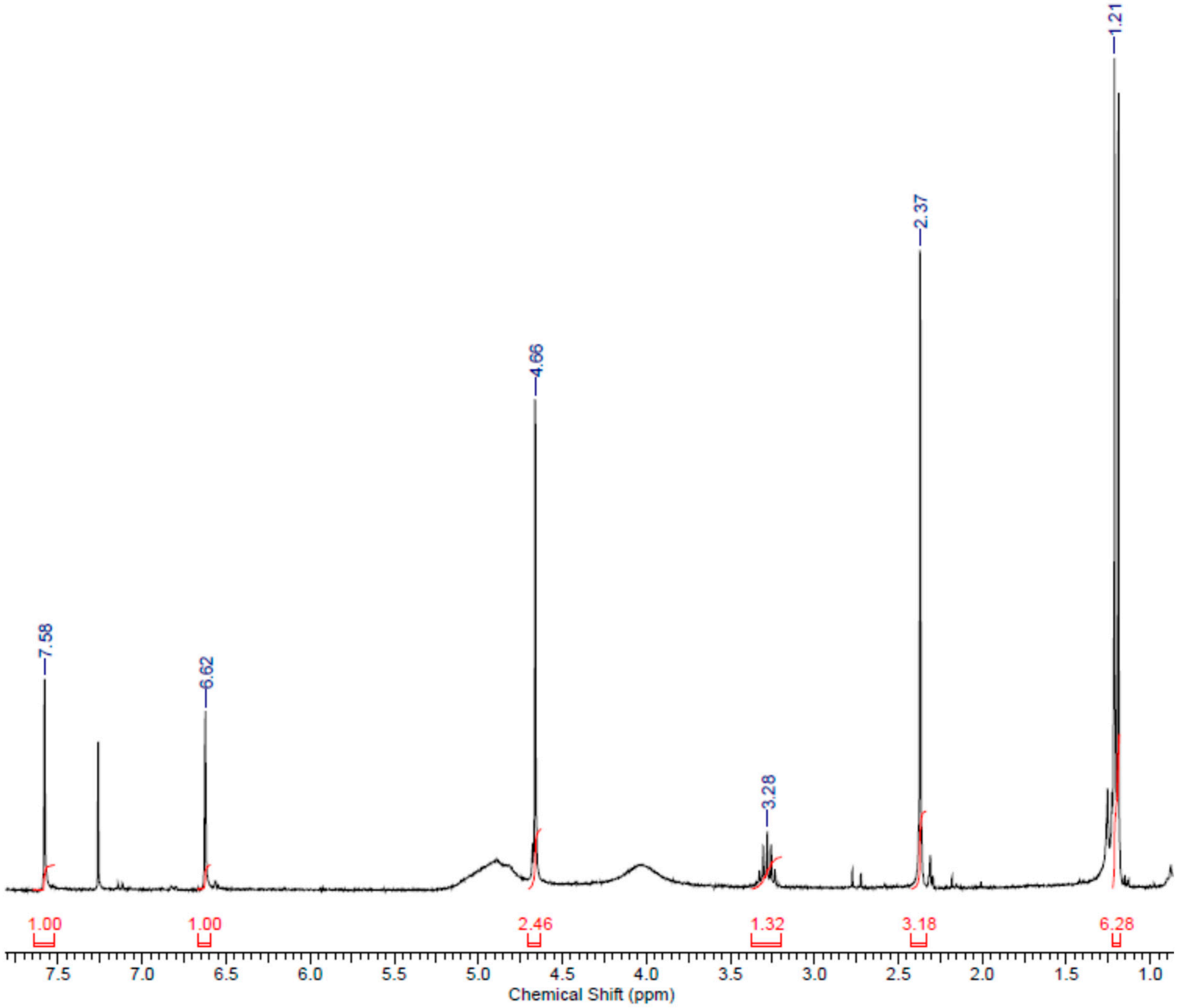

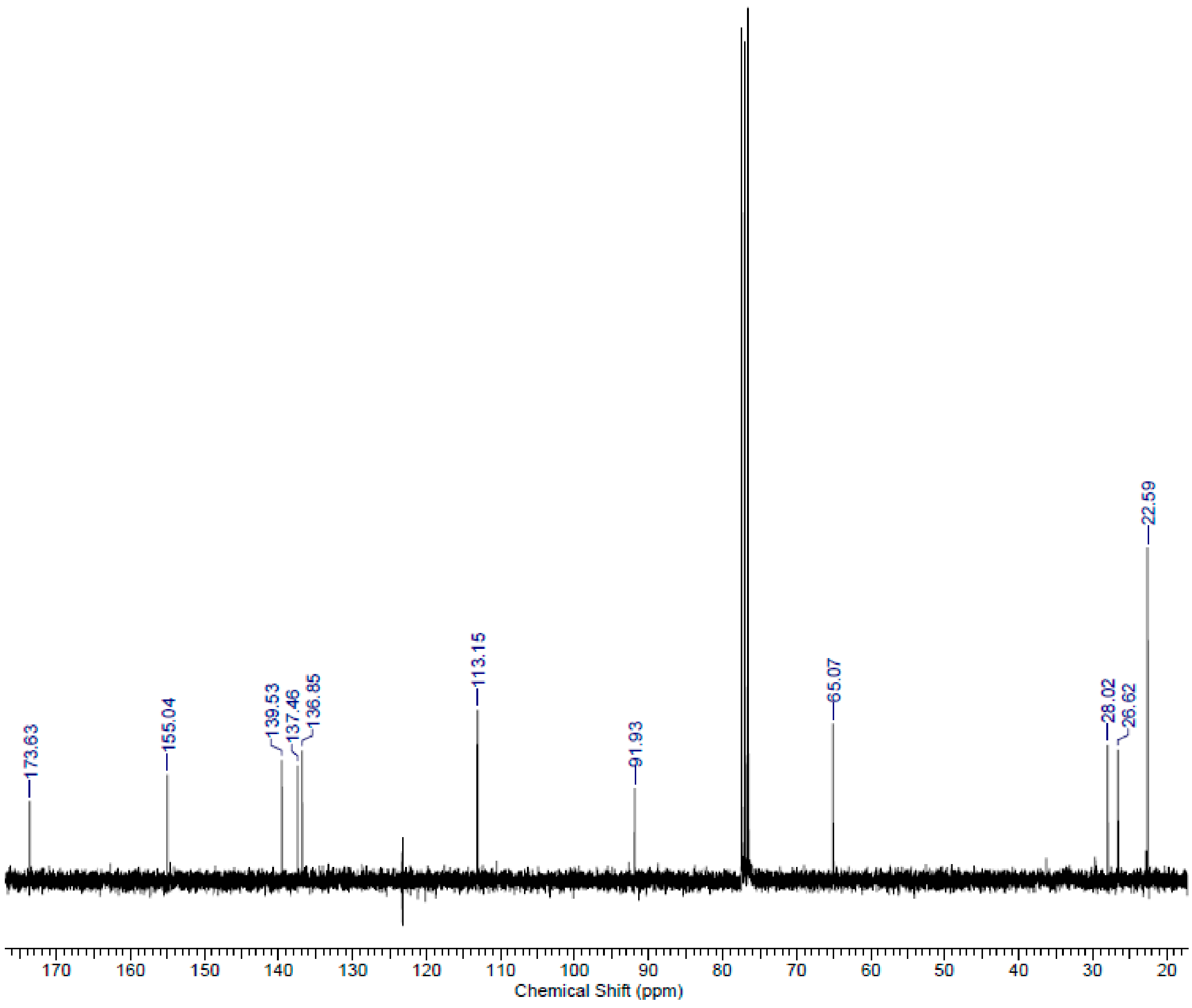

(4- iodo -2,3-dimethylphenoxy)acetic acid **17**

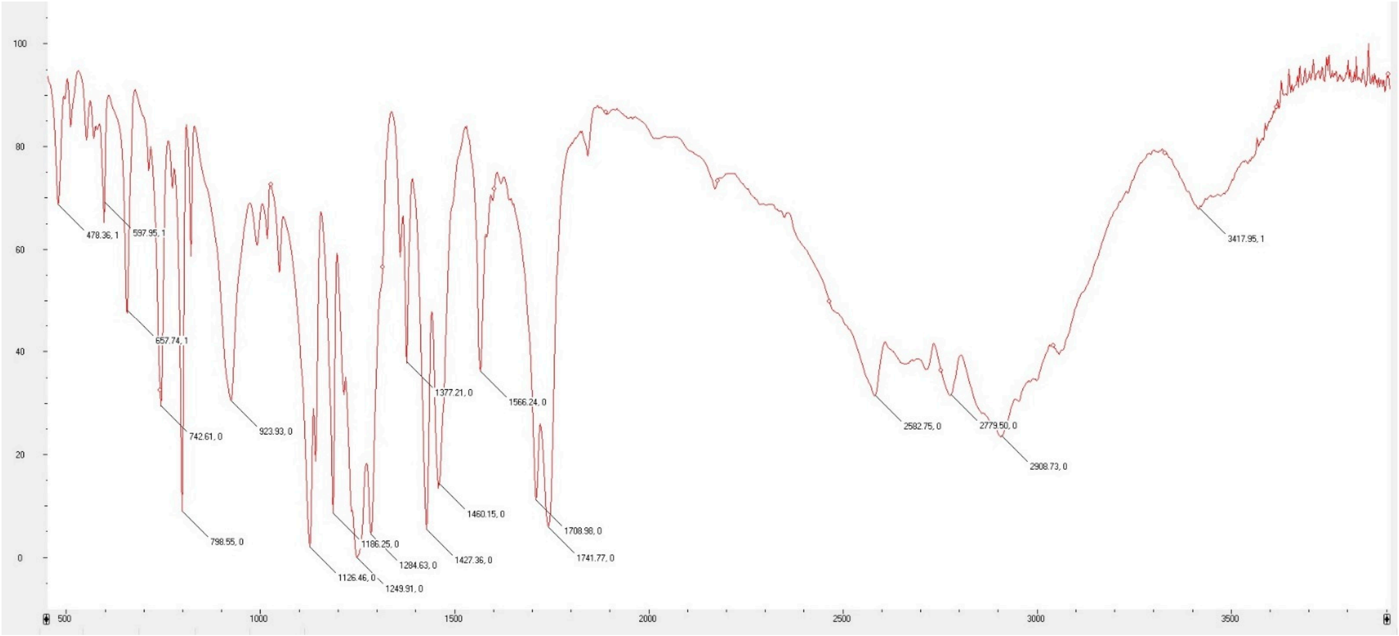

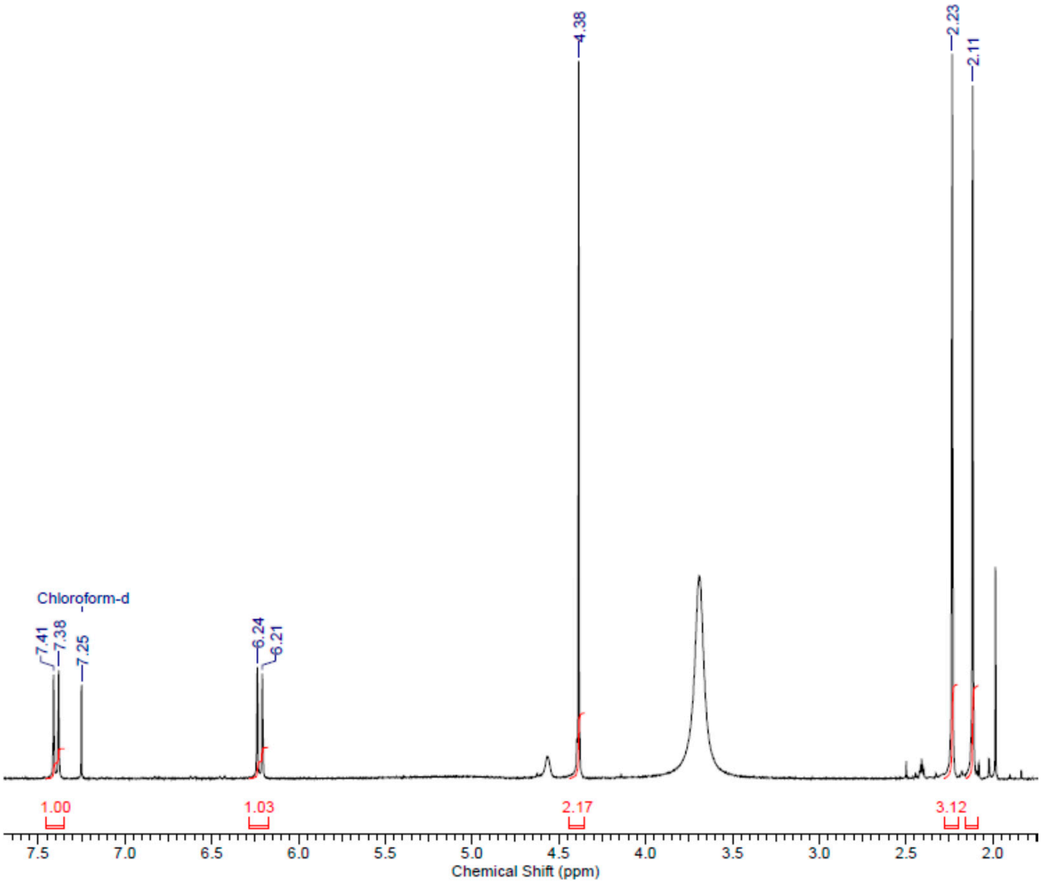

Chloroform-d
7.41
7.38
7.25
6.24
6.21
4.38
2.23
2.11
1.00
1.03
2.17
3.12
7.5
7.0
6.5
6.0
5.5
5.0
4.5
4.0
3.5
3.0
2.5
2.0
Chemical Shift (ppm)

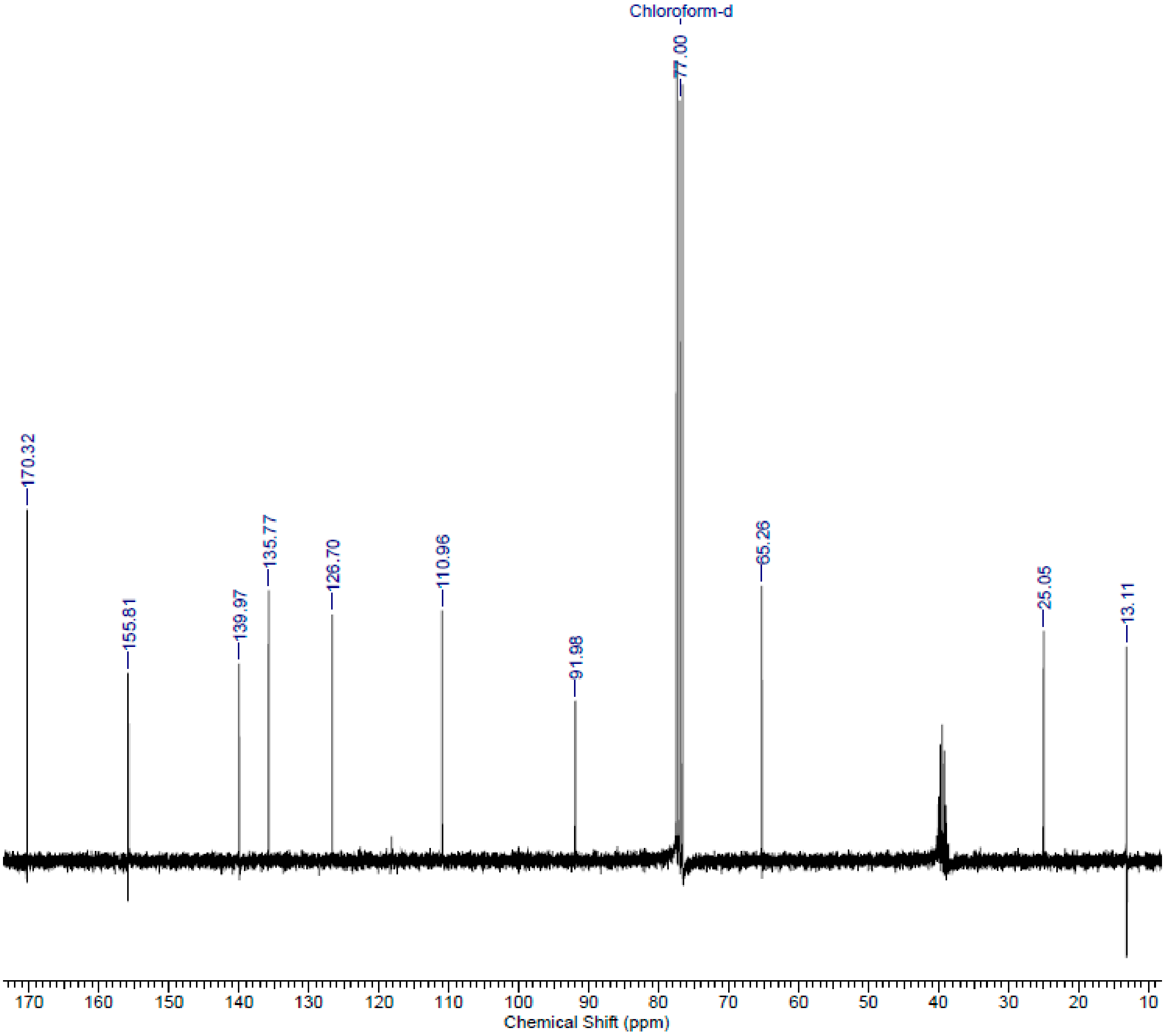

Chloroform-d
77.00
170.32
155.81
139.97
135.77
126.70
110.96
91.98
65.26
25.05
13.11
170
160
150
140
130
120
110
100
90
80
70
60
50
40
30
20
10
Chemical Shift (ppm)

(4- iodo -2,5-dimethylphenoxy)acetic acid **18**

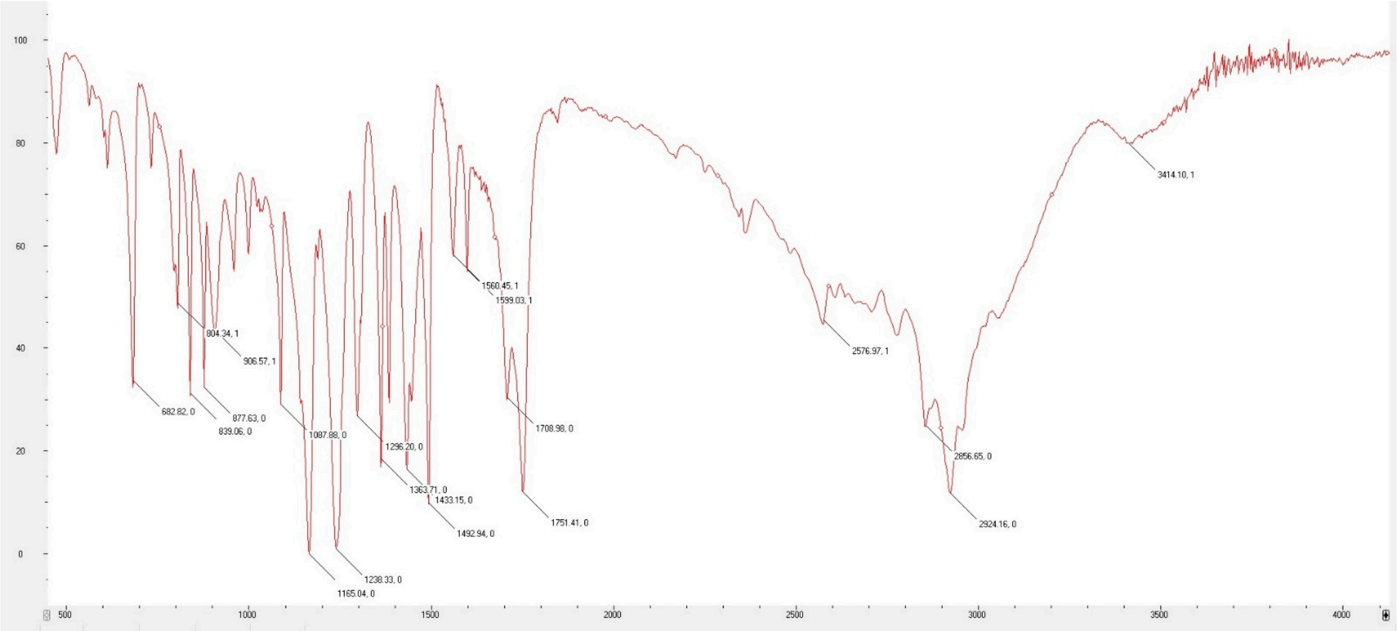

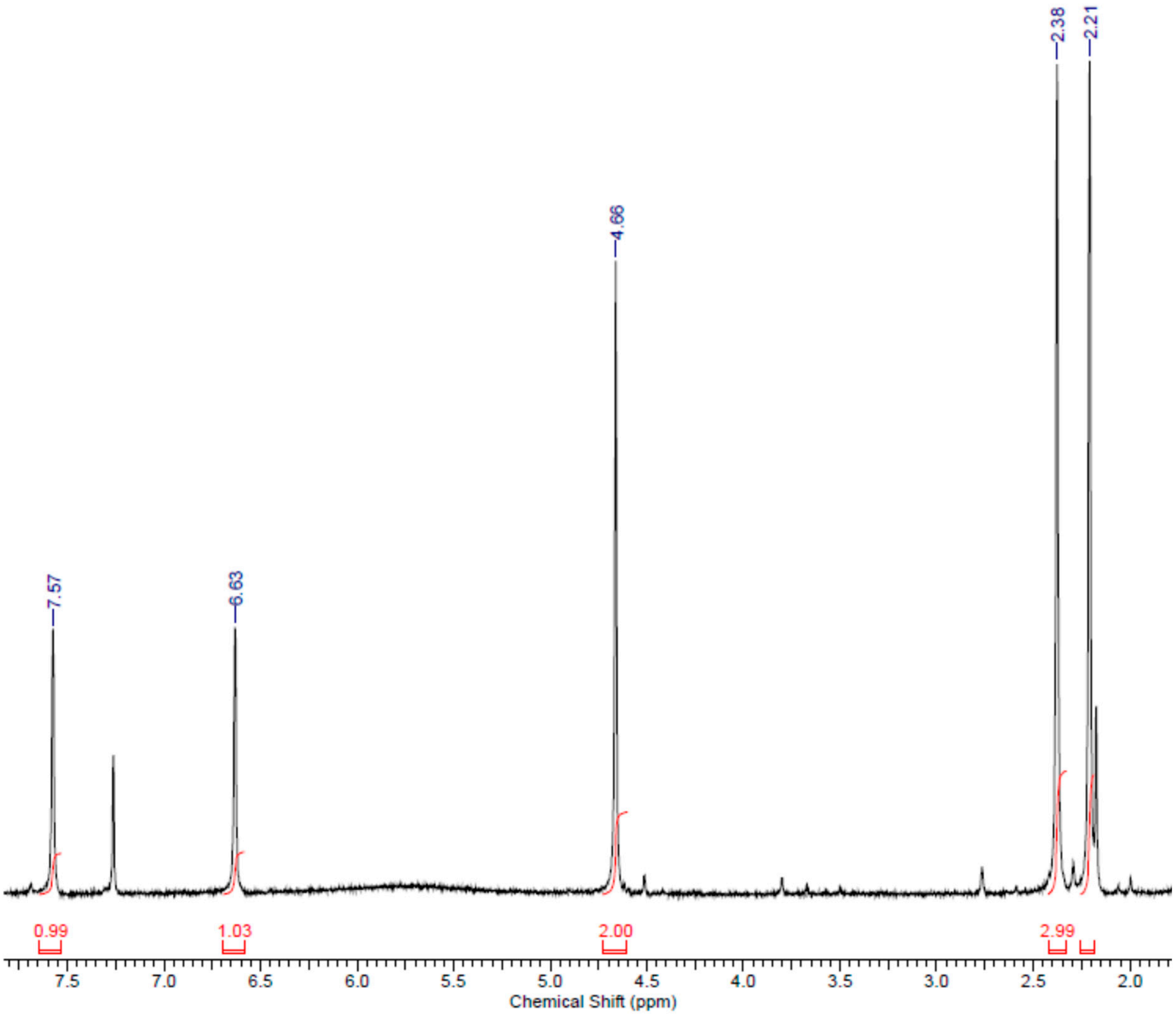

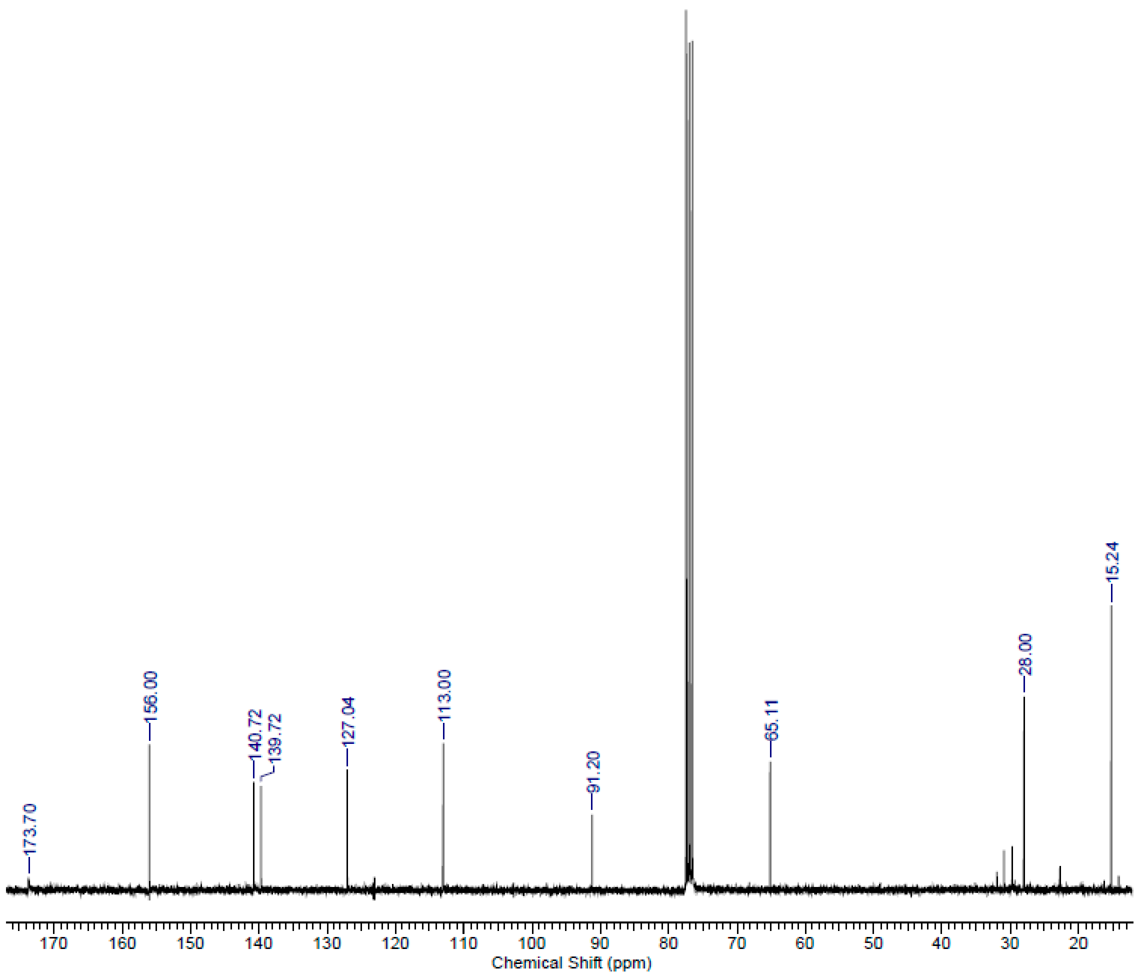

(4- iodo -3,5-dimethylphenoxy)acetic acid **19**

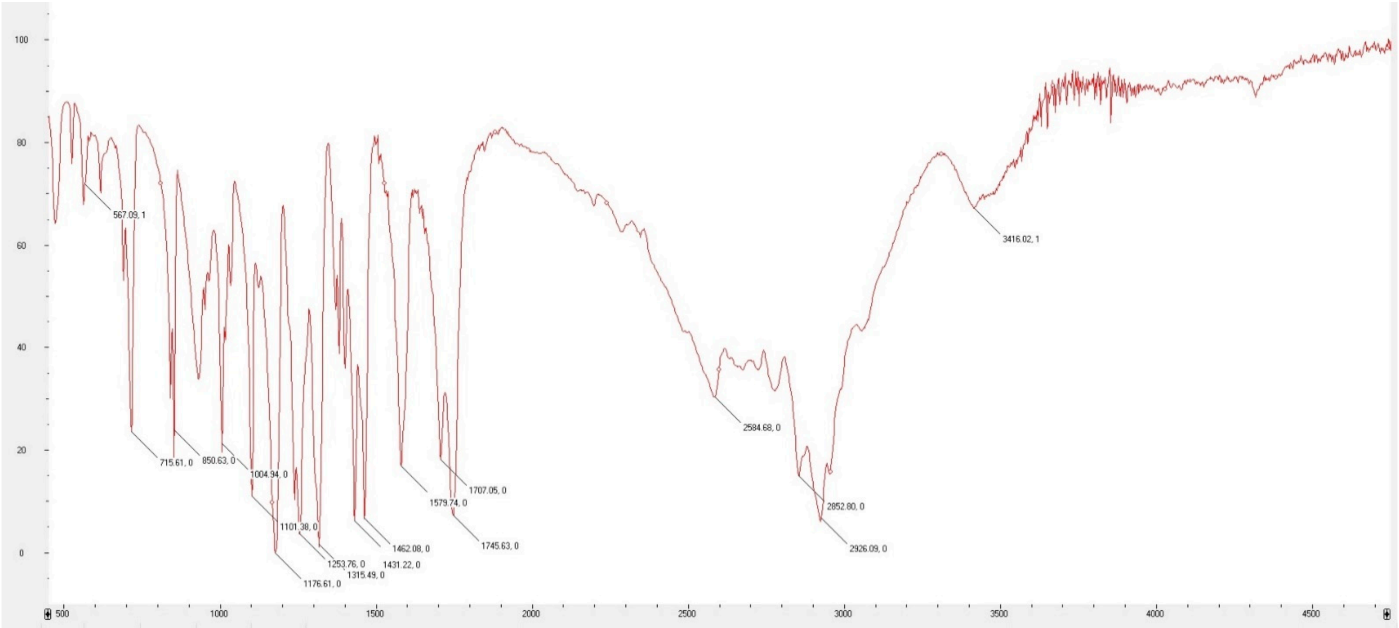

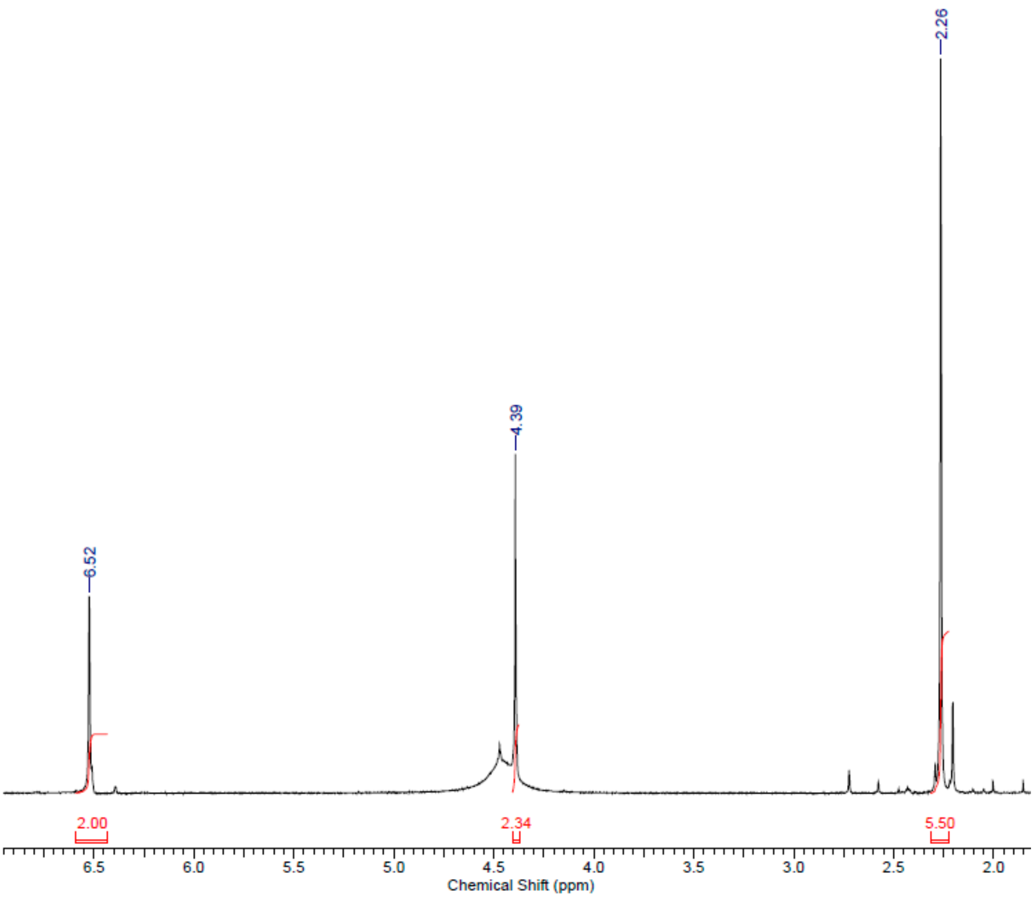
6.52
4.39
2.26
2.00
2.34
5.50
6.5
6.0
5.5
5.0
4.5
4.0
3.5
3.0
2.5
2.0
Chemical Shift (ppm)

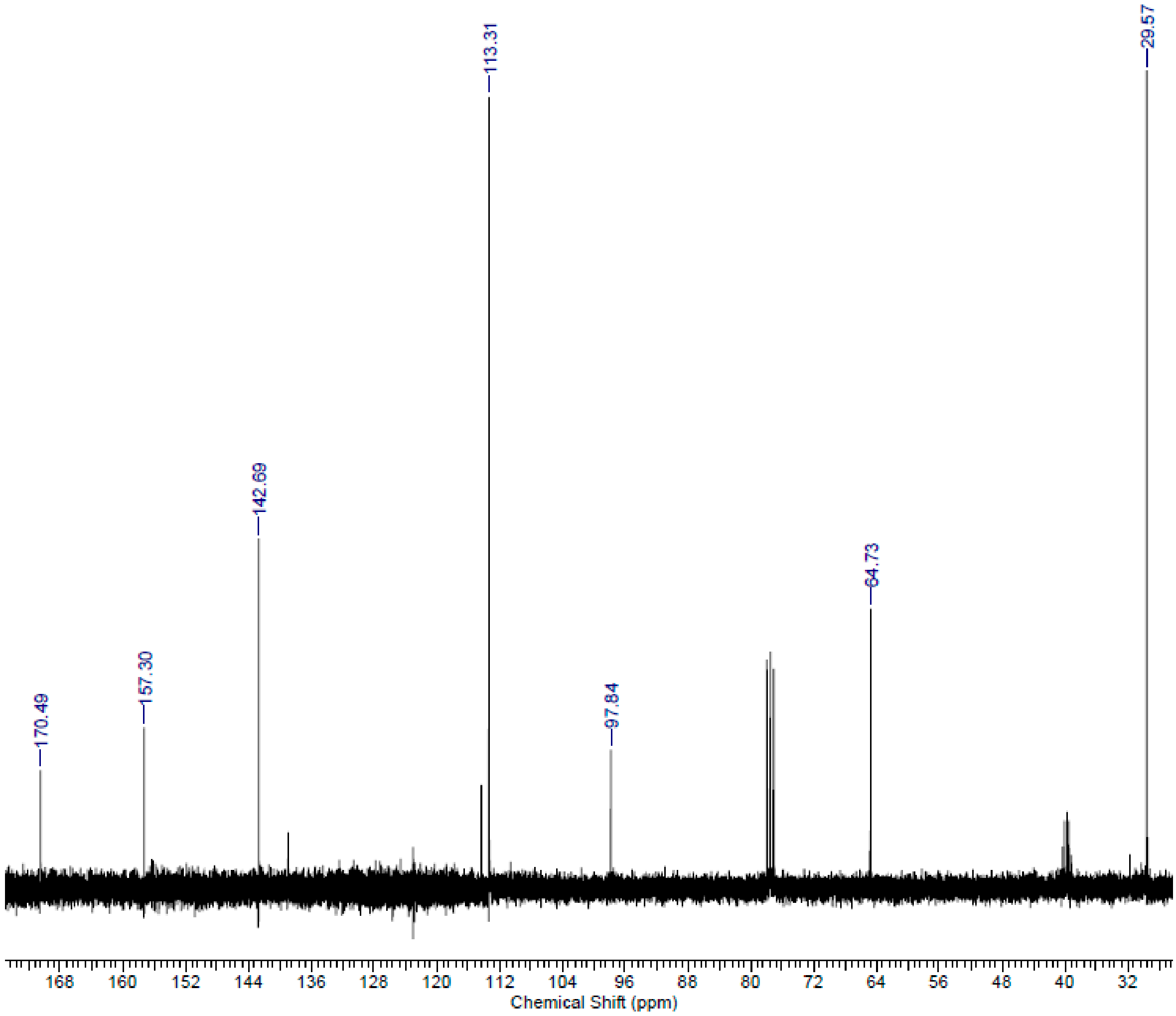
170.49
157.30
142.69
113.31
97.84
64.73
29.57
168
160
152
144
136
128
120
112
104
96
88
80
72
64
56
48
40
32
Chemical Shift (ppm)

(4- iodo -2,3,5-trimethylphenoxy)acetic acid **20**

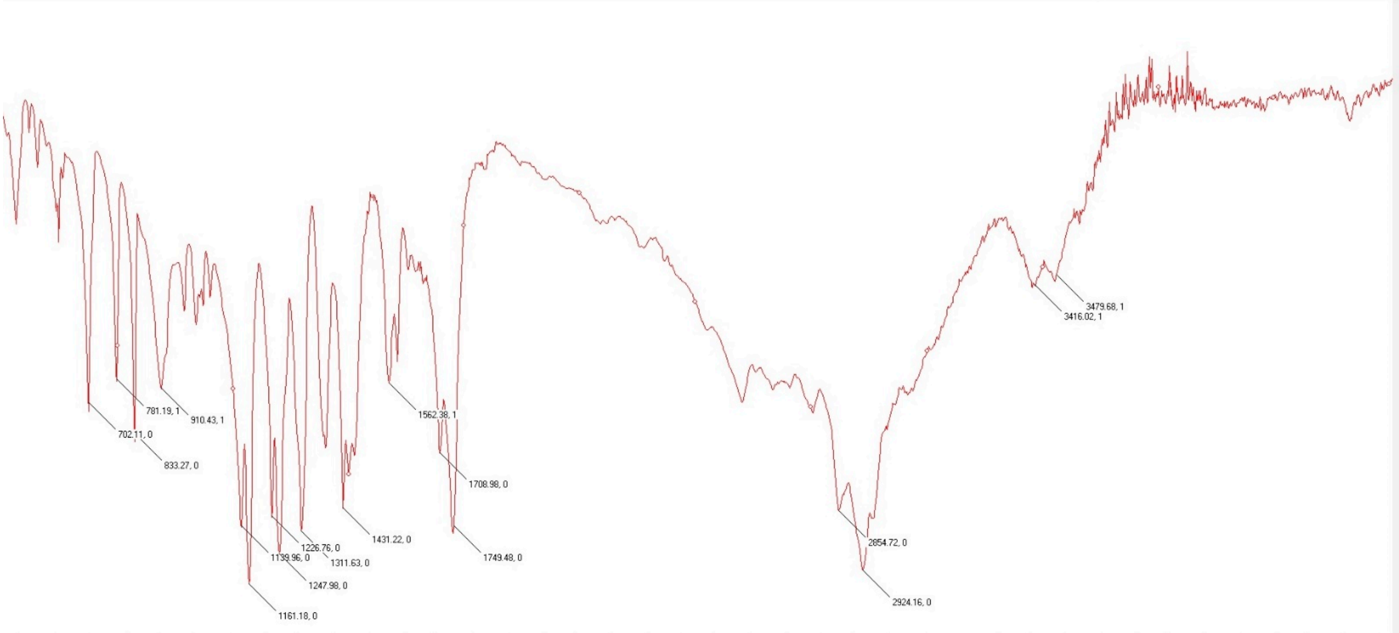

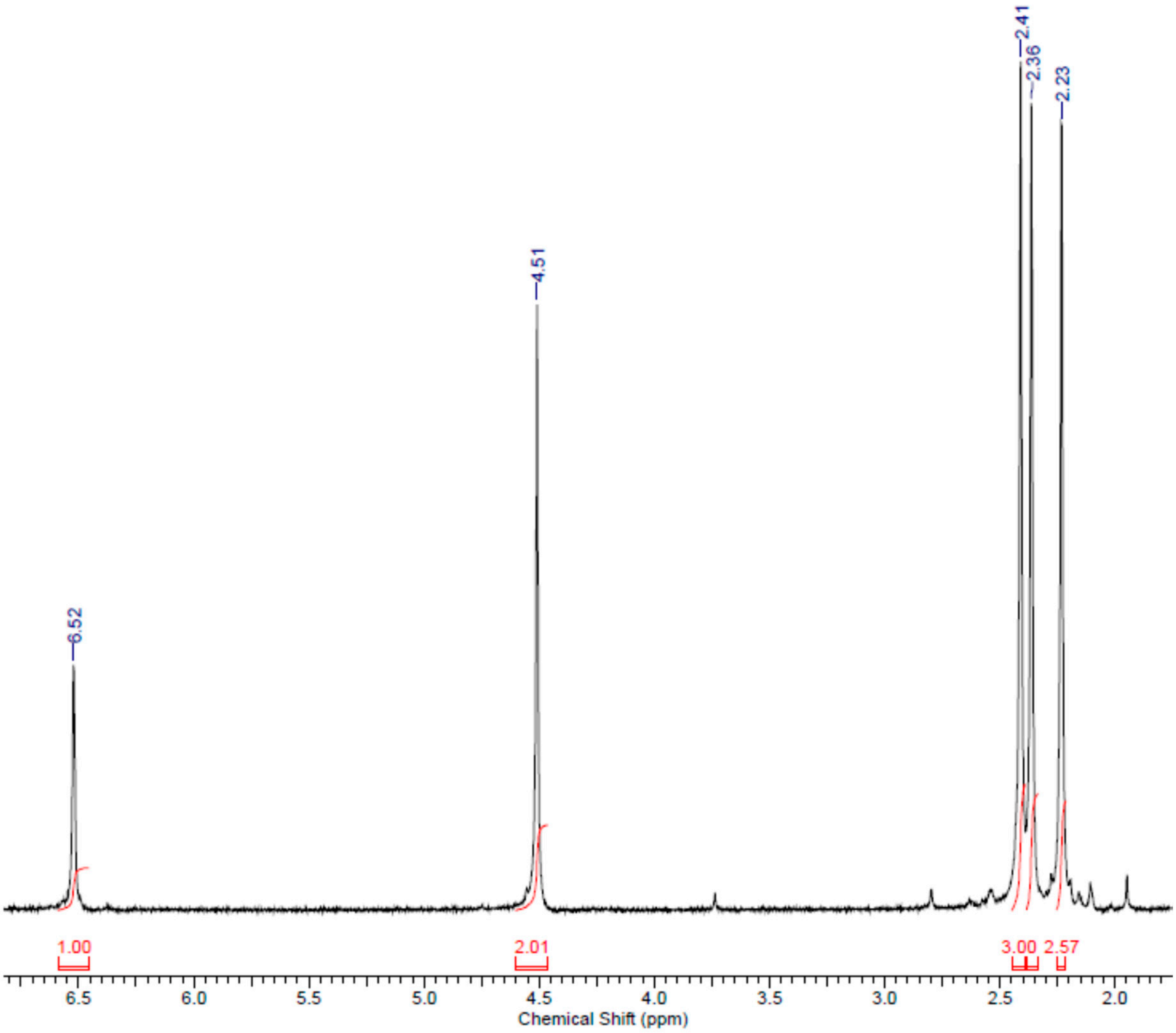

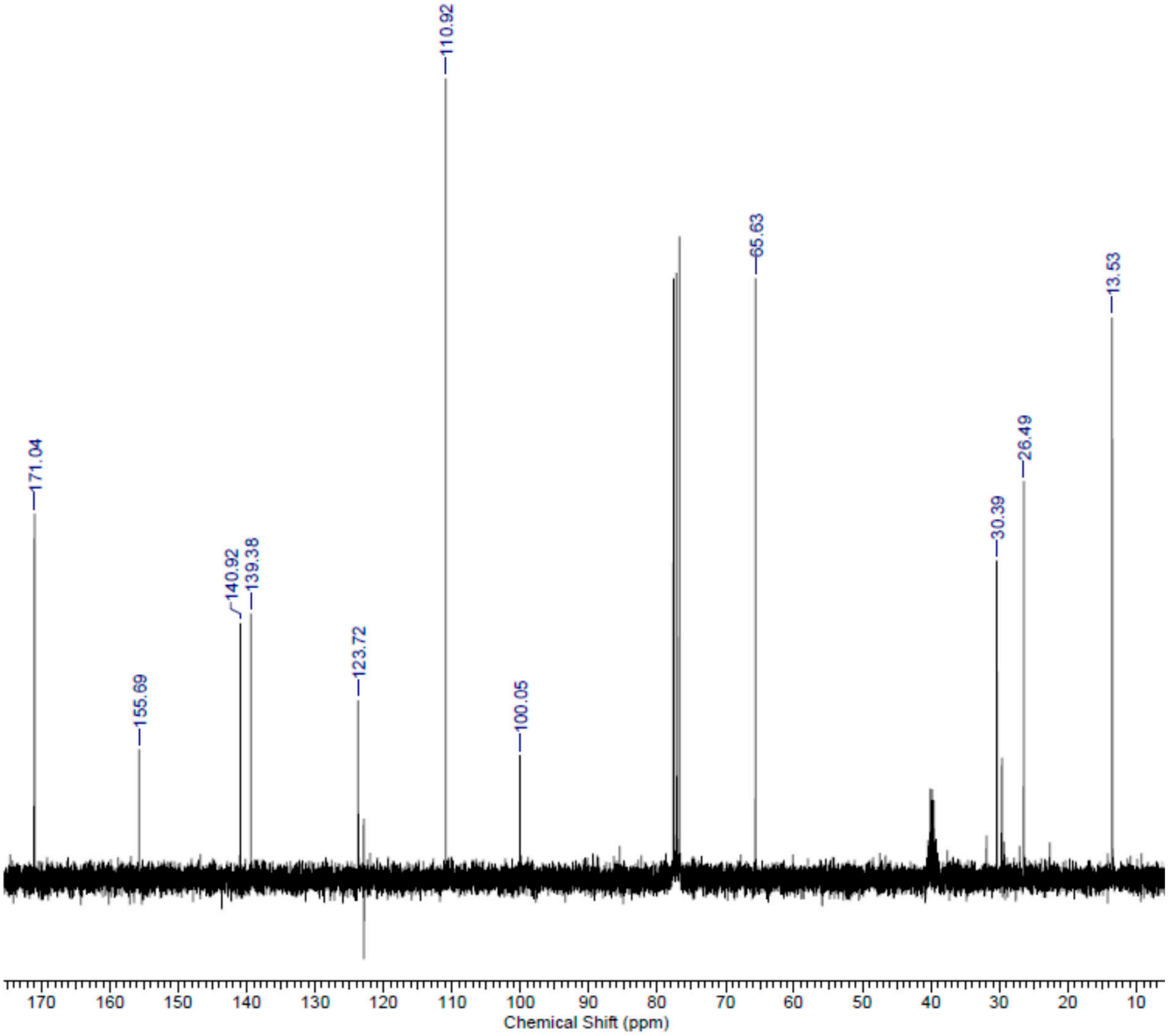

171.04
155.69
140.92
139.38
123.72
110.92
100.05
65.63
30.39
26.49
13.53
170 160 150 140 130 120 110 100 90 80 70 60 50 40 30 20 10
Chemical Shift (ppm)
